# Cysteine-Rich Receptor-Like Kinase Gene Family Identification in the *Phaseolus* Genome and Comparative Analysis of Their Expression Profiles Specific to Mycorrhizal and Rhizobial Symbiosis

**DOI:** 10.3390/genes10010059

**Published:** 2019-01-17

**Authors:** Elsa-Herminia Quezada, Gabriel-Xicoténcatl García, Manoj-Kumar Arthikala, Govindappa Melappa, Miguel Lara, Kalpana Nanjareddy

**Affiliations:** 1 Ciencias Agrogenómicas, Escuela Nacional de Estudios Superiores Unidad León—Universidad Nacional Autónoma de México (UNAM), C.P. 37684 León, Mexico; qrelsa@gmail.com (E.-H.Q.); garxga@gmail.com (G.-X.G.); manoj@enes.unam.mx (M.-K.A.); 2 Department of Biotechnology, Dayananda Sagar College of Engineering, Shavige Malleshwara Hills, Kumaraswamy Layout, Bengaluru 560 078, India; drgovindappa-bt@dayanandasagar.edu; 3 Departamento de Biología Molecular de Plantas, Instituto de Biotecnología, Universidad Nacional Autónoma de México (UNAM), C.P. 62271 Cuernavaca, Mexico; mflara@ibt.unam.mx

**Keywords:** common bean, CRKs, Cysteine (C)-rich receptor-like kinases, genome-wide identification, legume, mycorrhizal fungi, *Phaseolus*, *Rhizobium*, RLK

## Abstract

Receptor-like kinases (RLKs) are conserved upstream signaling molecules that regulate several biological processes, including plant development and stress adaptation. Cysteine (C)-rich receptor-like kinases (CRKs) are an important class of RLK that play vital roles in disease resistance and cell death in plants. Genome-wide analyses of *CRK* genes have been carried out in *Arabidopsis* and rice, while functional characterization of some CRKs has been carried out in wheat and tomato in addition to *Arabidopsis*. A comprehensive analysis of the *CRK* gene family in leguminous crops has not yet been conducted, and our understanding of their roles in symbiosis is rather limited. Here, we report the comprehensive analysis of the *Phaseolus* *CRK* gene family, including identification, sequence similarity, phylogeny, chromosomal localization, gene structures, transcript expression profiles, and in silico promoter analysis. Forty-six CRK homologs were identified and phylogenetically clustered into five groups. Expression analysis suggests that *PvCRK* genes are differentially expressed in both vegetative and reproductive tissues. Further, transcriptomic analysis revealed that shared and unique *CRK* genes were upregulated during arbuscular mycorrhizal and rhizobial symbiosis. Overall, the systematic analysis of the *PvCRK* gene family provides valuable information for further studies on the biological roles of *CRKs* in various *Phaseolus* tissues during diverse biological processes, including *Phaseolus*-mycorrhiza/rhizobia symbiosis.

## 1. Introduction

Plants encounter many environmental cues during their lifespan. The sessile nature of plants exposes them to a broad range of pathogens, nematodes and symbionts. Plants have developed sophisticated mechanisms to defend themselves against pathogenic and parasitic attacks by evoking their immune responses; however, they establish symbiotic associations with the microorganisms via an equally efficient strategy. The plasma membrane localized receptor-like kinases (RLKs) are the key players in perceiving and transducing these external stimuli to further activate the associated downstream signaling pathways. For instance, RLK elicitation by pathogen/microbe-associated molecular patterns (PAMPs/MAMPs), such as bacterial flagellin or fungal chitin heptamers and octamers, or of host-derived damage-associated molecular patterns (DAMPs), activate the defense pathway. During symbiosis, rhizobia derived lipochito-oligosaccharide (LCO) signals (nodulation Factors) and arbuscular mycorrhizal fungi secreted LCO [1] and short-chain chitin oligomer signals (Cos) [2] (Myc factors) bind to the symbiosis specific RLKs that trigger symbiosis signaling. RLKs are categorized into several sub-families, including leucine-rich repeat RLKs (LRR-RLKs), cysteine-rich repeat (CRR) RLKs (CRKs), domain of unknown function 26 RLKs, S-domain RLKs, and others [3].

Cysteine (C)-rich receptor-like kinases (CRKs), also known as DUF26 RLKs, are a large sub-family of plant RLKs. CRKs have a typical RLK domain structure, i.e., they contain an extracellular domain responsible for signal perception, a single-pass transmembrane domain, and a conserved intracellular serine/threonine (Ser/Thr) protein kinase domain responsible for signal transduction. Most CRKs possess two copies of DUF26 in their extracellular domain. The DUF26 domain contains three conserved cysteine residues in a C-X_8_-C-X_2_-C configuration [4]. The cysteine residues in each DUF26 domain are predicted to form two cysteine bridges, which are hypothesized to be targeted for apoplastic redox modification [5]. The structure of CRKs suggests the role of the extracellular domain in the perception of extracellular ligand and transmitting the signal to intracellular kinase domains. There are over 40 *CRK* genes in rice [6] and 44 *CRK* members in *Arabidopsis* [4,7]. The DUF26 domain is found in at least 50 secreted *Arabidopsis* proteins and in eight *Arabidopsis* plasmodesmata-located proteins (PDLPs) [5,8]. Unlike CRKs, the PDL proteins resemble receptor-like proteins, but without the intracellular kinase domain. PDLPs are involved in regulating important cellular processes such as plant cell-to-cell communication, viral cell-to-cell movement, and plant immunity [8,9,10].

CRKs are transcriptionally induced in response to abiotic stress conditions such as salicylic acid, ozone, UV light, drought, and salt treatments [5,11,12,13,14]. Likewise, a group of CRKs are also found to specifically respond to pathogens and PAMP treatments [5,13]. Overexpression of *Arabidopsis CRK4*, *CRK6*, and *CRK36* enhanced the activation of early and late PAMP-triggered immunity (PTI) responses and enhanced resistance to the bacterial pathogen *Pseudomonas syringae* pv *tomato* [14]. Overexpression of *CRK4, CRK5, CRK13, CRK19,* and *CRK20* leads to hypersensitive response-associated cell death in transgenic *Arabidopsis* [11,12,15]. *CRK7* has been reported to mediate the responses to extracellular reactive oxygen species production [16]. Recent reports suggest involvement of *Arabidopsis CRK28* and *CRK29* responsible for cell death in association with membrane receptor like protein kinase BAK1 in response to *Pseudomonas syringae* infection [17]. *CRK* family members in *Glycine max* were found to be transcriptionally regulated by the biotic stress signals triggering plant immune response [18]. Further, *CRK18* in *Gossypium barbadense* is reported to confer resistance to verticillium wilt resistance [19].

Several RLKs have been implicated in legume symbiosis [20,21,22,23,24,25]. However, *SymCRK* in *Medicago truncatula* is the only known *CRK* with a role during symbiosis [26,27]. Although recent years have seen considerable information being generated to understand the gamut of activities of CRKs, little is being done towards understanding the role of *CRK* genes during legume symbiotic associations.

In the present work, we identified the *CRK* gene family members in *Phaseolus vulgaris* and compared their expression levels specific to mycorrhizal and rhizobial symbiosis. Further, a detailed analysis of chromosomal localization and phylogenetic relationship among them was carried out. Towards an attempt to understand the phylogenetic distribution of *PvCRK* family members, gene structural analysis, i.e., intron–exon structure, protein secondary structure, conserved motifs, transmembrane helices, and hydrophobicity were studied. Further, the putative function of each *PvCRK* was predicted based on the *cis*-acting elements of the promoters. Expression patterns of *PvCRK* members in various *Phaseolus* tissues were studied. Finally, *CRK* expression patterns from mycorrhiza/*Rhizobium* inoculated *Phaseolus* roots are studied using previously generated RNA sequencing (RNA-Seq) data. With these analyses, we provide fundamental data for *CRK* family genes in *P. vulgaris* that could be further applied to studying various biological aspects of CRKs in *Phaseolus,* including symbiosis.

## 2. Materials and Methods

### 2.1. Identification, Alignment and Phylogenetic Analysis of CRK Orthologs

The *Arabidopsis CRK* gene family sequences [4] were used as query sequences in BLASTN and BLASTP searches of *CRK* homologs in *P. vulgaris, G. max, M. truncatula, Oryza sativa* and *Zea mays* in the Phytozome genome database (https://phytozome.jgi.doe.gov) using default settings for *e*-value and the number of hit sequences. The genome versions used for different species were *P. vulgaris* v2.1, *Z. mays* PH207 v1.1, *M. truncatula* Mt4.0v1, *O. sativa* v7_JGI and *G. max* Wm82.a2.v1. In addition to Phytozome, various other genome databases were used for the retrieval of *CRK* homologs, including Legume Information System (https://legumeinfo.org) for legumes, Lotus Base (https://lotus.au.dk) for *Lotus japonicus*, and OrthoMCL (http://orthomcl.org) for *L. japonicus* and *O. sativa*. The respective nucleic acid and peptide sequences were downloaded from the online tool PhytoMine from the plant comparative genomics portal Phytozome v12.1 for further analysis and annotation. Obtained genes and protein sequences were further examined to include the conserved domains by querying using Uniprot [28] and Pfam [29] databases.

Finally, the putative CRK homologs for each species were filtered using conserved sequence motif analyzer MEME [30] (http://meme-suite.org) and signal peptide cleavage site predictor SignalP v4.1 [31] (http://www.cbs.dtu.dk/services/SignalP/). Multiple sequence alignment of intra-species and *Phaseolus* CRK peptide sequences was performed using ClustalW. Phylogenetic analysis of the aligned sequences was carried out using Molecular Evolutionary Genetics Analysis (MEGA) 7.0 with the neighbor-joining (NJ) method, the JTT+I+G substitution model with 1000 bootstrap replicates keeping the default parameters [32]. Multiple sequence alignment of CRK sequences from individual groups was carried out using ClustalW and further, sequence identity was determined using Sequence Manipulation Suite (http://www.bioinformatics.org/sms2/ident_sim.html).

The chromosomal localization of *P. vulgaris CRK* gene family members were verified from the Phytozome v12.1 database, chromosomal images were drawn using EnsemblPlant tool [33], the centromere positions were designed according to Fonsêca et al. [34] and scale was determined based on Wang et al. [35].

### 2.2. Analysis of Exon-Intron Structures and Conserved Motif Identification in PvCRK Genes

The exon-intron organization of 46 *CRK* genes of *Phaseolus* was analyzed using Gene Structure Display Server (GSDS) (http://gsds.cbi.pku.edu.cn/index.php) [36]. The conserved motifs of PvCRK family members were determined using Multiple Em for Motif Elicitation (MEME) 4.11.4 (http://meme-suite.org/tools/meme). Genes with an *e*-value of <1 × 10^−20^ were subjected to further analysis. The motif representation was made with MAST version 4.12.0 ordered by *p*-values. The motifs obtained were analyzed with the BLASTP interface at National Center for Biotechnology Information (NCBI) website (https://blast.ncbi.nlm.nih.gov/Blast) and Pfam 31.0 database [37]; each was represented by multiple sequence alignments and hidden Markov models (HMMs).

The secondary structures of CRK proteins were predicted by MLRC (Multivariate Linear Regression Combination methods) using SOPMA-GOR4-SIMPA and run in NPS@ server (Network protein sequence analysis) [38]. The output results use the DSSP (Dictionary of protein secondary structure) to describe the structures such as α-helix (Hh) with minimum length 4 residues, extended strand (Ee) in parallel and/or anti-parallel β-sheet conformation.

### 2.3. Transmembrane Helices and Hydrophobicity Analysis of PvCRK Proteins

Prediction of transmembrane helices of the *P. vulgaris* CRK proteins was achieved by Phobius (http://phobius.sbc.su.se/) [39] server utilizing peptide sequences. The hydropathic character of the CRK proteins was analyzed using ProtScale (https://web.expasy.org/protscale/) according to Kyte and Doolittle [40]. The average of hydropathicity for the 46 CRKs was analyzed with ProtParam (https://web.expasy.org/protparam/) utilizing the scoring criteria of Gasteiger et al. [41]. The grand average of hydropathicity (GRAVY) value for the proteins was calculated as described by Miao et al. [42].

Next, Plant-mPLoc tool (http://www.csbio.sjtu.edu.cn/bioinf/plant/) was used to identify the sub-cellular localization of proteins in various cellular organelles and pathways. Plant-mPLoc identifies a wide variety of location sites such as cell membrane, cell wall, chloroplast, cytoplasm, endoplasmic reticulum, extracellular, Golgi apparatus, mitochondrion, nucleus, peroxisome, plastid, and vacuole [43].

### 2.4. Promoter Analysis of CRK Genes and GO Annotation

The promoter regions 2000 bp sequences upstream of coding region of 46 *CRK* genes were downloaded from Phytozome v12.1, *P. vulgaris* genome database. In silico analysis of promoter sequences was performed using PlantCARE software [44] to identify the *cis*-regulatory elements of CRK promoters. The frequency of the motifs found on each CRK promoter was represented as a pie chart using the R package (https://www.r-project.org/).

The Gene Ontology (GO) enrichment analysis was performed using AgriGO and REVIGO online tools (http://bioinfo.cau.edu.cn/agriGO/) [45]. GO categories (molecular function, biological process and cellular component) were developed using 46 CRK IDs of *P. vulgaris*. The results are represented graphically.

### 2.5. Transcriptome Profiling and RT-qPCR Analysis

Previously, we performed global transcriptome profiling in *Phaseolus vulgaris* L. cv. *Negro Jamapa* roots colonized with *Rhizophagus irregularis* spores, or *Rhizobium tropici* strain CIAT899 [46]. The present study utilizes the same transcriptomic data to obtain the expression profiles of *CRK* family genes under both types of symbiotic conditions. Heat maps were constructed with fold-change values applying the R package (https://www.r-project.org/). Venn diagrams were drawn with differentially expressed gene (DEG) numbers using a Venn diagram drawing tool (http://bioinformatics.psb.ugent.be/webtools/Venn/).

To validate the RNA-seq data, we surface-sterilized *P. vulgaris* L. cv. *Negro Jamapa* seeds and germinated them as described by Nanjareddy et al. [47]. Two-day-old germinated seedlings were transplanted into sterile vermiculite and were inoculated with *R. irregularis* or *R. tropici* according to Nanjareddy et al. [46]. Uninoculated *P. vulgaris* plants served as controls. Root tissues were separated from the shoots at two-week post inoculation, and were immediately frozen in liquid nitrogen and stored at −80 °C until they were subjected to RNA extraction. The root tissues were ground in liquid N_2_, and total RNA was extracted using the Spectrum™ Plant Total RNA Kit (Merck KGaA, Darmstadt, Germany) following the manufacturer’s recommendations. DNA contamination in the RNA samples were eliminated by incubating the samples with RNase-free DNase (1 U µL^−1^) at 37 °C for 15 min and then at 65 °C for 10 min. RNA integrity was verified by electrophoresis, and the concentration was assessed using a NanoDrop^TM^ 2000 spectrophotometer (ThermoFisher Scientific, Wilmington, DE, USA). DNA-free RNA samples were used in quantitative real-time PCR assays, which were performed using the iScript^TM^ One-step RT-PCR Kit with SYBR^®^ Green, following the manufacturer’s recommendations in an iQ^TM^ 5 (Bio-Rad, Hercules, CA, USA). Each reaction was prepared with 40 ng of RNA as template. A control sample lacked reverse transcriptase (RT) was incorporated to confirm the absence of contaminant DNA. Relative expression values were calculated using the formula 2^−ΔCT^, where cycle threshold value (ΔCT) is the CT of the gene of interest minus CT of the reference gene. Reference genes *viz., EF1α* and *IDE* [48,49] were used to normalize the expression data [50]. The gene-specific oligonucleotides used in this study are listed in Supplementary Table S1.

## 3. Results

### 3.1. Identification and Phylogenetic Analysis of the CRK Gene Family in Phaseolus Vulgaris

Genome-wide identification of *CRK* genes in *P. vulgaris* was performed based on homology with the identified *Arabidopsis CRK* family genes [5,13]. BLASTN and BLASTP searches were extensively employed to identify *P. vulgaris* CRK homologs from the *P. vulgaris* v2.1 genome database. A total of 46 *CRK* gene family members were identified in the genome of *P. vulgaris* (Table 1). HMMs of PvCRK proteins were determined based on the presence of two DUF26 domains in the Pfam database. The CRK family members were named according to their chromosome position starting from chromosome one to eleven. The numbering was from the short arm towards the long arm, starting from proximal to distal ends of the respective arms. The length and molecular weight (Mw/Da) of the deduced CRK proteins ranged from 376 to 1072 amino acids and 119.67 kDa to 42.98 kDa, respectively. The theoretical isoelectric point (pI) of most PvCRKs was slightly acidic (4.92–6.95), and twelve CRK proteins were alkaline (7.01–8.11) (Table 1).

**Figure 1:**
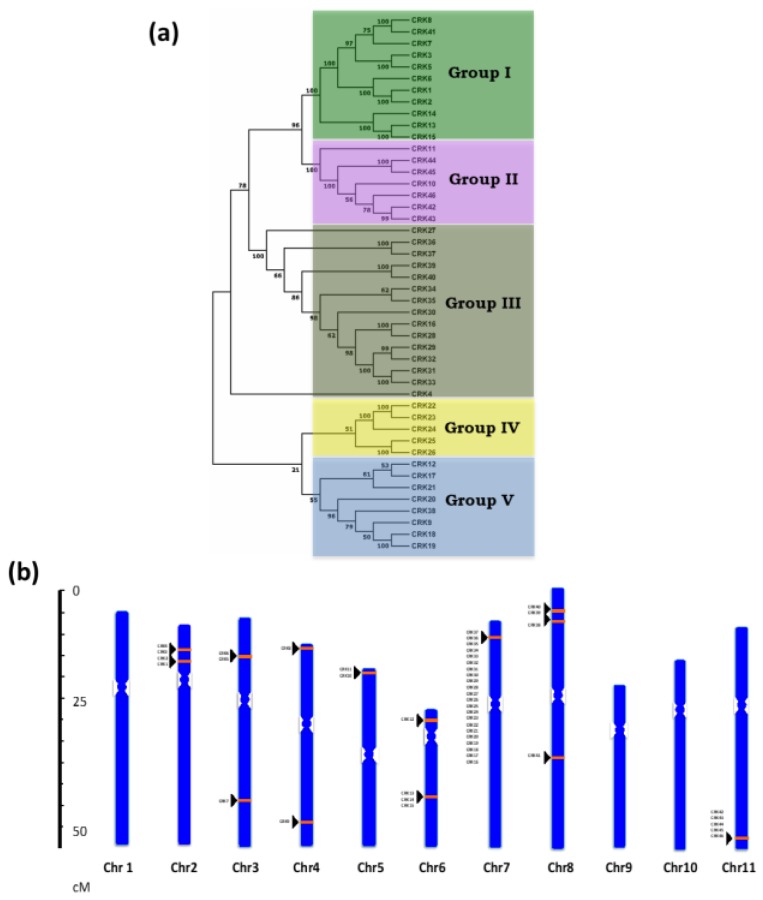
Phylogenetic analysis and chromosomal distribution of *Phaseolus vulgaris CRK* genes. (**a**) Protein sequences of 46 *P. vulgaris* Cysteine (C)-rich receptor-like kinase (CRK) homologs were identified in the Phytozome database. The phylogenetic tree was constructed using MEGA 7 software with the neighbor-joining (NJ) tree method with 1000 bootstrap values. (**b**) *CRK* genes localized to *Phaseolus* chromosomes. The chromosomes are represented by blue bars that are distributed numerically. The orange bands with black triangles indicate the *CRK* position on the chromosome.

Phylogeny of *P. vulgaris* CRK genes was constructed using a NJ method that classified the CRK homologues into two major clusters. One major cluster was divided into three minor clusters/groups and the other into two minor clusters/groups. Hence, the phylogenetic alignment was classified into five groups (Figure 1A). Interestingly, among these groups, 19 out of 22 CRKs localized on chromosome 7 fell into group III and IV. Next, chr11 had a maximum of five CRKs from *CRK42* to *CRK46*, which came under group II of the phylogenetic tree (Figure 1B). Sequence identity of CRK members in each group was found to be 46.5%, 65.15%, 70.29%, 52.37%, and 49.3% in Group I, II, III, IV, and V, respectively.

To investigate the evolutionary relationship between *Phaseolus* CRK proteins and CRKs from other species, a joining–joining method phylogenetic tree was constructed based on full amino acids of CRK family proteins from *P. vulgaris*, *M. truncatula*, *G. max*, *L. japonicus*, *O. sativa*, *Z. mays,* and *A. thaliana*. The dendrogram showed that the 280 CRKs (Table S2) could be classified into 14 distinct groups based on their sequence similarity (Figure S1).

### 3.2. Localization of CRK Gene Members on Phaseolus vulgaris Chromosomes

A total of 46 *PvCRK* genes were mapped to 8 of the 11 *P. vulgaris* chromosomes. The distribution and density of *PvCRK* genes on chromosomes were not uniform (Figure 1B). Among the 11 chromosomes of *P. vulgaris*, chr04, and chr05 contained two *CRK* genes and chr03 had three genes. Four *CRK* genes were localized on chr02 and chr08, five genes on chr06 and six genes on chr11. Interestingly, chr07 had a region with high gene density on the short arm, probably due to local gene duplications. The 22 *PvCRK* genes were spread over a genomic region of ~320 kb on chr07, forming the largest *CRK* gene cluster. *CRK* genes were absent on chr01, chr09, and chr10. Approximately, 70% of *CRK* genes were localized on chromosome short arms (Figure 1B).

### 3.3. Structural Analysis of CRK Genes

**Figure 2:**
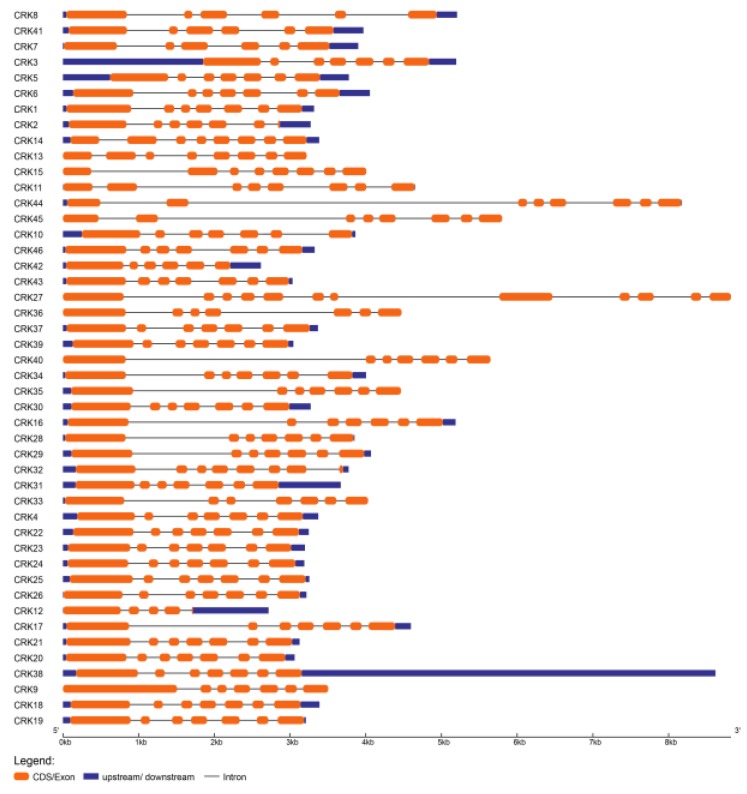
Gene structure analysis of *Phaseolus* cysteine-rich receptor-like kinases (CRKs). The intron–exon structures of *PvCRK* genes were analyzed using the Gene Structure Display Server (GSDS) database. Exons/Coding sequence (CDS) are represented by orange bars, introns by grey lines, and upstream (5′)/downstream (3′) untranslated regions (UTRs) are blue bars.

To understand the structural features of *Phaseolus* CRKs, we analyzed intron–exon distribution and conserved motifs. Intron–exon location analysis using the GSDS database showed that the number and distribution of intron–exon locations were highly conserved among the CRK homologs in *P. vulgaris* (Figure 2). The CRKs exhibited a range of 4 to 12 exons per gene; among them, 29 genes had 7 exons per gene and most of these also had a conserved distribution and length for each exon. Of 29 CRKs with 7 exons, 20 were localized on chr07 from *CRK16* to *CRK37,* except for *CRK32* with 8 exons and *CRK27*, which had a maximum of 12 exons. Phylogeny of these CRKs also showed grouping of these genes together into group III, IV and V (Figure 1A). In addition, 6 CRKs, *CRK11*, *CRK13*, *CRK14, CRK15, CRK44,* and *CRK45,* have 8 exons, and *CRK7, CRK8, CRK41,* and *CRK42* have 6 exons each. The *CRK* genes that contain 8 and 6 exons were clustered into group I and group II of the phylogenetic tree. *CRK12* had at least 5 exons (Figure 2). However, *CRK27* and *CRK44* had the longest genomic and protein sequence among the 46 *PvCRK* genes (Figure 1A and Figure 2).

The secondary structure prediction of the 46 CRKs in *P. vulgaris* revealed that 29.83% are α-helix, 18.51% are extended strand and 51.65% are random coil. The highest percentage of α-helix was contained in *CRK16* with 40.92%, while *CRK30* showed the lowest amount, with 26.15% α-helix. *CRK46* had the highest percentage of extended strand with 24.19% and *CRK19* had the lowest with 14.99% (Figure S2; Table S3).

### 3.4. Transmembrane Regions of CRK Proteins

Transmembrane helices were predicted for the 46 CRK proteins in *P. vulgaris* with the Phobius (http://phobius.sbc.su.se/) server. All the 46 CRK members of *Phaseolus* were found to contain a single transmembrane domain based on the analysis using Phobius, a combined transmembrane topology and signal peptide predictor. The results confirm that the CRK proteins contained only one transmembrane region typical to CRKs and RLKs (Figure S3). Multiple sequence alignment of PvCRK amino acid sequences using ClustalW revealed the highly conserved nature of the transmembrane region in most of the CRKs (Figure S4). The hydrophobicity analysis was carried out to predict whether a peptide segment is sufficiently hydrophobic to interact or reside within the interior of the membrane. The results for the GRAVY outputs showed values ranging from −0.032 to −0.335 for all of the CRK proteins (Table S4). Further, ProtScale analysis was carried out to determine the hydrophobic regions of the CRK proteins (Figure S5); the results agreed with the predicted transmembrane helices (Figure S2).

### 3.5. Signal Peptide Analysis and Subcellular Localization of PvCRK Proteins

The presence and location of signal peptide cleavage sites in amino acid sequences was predicted using SignalP v4.1; based on a combination of several artificial neural networks, it was determined that all 46 PvCRK sequences contained a signal peptide region. The length of signal peptide varied from 19 to 38 amino acids (Figure S3). To investigate the subcellular localization of CRKs, we used Plant-mPLoc software to search localization specific motifs. The analysis suggested that all CRKs identified in *Phaseolus* appeared to be localized to plasma membrane.

### 3.6. Protein Sequence Motif Identification

**Figure 3:**
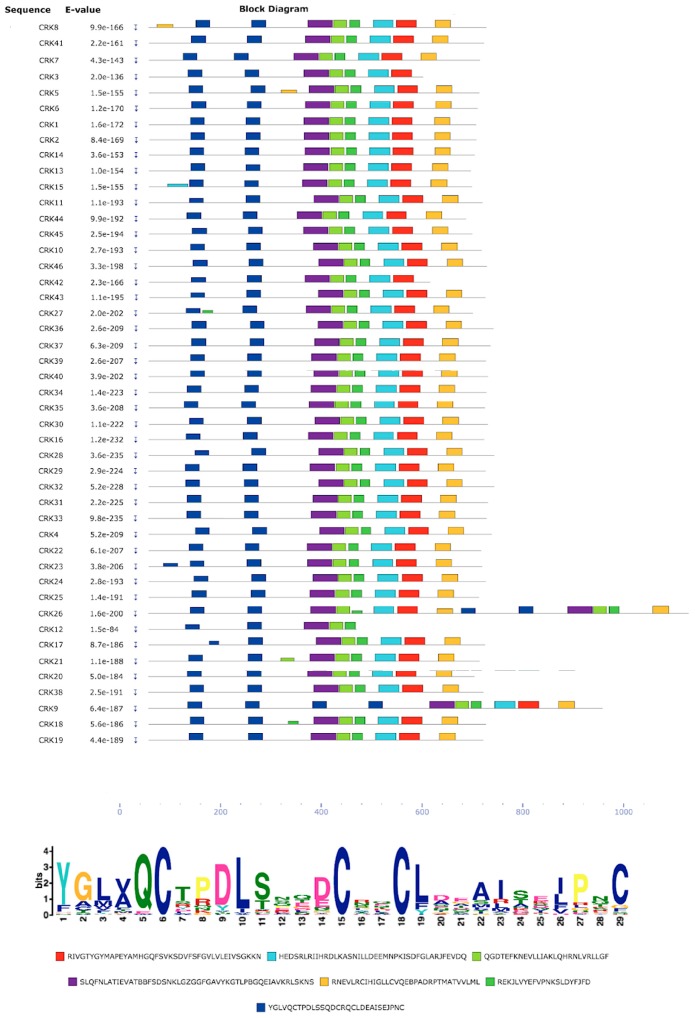
Identification of motifs in CRK protein sequences. MEME was used to identify motifs in the 46 *P. vulgaris* CRKs. Significantly overrepresented motifs are graphically depicted by bars corresponding to their predicted position. The dark blue bars are analogous to salt stress response/antifungal domain (PF01657), and the corresponding sequence logo is shown in the lower section, in which conserved amino acids are represented by one-letter abbreviations. The red boxes represent kinase domains (PF00069) and light blue represents Pkinase_Tyr (PF07714).

Conserved motif analysis of the CRK proteins through the MEME server showed seven motifs (Figure 3). The characteristic motif of CRK proteins is DUF26 (Domain unknown function 26; Pfam: PF001657), and alignment of PvCRK protein sequences showed two DUF26 domains with the conserved sequence C-X_8_-C-X_2_-C in most of the PvCRKs. However, CRK9 and CRK26 contained four DUF26 domains. The DUF26 domain corresponds to a salt stress response/antifungal activity. Pfam search of other domains showed two kinase domains, PF00069 and PF07714. The remaining four motifs showed no match in the Pfam IDs, and hence their functional role is not understood.

### 3.7. CRK Promoter Analysis

To understand the transcriptional regulation and potential function of the *PvCRK* genes, we analyzed the *cis*-regulatory elements in the promoter sequences (Table S5) using PlantCARE software. The results show 99 motifs for the 2000 bp CRK promoter regions (Table S5). Among them, 20 motifs involved in light response elements, such as ACE, AE-box, AT1-motif, ATCT-motif, Box 4, G-box, GA-motif, GT1-motif, TCT-motif, SP1, and I-box, were important, indicating that the *CRK* family genes might participates in photosynthesis activity. Several hormonal responsive elements, i.e., auxin responsive AuxRR-core (2 genes) and TGA-element (13 genes); gibberellin response dOCT (1 gene), GARE-motif (24 genes) and p-box (7 genes); ethylene and ABA responsive ERE (19 genes) and ABRE (19 genes), were also found on the CRK promoter regions (Table S5). Further, CRK promoter regions were also rich in defense, methyl jasmonic acid, and salicylic acid responsive elements. The unique motif ´fungal elicitor responsive element´ was present on 19 CRK promoters. Notably, TATA box and CAAT box motifs were the most predominant *cis*-regulatory elements found in the CRK promoter regions. The motifs present in the promoter regions of the *CRK* genes revealed their essential role in growth and development, and in plant-microbe interactions.

**Figure 4:**
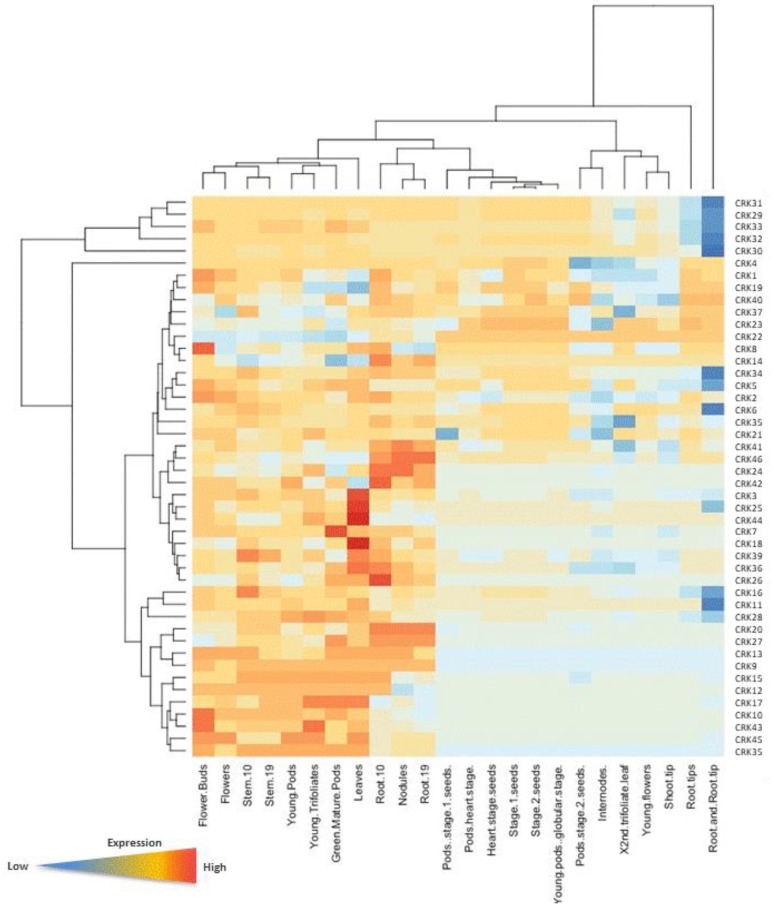
In silico expression profiles of *P. vulgaris* CRKs. Heat map expression profiles of CRK family genes in various tissues of *P. vulgaris*. The transcriptome data across different tissues were extracted by Phytozome (*P. vulgaris* v2.1) and the *P. vulgaris* gene expression atlas (PvGEA). The heat map was generated by R using the Fragments per kilobase of exon model per million reads mapped (FPKM) values of each CRK gene.

Tissue-specific and developmental stage-related expression data provide us with clues about the functions of the *PvCRK* family genes in different vegetative and reproductive tissues of *P. vulgaris*. Therefore, we performed an in silico analysis and extracted the expression levels reported in the Phytozome (*P. vulgaris* v2.1) transcriptome database. Based on the expression profile heat map (Figure 4B), we found that an average of 50% *CRK* genes were downregulated (i.e., Fragments per kilobase of exon model per million reads mapped (FPKM) values > −1.0) in vegetative (roots, stem, leaves, and young trifoliates) and reproductive tissues (flower buds, flower, young pod and mature pod). Upregulated (i.e., FPKM values > 1.0) expression in both vegetative and reproductive tissues was seen in an average of 22% *CRK* genes. However, the change in transcript expression was not detected in an average of 28% CRKs (Figure 4B). Interestingly, the transcript levels of *CRK4, CRK22, CRK23, CRK29, CRK31,* and *CRK40* were highly upregulated, while *CRK9, CRK13, CRK28, CRK38,* and *CRK45* were highly downregulated in all observed tissue types (Figure 4B). Based on most predominant *cis*-regulatory elements found in the CRK promoter regions (Table S5), we selected *CRK16*, *CRK23* and *CRK42* as representative members for the light responsive elements; *CRK2*, *CRK3* and *CRK17* as the defense responsive elements; and *CRK7*, *CRK38,* and *CRK43* as hormonal responsive elements. Next, we performed RT-qPCR analysis for the above selected *CRK* genes in vegetative and reproductive organs of wild-type *Phaseolus* plants. Differential expression patterns of *CRK* genes were observed in different *Phaseolus* organs (Figure S5), and these results were consistent with those observed using the in silico analysis from the Phytozome (*P. vulgaris* v2.1) transcriptome database (Figure 4B). Together, variable expression patterns were observed among the *PvCRK* members, indicating different functions of CRK genes in various tissues of *P. vulgaris*.

### 3.8. Gene Ontology and Validation of Transcriptome Data

**Figure 5:**
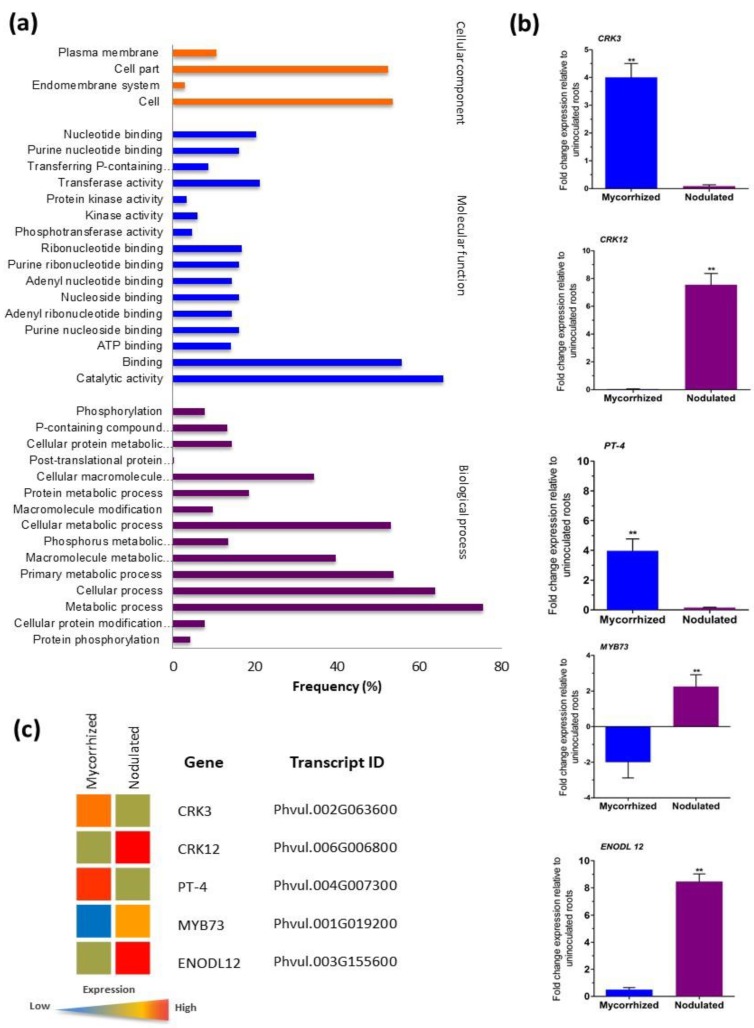
Gene ontology (GO) annotation and RT-qPCR validation of RNA sequencing (RNA-Seq) data from symbiont-colonized *P. vulgaris* roots. (**a**) GO term annotation of PvCRKs were summarized in three main GO categories, biological process, molecular function and cellular component. GO enrichment analysis performed using AgriGO and REVIGO platforms. Bars indicates the frequency of genes with the same term. (**b**) RT-qPCR analysis showing relative expression of *Phaseolus CRK3*, *CRK12*, *PT-4*, *MYB73,* and *ENODL12* genes. Candidate genes were selected and corresponding transcript accumulation under mycorrhized and nodulated conditions was quantified by RT-qPCR. RT-qPCR data are the averages of three biological replicates (n > 9). Statistical significance of differences between mycorrhized and nodulated roots was determined using an unpaired two-tailed Student’s *t*-test (** *p* < 0.01). Error bars represent means ± Standard error mean (SEM). (**c**) Heat map of the transcriptomic data obtained through RNA-Seq showing the expression profiles of *CRK3*, *CRK12*, *PT-4*, *MYB73* and *ENODL12*. Color key in red and blue color represents upregulated and downregulated genes respectively whereas, yellow represents no transcript accumulation.

Gene ontology (GO) was used to classify the 46 CRK genes of *P. vulgaris* into functional groups using AgriGO and REVIGO platforms. Our results show that the CRKs were allocated to three GO categories: biological process, cellular component and molecular function (Figure 5A). Maximum numbers of CRKs were assigned to molecular function (46%), followed by biological processes (43%) and cellular components (11%). In the molecular function category, catalytic activity (GO-0003824), binding (GO-0005488), transferase activity (GO-0016740), and nucleotide binding (GO-0000166) were the most highly represented GO terms. Minor subgroups in the molecular function category included protein kinase activity (GO-0004672), kinase activity (GO-0016301), and phosphotransferase activity (GO-0016773) and transferase activity: transferring phosphorus-containing groups (GO-0016772). In the biological process category, primary metabolic process (GO-0044238), metabolic process (GO-0008152), cellular metabolic process (GO-0044237), cellular process (GO-0009987), cellular macromolecule metabolic process (GO-0044260), and macromolecule metabolic process (GO-0043170) were the most abundant terms. Minor groups within this category included macromolecule modification (GO-0043412), protein phosphorylation (GO-0006468), phosphorylation (GO-0016310), and cellular protein modification process (GO-0006464). In the cellular component category, the most abundant groups were plasma membrane (GO-0005886), cell (GO-0005623) and cell part (GO-0044464). Endomembrane system (GO-0012505) was the only minor group found within this category. The GO functional annotations of the CRKs suggest that the members of the gene family were distributed among several important groups of all three GO categories at distinctive percentages.

Previously, we performed *P. vulgaris* RNA-seq analysis of mRNA from uninoculated control and 2 weeks post inoculation with mycorrhized or nodulated roots using Ion Proton sequencing; data obtained were then deposited in the NCBI with accession number of PRJNA388751 (https:// www.ncbi.nlm.nih.gov/bioproject/388751). In total, 1959 upregulated and 1260 downregulated mycorrhized DEGs and 1247 upregulated and 1398 downregulated nodulated DEGs were identified [47]. Herein, we validated the RNA-Seq results. Five genes with specific expression patterns under corresponding symbiotic condition were selected for RT-qPCR analysis, and then these results were compared with the previously published transcriptomic data [47] obtained through RNA-Seq. First, the wild-type *P. vulgaris* plants were inoculated with *R. irregularis* or *R. tropici,* and total RNA was isolated 2 weeks postinoculation. Later, the transcript abundance of *CRK3*, *CRK12*, *PvPT4* (*P. vulgaris phosphate transporter 4*), *EnodL12* (*early nodulin-like 12*) and transcription factor *Myb73* was measured by RT-qPCR analysis. The results obtained from both RT-qPCR and RNA-Seq analyses were found to be same (Figure 5B,C). *CRK3* expression was specific in mycorrhized roots whereas, *CRK12* expression was restricted to nodulated roots. Mycorrhizal symbiosis-specific *PvPT4* expression levels were significantly induced only in mycorrhized roots; similarly, nodule specific *EnodL12* expression was specific in nodulated roots. The *Myb73* transcription factor characterized as a fungal pathogen *Bipolaris oryzae* resistant gene [51] was downregulated in mycorrhized roots but upregulated in nodulated roots (Figure 5B). Hence, the RNA-seq data was used for the identification of *CRK* family gene expression during mycorrhizal or rhizobial colonization in this study.

### 3.9. CRK Gene Expression Patterns in Mycorrhized and Nodulated Roots

**Figure 6:**
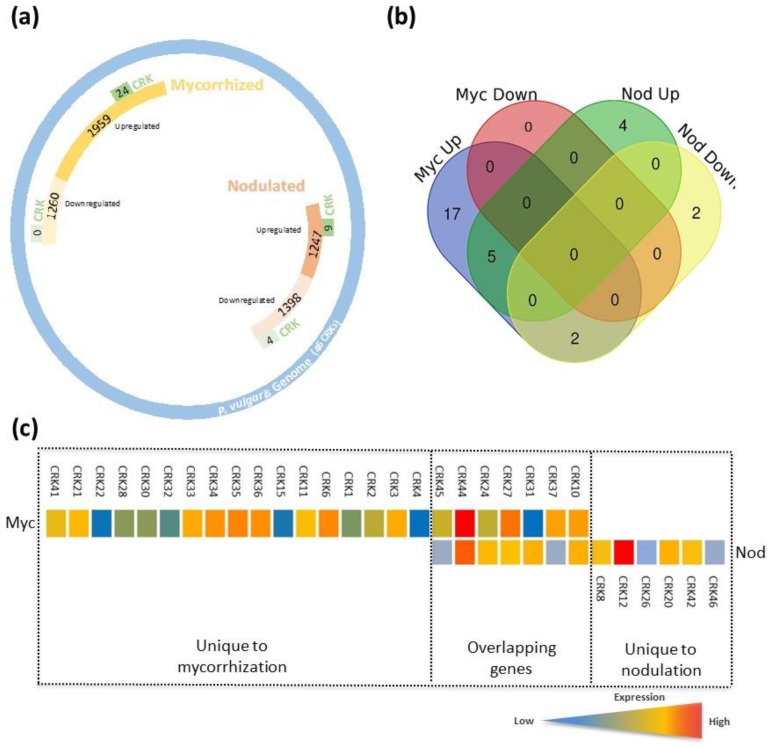
Consolidated representation of genome-wide expression profiling of differentially expressed genes (DEGs) and CRKs in response to root symbionts in *P. vulgaris*. Expression pattern of DEGs in response to mycorriza or rhizobia in *P. vulgaris* roots tissues were obtained based on *p*-values of ≤0.05 and fold changes of ≥2.0 (upregulated and downregulated). (**a**) Global transcriptome profile of mycorrhizal fungi and rhizobia activated and repressed genes, and the number of upregulated and downregulated CRKs under each symbiotic condition. (**b**) Venn diagram showing the number of overlapping expression (upregulated and downregulated) of *CRK* genes in mycorrhized and nodulated roots (clustered into four comparison groups represented by four rounded rectangles) (http://bioinformatics.psb.ugent.be/webtools/Venn/). (**c**) Heat maps showing the unique and overlapping *CRK* gene expression patterns specific to AM and rhizobial colonization. Colour bar shows the fold-change range, with red and blue representing upregulation and downregulation, respectively.

During mycorrizal and rhizobial symbiosis, the host plant recruits specific RLKs to perceive symbiotic signals. CRKs are conserved RLKs across plants and are involved in several key cellular functions. Thus far, the role(s) of CRK family members either in mycorrhizal or rhizobial symbiosis is poorly understood. Herein, based on *P. vulgaris* transcriptomic data [46] we identified differentially expressed CRKs (genes with *p*-values of ≤0.05 and fold-change of ≥2.0 (upregulated and downregulated) were selected). Out of 46 *CRK* family members, we observed 24 and 13 differentially responding *CRK* genes in mycorrhized and nodulated roots, respectively (Figure 6A), indicating that several *CRK* members respond to mycorrhizal colonization compared to nodulation. The Venn diagram intersection and pie chart revealed 17 unique (65%) *CRK* genes (all upregulated) under mycorrhized conditions and 6 unique (16%) *CRK* genes under nodulated conditions (4 *CRK* genes upregulated and 2 downregulated). Seven overlapping (19%) *CRK* genes were found in both the mycorrhizae and rhizobia colonized roots (Figure 6B, Figures S6 and S7). Among the overlapping genes, *CRK37* and *CRK45* were downregulated, whereas *CRK10, CRK24, CRK27, CRK31,* and *CRK44* were upregulated under nodulated conditions. In contrast, all 7 *CRK* genes were upregulated in mycorrhized roots; interestingly *CRK44* was upregulated 7.8-fold compared to 4.9-fold in nodulated roots (Figure 6C). Within the mycorrhized unique genes, *CRK6, CRK34, CRK35, and CRK36* gene transcripts were upregulated over 4-fold and *CRK3, CRK11, CRK33, CRK21,* and *CRK41* were upregulated over 3-fold in mycorrhized roots compared to controls. Among the nodulated unique genes, *CRK12* was upregulated 7.3-fold compared to control roots (Figure 6C).

Next, we compared phylogenetic groups with *PvCRK* genes that respond to mycorrhizal and rhizobial colonization. Our observations show that 7, 6, 11, 3, and 3 *CRK* genes were found in Group I, II, III, IV, and V, respectively (Figure 1A and Figure 6C). These combined results indicate that different members of *CRK* family genes are elicited in *P. vulgaris* roots during mycorrhizal and rhizobial symbioses, and the majority belong to the phylogenetic group III.

## 4. Discussion

Receptor-like kinases are the primary signaling molecules that regulate numerous biological processes, including growth, development and immune responses of plants. CRKs are one of the largest families of RLKs, which have been implicated in abiotic stress, plant defense responses and programmed cell death [11,15,17,52,53,54]. CRKs have been identified and functionally analyzed in *Arabidopsis* [55,56] rice [6], wheat [57], and tomato [51] to decipher their role in various biological processes. Thus far, legume *SymCRK* has been shown to be involved in *M. truncatula* root nodule symbiosis. Gene characterization studies and functional analysis of *CRK* family genes are needed to elucidate the role of *CRK* family genes. Legume crops are a special class of plants with the unique ability to establish symbiotic association with *Rhizobium* bacteria to fix atmospheric nitrogen and arbuscular mycorrhizal fungi to uptake soil nutrients. Herein, as a first step towards understanding the putative role of CRKs during legume symbiotic associations, we conducted genome-wide identification and expression profiling of the *CRK* gene family in *P. vulgaris*.

In this manuscript, we identified 46 *CRK* members in *Phaseolus*. As in rice and *Arabidopsis*, *CRK* genes were clustered together in the genome of *Phaseolus*. The maximum number of genes in a cluster was on chr07, with 22 CRKs, indicating gene duplications. Gene clusters generally facilitate the recombination and accelerated evolution of the associated traits. Phylogenetic analysis of the CRK members resulted in five groups that were based on gene loci, rather than the number of *cys* residues as in case of *Arabidopsis* CRKs. In the present study, we also identified CRK family members in *Z. mays* (22 CRKs) and legumes such as *M. truncatula* (47 CRKs), *G. max* (63 CRKs) and *L. japonicus* (18 CRKs) for the first time.

The conserved nature of gene structures in the number of introns–exons and motif compositions further indicated fewer insertions and deletions during evolution and supported the theory of gene duplication. While analyzing the gene structures, the exon numbers were similar on intrachromosomal genes rather than on interchromosomal genes. Most CRKs had two DUF26 domains, except for CRK9 and CRK26, which had four DUF26 domains. Evolutionary patterns are attributed to three mechanisms of gene duplications, including segmental duplication, tandem duplication and transposition events such as retro position and replicative transposition [58]. Segmental duplications are the most frequent occurrence in plants, as they are diploidized polyploids and retain numerous duplicated chromosomal blocks within their genomes [59]. *CRK* gene family analysis in *Phaseolus* shows their distribution in duplicated blocks, implying segmental duplications.

Theoretical isoelectric point analysis revealed that the majority of PvCRKs have slightly acidic pH, and the remaining proteins have an alkaline pH. Because plants can vary their gene expression in response to the external pH [60,61], the variation in the isoelectric point of PvCRKs could help in the functional diversity of these proteins. The splice variants of the gene families have been shown to have distinct isoelectric points, and the presence of isoforms with varying isoelectric points may help in adoptability to change in external pH [62]. Isoelectric points can also affect protein localization, and hence pI can help in subcellular localization and functionality [63,64]. The analysis of hydrophobicity of CRKs showed negative GRAVY values, which appeared to contradict the hydrophobic nature of membrane localized proteins. Hydropathy analysis showed that PvCRKs possess a transmembrane domain whose secondary structure showed alpha helical structures rich in hydrophobic cysteine residues. These alpha helical structures are highly conserved in all CRK members identified in *Phaseolus*. In the 3D structure, the hydrophobic cysteine residues will be able to bind to a spectrum of hydrophobic molecules to generate varied cellular responses. Transmembrane proteins contain hydrophobic midsections within the membrane and hydrophilic ends, which are exposed to the aqueous cellular and extracellular environment. Hence, we would hypothesize that the negative GRAVY values are the average of both hydrophilic and hydrophobic residues in the CRK members.

*Cis*-regulatory element analysis in promoters of CRK members of *Phaseolus* suggested that they play a role in regulating biotic and abiotic stress responses, and plant growth and development. In the present study, we predicted four types of hormone responsive *cis*-elements in the promoters of *PvCRK* genes, including auxin-responsive, jasmonate-responsive, gibberellin-responsive, ethylene-responsive, and salicylic acid-responsive elements. No motifs related to cytokinin response were detected. In *Arabidopsis*, CRKs are involved in ABA signaling by regulating ABA responses in seed germination, early seedling development and abiotic stress responses. The same *Arabidopsis* study showed plants overexpressing *CRK45* were more sensitive to ABA and hypersensitive to salt and glucose inhibition of seed germination and enhanced drought tolerance, whereas the knockout mutants showed the opposite phenotypes [52,53]. Light responsive *cis*-regulatory elements are another common motif encountered in the promoters of most of the PvCRKs. *CRK5* in *Arabidopsis* is the regulator of UV light responses [7]. Defense response elements were common in PvCRK promoters, which explain their involvement in hypersensitive response, cell death, and disease resistance in *Arabidopsis* [11,12,15]. Although the promoter analysis of PvCRKs did not show symbiosis associated *cis*-elements, the presence of fungal elicitor responsive elements signals the putative role of CRKs during mycorrhizal symbiosis. Nevertheless, previous studies demonstrate phytohormones playing crucial roles in defining the rhizobial and mycorrhizal symbiosis. Auxin regulates nodule development by modulating cell divisions and cell cycle genes in *L. japonicas* and *M. truncatula* [65,66]. The cytokinin receptor CRE1 in *M. truncatula* is known to coordinate nodule organogenesis by integrating bacterial and plant signals [67,68]. Further, abscisic acid is found to coordinate nod factor and cytokinin signaling in *M. truncatula* during nodulation [69]. Although ethylene is an inhibitor of nod factor signal transduction, crosstalk between jasmonic acid and ethylene is essential for the regulation of nodulation [70,71]. Gibberellic acids are also known to play pivotal roles during rhizobia infection and nodule development [72,73,74,75,76,77]. Similar observations were also made during mycorrhizal symbiosis; auxin perception is known to be required for initiation and arbuscule development by influencing the strigolactones [78,79,80]. ABA and ethylene were found to influence the AM initiation, colonization and functionality in *M. truncatula* and tomato [81,82,83,84]. Further, AM symbiosis was reported to be regulated by gibberillins and GA regulating DELLA proteins [79,85,86,87].

*PvCRK* genes show differential expression patterns in various vegetative and reproductive tissues of *Phaseolus*. RT-qPCR analysis of representative *CRK* genes (based on predominant *cis*-regulatory elements for light responsive, defense responsive and hormonal responsive elements) in different *Phaseolus* organs show variable expression patterns and were consistent with the Phytozome transcriptome database. As shown in the results, out of 46 PvCRKs, an average of 50% of genes showed low expression and 22% of genes exhibited high expression in all tissues analyzed. Curiously, CRKs with high expression in most of the tissues belonged to phylogenetic group III, implying their indispensable role in various aspects of growth, development, reproduction and defense in homologues in other plant systems [5,7,11,12,13,14,15,16].

While analyzing the previously reported [47] RNA-Seq data of *Rhizobium*/mycorrhiza inoculated *Phaseolus* for CRK gene expression, interesting facts were uncovered. With the applied cutoff value of the transcripts, 24 *CRK* genes were upregulated and none were specifically downregulated. However, under *Rhizobium* inoculated conditions, 4 *CRK* genes were downregulated and 9 were upregulated. Seven CRKs were shared between the symbiotic conditions. Taken together, 11 CRKs with high expression under mycorrhiza/*Rhizobium* inoculated conditions belonged to group III of the phylogenetic tree of *Phaseolus* CRKs.

## 5. Conclusions

In summary, we performed a genome-wide analysis of the *CRK* family of *P. vulgaris* and identified 46 *PvCRK* genes. An array of the biochemical characteristics of the PvCRK proteins was analyzed. The phylogeny of the PvCRK members classified them into five groups, which were substantiated with the similarities found in the gene structure and motif arrangements. *PvCRK* genes were distributed on 8 chromosomes among 11 chromosomes of *Phaseolus*. Gene clustering was the most common feature of the *CRK* gene distribution, and the largest cluster of 22 genes (*CRK16* to *CRK37*) was found on chr07. Gene clustering indicated the possibility of gene duplication as a factor of *CRK* gene family expansion. *PvCRK* members were differentially expressed in all vegetative and reproductive tissues of *P. vulgaris*. Further, GO analysis revealed divergent roles of CRK proteins in *Phaseolus*. RNA-Seq data for mycorrhiza/*Rhizobium* colonized *Phaseolus* root tissues revealed shared and unique *PvCRK* genes that could play a decisive role during symbiotic events. This article also provides a comprehensive list of CRKs in *L. japonicus*, *M. truncatula* and *G. max* and *Z. mays* as a first report. Taken together, the results provide a foundation for functional characterization of CRK proteins in *Phaseolus* and also other species discussed.

## Figures and Tables

**Table 1:** The *CRK* gene family members in *Phaseolus vulgaris*.

Gene ID *	Gene Name ^$^	*Arabidopsis* Orthologs ^#^	Gene Length, bp	CDS Length, bp	Transcript Length, bp	Protein Length, aa	pI	MW, kDa
**Phvul.002G063900**	*CRK1*	*CRK2*	3373	1950	2342	649	7.54	72.38
**Phvul.002G063700**	*CRK2*	*CRK2*	5195	1950	3126	649	7.19	72.23
**Phvul.002G063600**	*CRK3*	*CRK2*	3272	1632	2114	543	7.54	60.87
**Phvul.002G049500**	*CRK4*	*CRK4, CRK5, CRK6, CRK7, CRK8, CRK10, CRK19, CRK20, CRK23*	3317	2043	2248	680	6.07	74.88
**Phvul.003G062700**	*CRK5*	*CRK3*	4054	1968	2501	655	7.18	72.35
**Phvul.003G062600**	*CRK6*	*-*	3777	1956	2851	651	7.2	72.37
**Phvul.003G202000**	*CRK7*	*-*	3900	1971	2364	656	7.08	71.53
**Phvul.004G011000**	*CRK8*	*CRK42*	5206	2013	2326	670	7.22	73.98
**Phvul.004G125200**	*CRK9*	*-*	3504	2706	2706	901	6.69	102.05
**Phvul.005G015100**	*CRK10*	*-*	4653	1983	1994	660	5.73	74.05
**Phvul.005G014900**	*CRK11*	*-*	3863	1986	2278	661	5.47	74.13
**Phvul.006G006800**	*CRK12*	*-*	2716	1242	1729	413	8.31	47.33
**Phvul.006G084500**	*CRK13*	*-*	3220	1917	1917	638	6.95	70.81
**Phvul.006G084600**	*CRK14*	*-*	3385	1941	2206	646	6.81	71.07
**Phvul.006G084800**	*CRK15*	*-*	4008	1923	1923	640	7.09	70.76
**Phvul.007G052500**	*CRK16*	*CRK28*	3369	1998	2155	665	4.95	74.96
**Phvul.007G051500**	*CRK17*	*-*	4473	2004	2004	667	6.78	75.05
**Phvul.007G051300**	*CRK18*	*-*	4465	2010	2117	669	5.98	75.6
**Phvul.007G051200**	*CRK19*	*-*	4004	1992	2201	663	6.29	74.91
**Phvul.007G051100**	*CRK20*	*-*	4031	1944	1970	647	7.43	73.46
**Phvul.007G051000**	*CRK21*	*-*	3774	1971	2211	656	5.89	73.86
**Phvul.007G050700**	*CRK22*	*-*	3669	1980	2963	659	6.2	73.28
**Phvul.007G050600**	*CRK23*	*-*	3272	1986	2377	661	5.84	73.43
**Phvul.007G050500**	*CRK24*	*-*	4069	2010	2209	669	5.95	74.31
**Phvul.007G050400**	*CRK25*	*-*	3852	1968	2010	655	5.47	72.69
**Phvul.007G050300**	*CRK26*	*-*	8823	3219	3219	1072	5.83	119.67
**Phvul.007G050200**	*CRK27*	*CRK26*	3217	1932	2024	643	6.54	72.12
**Phvul.007G049600**	*CRK28*	*CRK28, CRK29*	3254	2058	2194	685	4.92	77.23
**Phvul.007G049500**	*CRK29*	*CRK28, CRK29*	3188	2010	2186	669	5.63	75.25
**Phvul.007G049400**	*CRK30*	*CRK28, CRK29*	3196	2019	2267	672	5.7	74.77
**Phvul.007G049100**	*CRK31*	*CRK28, CRK29*	3247	2022	2283	673	5.19	75.84
**Phvul.007G049000**	*CRK32*	*CRK28, CRK29*	3126	2058	2199	685	5.24	77.2
**Phvul.007G048900**	*CRK33*	*CRK28, CRK29*	3061	2016	2179	671	5.15	75.69
**Phvul.007G048800**	*CRK34*	*CRK28, CRK29*	3209	2013	2131	670	5.99	75.27
**Phvul.007G048700**	*CRK35*	*CRK26*	3389	2001	2351	666	6.92	74.78
**Phvul.007G048600**	*CRK36*	*CRK28, CRK29*	4596	2052	2307	683	5.13	76.62
**Phvul.007G048500**	*CRK37*	*CRK26*	5186	2034	2264	677	5.12	76.17
**Phvul.008G077800**	*CRK38*	*-*	5648	1995	1995	664	7.01	75.18
**Phvul.008G058700**	*CRK39*	*CRK28, CRK29*	3044	2010	2202	669	5.76	75.13
**Phvul.008G058600**	*CRK40*	*CRK28, CRK29*	8619	2022	2432	673	5.59	74.92
**Phvul.008G156400**	*CRK41*	*CRK42*	3969	1995	2462	664	7.51	73.27
**Phvul.011G193300**	*CRK42*	*-*	2615	1677	2120	558	7.53	63.84
**Phvul.011G194401**	*CRK43*	*-*	3032	2004	2095	667	6.23	75.46
**Phvul.011G194600**	*CRK44*	*-*	8175	1890	1953	629	5.81	70.7
**Phvul.011G194700**	*CRK45*	*-*	5802	1926	1926	641	6.84	72.23
**Phvul.011G196200**	*CRK46*	*-*	3325	2016	2210	671	6.91	7498

* Phytozome gene ID; ^$^ Nomenclature based on *CRK* localization on chromosomes (Figure 1B). bp—base pairs; CDS—coding sequence; aa—amino acids; pI—isoelectric point; MW—molecular weight; kDa—kilodaltons. ^#^ Phytomine—Inparanoid-Orthomcl.

## Supplementary Materials

The following are available online at http://www.mdpi.com/2073-4425/10/1/59/s1, Figure S1: Phylogenetic tree of various *CRK* family genes in legumes and non-legumes. Figure S2: Secondary structure prediction of CRKs in *P. vulgaris* by MLRC. Figure S3: Protein structure of CRKs in *P. vulgaris*. Figure S4. Multiple sequence alignment of PvCRK amino acids using CLUSTAL. Figure S5: Expression patterns of the *CRK* gene in wild-type *P. vulgaris* tissues by RT-qPCR. Figure S6: Hydrophobicity of *P. vulgaris* CRK proteins. Figure S7: Pie chart showing the percent of unique and overlapping *CRK* genes during symbioses in *P. vulgaris*. Table S1: Primer sequences of *P. vulgaris* genes used to perform RT-qPCR analyses. Table S2: The comprehensive list of CRK transcript IDs of different dicots (legumes and non-legume) and monocots. Table S3: The secondary structure of *Phaseolus vulgaris* CRK family proteins. Table S4: List of *Phaseolus* CRK proteins showing GRAVY scores. Table S5: In silico analysis of the *Phaseolus vulgaris* CRK promoter region.
