# Supplementary material for: Cysteine-Rich Receptor-Like Kinase Gene Family Identification in the Phaseolus Genome and Comparative Analysis of Their Expression Profiles Specific to Mycorrhizal and Rhizobial Symbiosis
Source: Genes (Basel). 2019 Jan 17;10(1):59. doi: 10.3390/genes10010059 (PMC6356535; doi:10.3390/genes10010059)
Supplement: Supplementary file 1 [file genes-10-00059-s001.zip › Figure S4.pdf]

Figure S4. Multiple sequence alignment of *PvCRK* genes using CLUSTAL.

```
CRK1  ------------------------------------------------------------
CRK2  ------------------------------------------------------------
CRK3  ------------------------------------------------------------
CRK4  ------------------------------------------------------------
CRK5  ------------------------------------------------------------
CRK6  ------------------------------------------------------------
CRK7  ------------------------------------------------------------
CRK8  ------------------------------------------------------------
CRK9  MASYKILIFFILINFLNFAITEPQDYSPLYQYCSSEQTIDNTSLQVYVEKLLNNLSSLSS 60
CRK10 ------------------------------------------------------------
CRK11 ------------------------------------------------------------
CRK12 ------------------------------------------------------------
CRK13 ------------------------------------------------------------
CRK14 ------------------------------------------------------------
CRK15 ------------------------------------------------------------
CRK16 ------------------------------------------------------------
CRK17 ------------------------------------------------------------
CRK18 ------------------------------------------------------------
CRK19 ------------------------------------------------------------
CRK20 ------------------------------------------------------------
CRK21 ------------------------------------------------------------
CRK22 ------------------------------------------------------------
CRK23 ------------------------------------------------------------
CRK24 ------------------------------------------------------------
CRK25 ------------------------------------------------------------
CRK26 ------------------------------------------------------------
CRK27 ------------------------------------------------------------
CRK28 ------------------------------------------------------------
CRK29 ------------------------------------------------------------
CRK30 ------------------------------------------------------------
CRK31 ------------------------------------------------------------
CRK32 ------------------------------------------------------------
CRK33 ------------------------------------------------------------
CRK34 ------------------------------------------------------------
CRK35 ------------------------------------------------------------
CRK36 ------------------------------------------------------------
CRK37 ------------------------------------------------------------
CRK38 ------------------------------------------------------------
CRK39 ------------------------------------------------------------
CRK40 ------------------------------------------------------------
CRK41 ------------------------------------------------------------
CRK42 ------------------------------------------------------------
CRK43 ------------------------------------------------------------
CRK44 ------------------------------------------------------------
CRK45 ------------------------------------------------------------
CRK46 ------------------------------------------------------------


CRK1  ------------------------------------------------------------
CRK2  ------------------------------------------------------------
CRK3  ------------------------------------------------------------
CRK4  ------------------------------------------------------------
CRK5  ------------------------------------------------------------
CRK6  ------------------------------------------------------------
CRK7  ------------------------------------------------------------
CRK8  ------------------------------------------------------------
CRK9  GHTQYNTNFSCANPSDSIYGLFMCRGDTSPPLCQQCVQSAKNRLSSECSLSKESVTWYEE 120
CRK10 ------------------------------------------------------------
CRK11 ------------------------------------------------------------
CRK12 ------------------------------------------------------------
CRK13 ------------------------------------------------------------
CRK14 ------------------------------------------------------------
CRK15 ------------------------------------------------------------
CRK16 ------------------------------------------------------------
CRK17 ------------------------------------------------------------
CRK18 ------------------------------------------------------------
CRK19 ------------------------------------------------------------
CRK20 ------------------------------------------------------------
CRK21 ------------------------------------------------------------
CRK22 ------------------------------------------------------------
CRK23 ------------------------------------------------------------
```

```
CRK24 ------------------------------------------------------------
CRK25 ------------------------------------------------------------
CRK26 ------------------------------------------------------------
CRK27 ------------------------------------------------------------
CRK28 ------------------------------------------------------------
CRK29 ------------------------------------------------------------
CRK30 ------------------------------------------------------------
CRK31 ------------------------------------------------------------
CRK32 ------------------------------------------------------------
CRK33 ------------------------------------------------------------
CRK34 ------------------------------------------------------------
CRK35 ------------------------------------------------------------
CRK36 ------------------------------------------------------------
CRK37 ------------------------------------------------------------
CRK38 ------------------------------------------------------------
CRK39 ------------------------------------------------------------
CRK40 ------------------------------------------------------------
CRK41 ------------------------------------------------------------
CRK42 ------------------------------------------------------------
CRK43 ------------------------------------------------------------
CRK44 ------------------------------------------------------------
CRK45 ------------------------------------------------------------
CRK46 ------------------------------------------------------------


CRK1  ------------------------------------------------------------
CRK2  ------------------------------------------------------------
CRK3  ------------------------------------------------------------
CRK4  ------------------------------------------------------------
CRK5  ------------------------------------------------------------
CRK6  ------------------------------------------------------------
CRK7  ------------------------------------------------------------
CRK8  ------------------------------------------------------------
CRK9  CMVRYSTNYFFSTETTIQCNTGKVVSNPENFTQLYFETMNKTADEAAHAPVVNNNNKMFS 180
CRK10 ------------------------------------------------------------
CRK11 ------------------------------------------------------------
CRK12 ------------------------------------------------------------
CRK13 ------------------------------------------------------------
CRK14 ------------------------------------------------------------
CRK15 ------------------------------------------------------------
CRK16 ------------------------------------------------------------
CRK17 ------------------------------------------------------------
CRK18 ------------------------------------------------------------
CRK19 ------------------------------------------------------------
CRK20 ------------------------------------------------------------
CRK21 ------------------------------------------------------------
CRK22 ------------------------------------------------------------
CRK23 ------------------------------------------------------------
CRK24 ------------------------------------------------------------
CRK25 ------------------------------------------------------------
CRK26 ------------------------------------------------------------
CRK27 ------------------------------------------------------------
CRK28 ------------------------------------------------------------
CRK29 ------------------------------------------------------------
CRK30 ------------------------------------------------------------
CRK31 ------------------------------------------------------------
CRK32 ------------------------------------------------------------
CRK33 ------------------------------------------------------------
CRK34 ------------------------------------------------------------
CRK35 ------------------------------------------------------------
CRK36 ------------------------------------------------------------
CRK37 ------------------------------------------------------------
CRK38 ------------------------------------------------------------
CRK39 ------------------------------------------------------------
CRK40 ------------------------------------------------------------
CRK41 ------------------------------------------------------------
CRK42 ------------------------------------------------------------
CRK43 ------------------------------------------------------------
CRK44 ------------------------------------------------------------
CRK45 ------------------------------------------------------------
CRK46 ------------------------------------------------------------


CRK1  ------------------------------------------------------MKKV-- 4
```

```
CRK2  ------------------------------------------------------MKKV-- 4
CRK3  ------------------------------------------------------------
CRK4  ------------------------------------------------MRGEREM-VTD- 10
CRK5  ------------------------------------------------------------
CRK6  -----------------------------------------------------MERTA-- 5
CRK7  ------------------------------------------------------------
CRK8  --------------------------------------------MAFKCQSNNYKYKC-- 14
CRK9  TREAPVSGFHTLYCLAQCAPDLSRDDCRTCLGDAIKDIPNSPQCSGVRLGGKVLHPSCN- 239
CRK10 ------------------------------------------------------------
CRK11 -----------------------------------------------------MI-YQWL 6
CRK12 -----------------------------------------------------------M 1
CRK13 ----------------------------------------------------MLQLKL-- 6
CRK14 ----------------------------------------------------MVQLKI-- 6
CRK15 ----------------------------------------------------MFELKL-- 6
CRK16 ------------------------------------------------------------
CRK17 ------------------------------------------------------------
CRK18 ------------------------------------------------------------
CRK19 ------------------------------------------------------------
CRK20 ------------------------------------------------------------
CRK21 --------------------------------------------------------MIT- 3
CRK22 ------------------------------------------------------M-CIL- 4
CRK23 ------------------------------------------------------M-FIL- 4
CRK24 ------------------------------------------------------M-LVS- 4
CRK25 ------------------------------------------------------MFSRNM 6
CRK26 -----------------------------------------------------------M 1
CRK27 ------------------------------------------------------------
CRK28 -------------------------------------------MIIRKASVNVVP-YPD- 15
CRK29 ------------------------------------------------------------
CRK30 ------------------------------------------------------M-AII- 4
CRK31 ------------------------------------------------------------
CRK32 ------------------------------------------------------------
CRK33 ------------------------------------------------------------
CRK34 ------------------------------------------------------------
CRK35 ------------------------------------------------------------
CRK36 ------------------------------------------------------------
CRK37 ------------------------------------------------------------
CRK38 ------------------------------------------------------MPSNN- 5
CRK39 ------------------------------------------------------------
CRK40 ------------------------------------------------------------
CRK41 --------------------------------------------MNT---------KS-- 5
CRK42 -----------------------------------------------------MH-APWF 6
CRK43 ------------------------------------------------------M-VGTQ 5
CRK44 ------------------------------------------------------------
CRK45 ----------------------------------------------------MGM-KTTK 7
CRK46 ------------------------------------------------------M-EGKQ 5


CRK1  ---VYL---VFLCNLSLLIKLLLLSKTV-----IAEPRAKTVLITCGHVR--EHN---TT 48
CRK2  ---VSL---VFLCNLSLLIKLLLLSKTV-----IAEPRDKTVLVTCGHVP--GHN---NT 48
CRK3  ----------MSLASFLLVFLCCGALPA-----LADPRATELAVMCTNTT--AP-MLQRQ 42
CRK4  VSMASL---KLLLLLFILSFLHFN--T-CKAQELNSDPVYL-YHNCSGGN-----TTANS 58
CRK5  -----M---IPSSVSFLLLFLSVFAPLT-----LSDPRAQRAALLCTNRS--VSSLSRRQ 45
CRK6  ---PVV---YFQCFIFLLMKAVLLSKTV-----VAEPRAKTVNITCSQKL--EHN---YT 49
CRK7  ---------------MLLSSSLLIPRAV-----CSGTGIRETGFTCGGSE--NPG---S- 34
CRK8  ---KKS---SFEVVIITVATLLCFSHSA-----LSDPRISEVGFFCGTQK--ASP---SG 58
CRK9  IRYESY-PFYRVPPTNPSPKLVLESKTSRHADLTFSKDPFYVSHNCSTN----ETLTSNN 294
CRK10 MATRIPRFVGFF-------MT--LFTMA-----SAQSLNYV-NDDCHNST--TKEQAITL 43
CRK11 FIIKLVY------------LV--CFVAG--QNSSNSQPLDY-RYACLDQS--S--VPPSA 45
CRK12 VSVIRLKLISIL-MFYLLGTLNVL--P------TETASTFR-HYDCTNK----AKFSPKS 47
CRK13 ---LV-------LTLIWWSYNNFHAYGA-----VENSQTNLLNKGCSAYN--ASN---LR 46
CRK14 ---QEL---TLLVLCSWWSL-----EGT-----VGDPQIFFLKWDCSGFT--APN---LS 45
CRK15 ---IV-------LTMMLWSN--IVEHGA-----TGDPQTRMINSGCSTYN--ATN---LQ 44
CRK16 --MGAV---SCMLL-FFLCGV-------L-SQA---NAQYL-FQSCDN--S-NGNYTANS 39
CRK17 --MASF---NFFILHLTFFSLFLHCSPSPITQG-ALDGASKAYYNCTRN---STFASYSV 51
CRK18 --MAWY-LKYVIPLIFSIITL--NFTATRA------QGLSYQYQVCSTN-----KFTPNS 44
CRK19 --MASC-FTYAIPFIFF-ISL--CFTPTRA------QGLQYQYQVCSPN-----KFTPNT 43
CRK20 ---------MSLLVYVFFLGFFFNFSITEA------TEIYYLNHSCSSN----KTFTPNT 41
CRK21 KS-------KNNLKLFLFCTLVLT--V-----TETSASVFN-NVSCTSN----HTFTPNS 44
CRK22 KSCSTQ---LLIL--SLFVFLNFA---------SEAA-TYS-SHVCT-D---GSFYRPNT 44
CRK23 KSCSTH---LLIL--SLFVFLNFA---------SEAAPTYS-RHACTND---GSFYRPNT 46
CRK24 NFCATS---LTLQSLCLLCMLFHA---------TASPPVYS-AHFCSDQNSDQNSYASDT 51
CRK25 FLSISNNLVTFLLFLSLFGIE---------PFCSSGAPVY---NYCPSNA---SYNSS-V 50
CRK26 IIFNSNCFISFLL-LSLLGSL---------TPLTSATPKYN-DIYCPKNA---SYQSNTT 47
CRK27 -----------MPI-FLFIFLQLI--S-I-SQC-NAQPVFH-LHQCM--NH-KGNYTANS 39
```

```
CRK28 YGMGAV---SCMLL-FFLCCL-------I-SQA---NAQYL-FQSCDN--S-NGNYTANS 56
CRK29 ---MAV---SFTLI-LFLSFSFL-----I-SLG---SA--Q-KYICDN--R-SGNYTANS 38
CRK30 IPMAAI---SCILS-LLL----SS--L-I-SQV---SAQFN-TEYCDN--N-KGNYTINS 45
CRK31 --MAAV---SYMLI-LFLSFPFLK--F-I-SLG---SA--Q-KILCDD--G-HGNYTANS 41
CRK32 --MAAL---SFMLI-LFFSFPFL-----V-SLG---SA--Q-KYVCDK----GGNYTANS 38
CRK33 --MAAL---SFMLI-LFLSFTFLK--F-I-SLG---SA--Q-NVICDD--G-HGNYTANS 41
CRK34 --MASV---SFTLF-SFLRCLCVI--V-I-LA-----QSSA-QTPCDN--T-KGNYTINS 41
CRK35 ----------MTAI-FFLFCLCVT--I-I-ISQ-------G-TAFCDN--S-KGNYTVNS 34
CRK36 --MPLL---LPRLL-FFLWCLPIT--F-FISQVSAVNNTQNFHYFCDDTND-QGNYTTNS 50
CRK37 --MPLL---LPRLL-FFLWCLPIT--F-FISQVSAVNNTQNFHYFCDDTND-QGNYTTNS 50
CRK38 LIFRIFCCILVIT--------FFNFPTTKA---QNEDRVYFEHQNCSAN----I--SLPS 48
CRK39 MAMATI---SSTLL-FFLFWLHAI--M-T-ACETTFGPDFGTSKFCD---S-SMNYTANS 48
CRK40 MAMATI---SSTLL-FFLFWLHVI--M-T-ACEDSFGPDFGASKICI---S-SMNYTANS 48
CRK41 ---ITS---NPP-WILFTTLFLSSFSLS-----LSDPRITEAGLYCGTTK--APL---KA 48
CRK42 LTVRSM-CFGFLL---CLSFS--CLSYT-----QAQTPIYQ-GSYCQNST--Q--ESLTT 50
CRK43 GNVRSI-CFSFLL---LLSFR--SFST------KTKADTYL-GSNCDNTT--Q--QTLSS 48
CRK44 -MIPIRVSVSFL-------FL--LFTTT-----RAQAPIYM-YNFCENST--S----VSP 38
CRK45 MMIPIRVVLSFM-------LL--LFITA-----SAQVPIYM-NNFCDNST--S----VSP 46
CRK46 LNVRSIYSFIFLF---LLSFK--PLVT------EAQSPNYV-GDDCNSTT--T--TSLSS 49
                                                   *

CRK1  IFVPNFVATMDKISDEMRKT---GFGTAVVG---T--GPDTNYGLAQCYGDLSLLDCVLC 100
CRK2  IFVPNFIATMDKISDEMRKT---GFGTAVVG---T--GPDTNYGLAQCYGDLSLLDCVLC 100
CRK3  AFLTNFYDAMEALTDLITSQ---RYALVVKG---TT-QNETVYAFGECMKDLSKPDCDVC 95
CRK4  AYQLNLGTLLTSLSSNAST----EFSNNTVG----TSSSDRVYGLFMCRGDVPSTLCHQC 110
CRK5  VFISNFLEAMDALTPLTTTR---QRGAVVKG---S--GNVTVYAMGECMKDLSQSDCNVC 97
CRK6  IFVPNFVATMEKISEQMSST---GYGTAVEG---TG-GPDTNYGLAQCYGDLSPLDCVLC 102
CRK7  -SASRFMALMDTLSFQVKER---GWGAQT-----LLGSGPPMYALGQCRRDLRSTDCYKC 85
CRK8  NYFPIFTKEMEKLHELVKKN---NWGTHSEG---LS-SATPIYALVQCFQDLSNLDCLQC 111
CRK9  TLQISLTTLFSYLSSKATN--KRYY---------KADVNSTVYGLFMCRGDVPNDVCEKC 343
CRK10 AFRTNLNSTLSRLSSDAAT--SKGYNHTTTGNGSTPVDDDAVYGLYDCRGDVTGPFCQFC 101
CRK11 AYKTNINNLFTSLSSDSAT--SNGFGN-----GTSGIDQDMVYGLYLCRGDVNTSLCHSC 98
CRK12 SYQFNLNKLLSKLTSKINI--T-NYYNTTVS---GTNKRDIVYGLFMCNGYM--EKCGEC 99
CRK13 SFFANMNETFESLRAQISNDKNRHFAVED-----KARGEVLTYAMFQCRNYLSKTECLAC 101
CRK14 NFIQNLNASLDDLTAQVSNQ-SKHFATAK-----ATSGTDPVYAMFQCRNYLSITDCATC 99
CRK15 SFHANINATFSELRGDIIND-SKHFGTTQ-----QATGDVLTFVMFQCRNYLSNNDCLSC 98
CRK16 TYNTNLITLLLSNLSSNTEI--NYGFYNFSH-----GQNADKVNAIGLCRGDVEPNECRSC 92
CRK17 AYRSNVKTLLDWLSSNVTN--NARFYNTTVA---SRHAADTAYGSFLCT--INPEICQRC 104
CRK18 TFQRDLTTLFSSLSSTASS--NVEFFNNTVTA--GTNSSDTVYGLFLCRGDIPSDLCNHC 100
CRK19 TFQSHLTTLFSSLSSKASN--NVQFFNNTVT---GTNPSDTVYGLFMCRGDIPSDLCNHC 98
CRK20 AYETNLRTLLTSLSSHATT--AQFF-N-TTTG--GGDGSEIIYASFMCRGDVSNRTCQEC 95
CRK21 TFNANLNTLLSYLSSNVTN--DTRFFNT-TS----GEDSDTVYGLYMCRGDVPFALCREC 97
CRK22 TFQNNLNLLLSSLVSNATL--HDGFYRTKIS----LGAPGEIKGLFLCRGDVTPSVCHDC 98
CRK23 TFQTNLNILLSSLVSNATL--HDGYYLTNIS----LGAPGEVKGLFLCRGDVTPSVCHHC 100
CRK24 KFMSNLNTLLSSLVSNSSLPSNNGFLRTSVA----KGTPDDIDGRFLCRGDVNETVCHSC 107
CRK25 TFETNLRVLLESLVSNISQ--SDGSYSSAM----GLGTASVASGYFLCRGDVSLTTCNDC 104
CRK26 TFQTNLNVLFASLTSNATQ--GVVSYTTTM----GFGSTNAVNGLFLCRADLFSATSNDC 101
CRK27 TYHNNLNTLLSNLSSNTDI--DYGFYNFSY-----GQESDRVNAIGLCRGDAKPDACRMC 92
CRK28 TYNTNLITLLSTLSSNTEI--NYGFYNFSH-----GQNADKVNAIGLCRGDVEPNECRSY 109
CRK29 IYSTNLNTLLSTLSSNTQI--HYGFYNFSY-----GQNTDTVYAIGLCKGDVEPEECRSC 91
CRK30 TYHNNLNTLLSTLSSHTQI--NYGFYNFSH-----GQNNDKVNAIGLCRGDVKPDECRRC 98
CRK31 TYSTNLNTLLSTLSSNTQI--EYGFYNFSY-----GQNTDTVYAIGLCRGDQQPEECRSC 94
CRK32 TYSTNLNTLLSTLSSNTQI--EYGFYNFSY-----GQNTDTVYAIGLCRGDQQPEQCRSC 91
CRK33 TYSTNLNTLLSTLSSNTQI--EYGFYNFSY-----GQNTDTVYAIGLCRGDQQPEECRSC 94
CRK34 TYHNNLNTLLSSFSSYTQI--NYGFYNFSY-----GQGEDKVYAIGLCRGDLKPDECLKS 94
CRK35 TYYNNLNTLLSSFSTHDQI--NYGFYNFSH-----GQGTDKAYAIGVCRGDRTRDQCLKC 87
CRK36 TYHTNLNTLLSTLISNTEI--DYGFYNFTN-----GESTDKVYAIGLCRGDIKPNECRRC 103
CRK37 TYHTNLNTLLSTLISNTEI--DYGFYNFTN-----GENTDKVYAIGLCRGDIKPDECRRC 103
CRK38 TYQNNLITLFSSFTSNASA--KLFY-NTTIL-----GGNSSVYGMFMCRGDIPLRLCKQC 100
CRK39 TFSTNLHTLLSTLTSHKEI--NYGFYNFSH-----GQNSDKVYAIGLCRGDLKVDECRSC 101
CRK40 TFNTNLHTLLSTLTSHTEI--NYGFYNFSH-----GQNSDKVYAIGLCRGDLKVDECRSC 101
CRK41 NYIPSFTKEMESISQLVTNH---NWGTHVVN---ISGSSIPIYGFAQCFKDLSNTDCLLC 102
CRK42 AYQTNLERILSWMSSDAAT--SKGYNYTSIGNS------STVYGLYNCRGNVVGYFCQFC 102
CRK43 AYQTNLDRILTWMPSDAAT--SNGYNQTSIGTN------SSVYGLYDCGGDAVGYFCQFC 100
CRK44 SYKANVDTLLSWVTTDSDQ--SDGYNYTTVNNN---NDDDAVYGLYSCRYDITGYFCRFC 93
CRK45 SYKANVDTFLSWVTTDSYK--GDGYNYTTVNNNNHNNDDDAVYGLYSCRYDITGYFCQFC 104
CRK46 AYKANLNSVLSWLSSDAAT--SKGYNHNSFGNN-TPGADAAVYGLYDCRGDVSGYFCQFC 106
           .   :  .                                  *        .

CRK1  YAEARTVLP---QCYPYN----SGRIFL--DGCFMRAENYSFFNEYT-G--PG-DKAVCG 147
CRK2  YAEARTVLP---QCYPYN----SGRIFL--DGCFMRAENYSFFNEYT-G--PG-DKAVCG 147
CRK3  FAQCKTRVH---RCSPFQRGTNGGMFFF--DGCYLRYDGYNFFNESL-S--PR-DRTICG 146
CRK4  VLNATRRLR--SDCSLAK----QAVIWY--DECTVRYSNISFFSTVATR--PRVGLLNTG 160
CRK5  LAQCKTQLL---ACLPFQKGTQGGRLFF--DGCYLRYDDYNFFAESF-G--DQ-DTTVCG 148
```

```
CRK6  YAEARTVLP---QCFPHN----GGRIYL--DGCFMRAENYSFYDEYT-G--PG-DKAVCG 149
CRK7  FSQARQVLS---RCVPKT----AGTIYL--DGCFLRYDNYSFIRESV-D--PTRDIGICM 133
CRK8  FATSRTKLP---RCLPSV----SAHIYL--DGCFLRYDNYSFYSEDT-D--PLRDTVNCT 159
CRK9  VQNAAHRIA--LDCNSSQ----EAIVWY--SQCMLRYSFRKIFRTVERS-----PVFSDL 390
CRK10 VSTAASEIL--QQCPNRS----SAVIWY--NYCILRYSNNNFFGNLTTT--PSWEIVEAK 151
CRK11 VQNSSILVM--QHCPNNA----SAILWY--PFCLVRYSNQNFFGNLTIR--PRIPMFDAT 148
CRK12 VSTSIQTLK--SKCVFNK----EAIIWT--HECLVRYSDKFFPNTPEKW--PSRCVRKSE 149
CRK13 FNTATSQIR---NCSA----ANGARIIY--DSCFLRYESDRFYDETN-E--PG-GGVSCG 148
CRK14 LATAAAKIR---NCST---GVNGARVIY--DGCFLRYESNDFFDQIM-P---R-SSILCG 146
CRK15 FNTASTQIR---NCSA----ANGARVIY--DGCFLRYESERFFDQTN-E--VG-NGVSCG 145
CRK16 LKDAGGNIT--QLCPNQK----EATIYY--DNCLLRYSNHSIFGVLDIS--PDFYMANPN 142
CRK17 VKEAAKLLS--SLCTIAK----EAIIWY--EVCYVRYSDRRFFSTVEVS--PKLSFMNDQ 154
CRK18 VGNATQRLSTHADCTLSK----AAVVYY--DECIVRYSNRSFFSTLT--MQAGYALWNPT 152
CRK19 VGNATQRLSTHVDCSFSI----EAVVYY--DECIVRYSNLSFFGTADMEINSGYVLASPI 152
CRK20 VRTAAEQIS--RVCPISK----EALIWY--HECLVRYSNRCFFSTLEEWPRFNFMGSHVT 147
CRK21 VGFATQTIA--SSCPTSK----EAVIWY--NECLLRYSYRFFFSKMEEW--PRHQVNIPL 147
CRK22 VTAAAKNIT--QRCTNQT----ESIIWY--DECMLRYSNSSVL-N-NIV--PGVNLLNVE 146
CRK23 VAAAADNIT--RLCTNQT----ESVIWY--DECMVRYSNLSFL-N-NIV--PSIDYSSEQ 148
CRK24 IAAAAANIT--RLCPNNT----ESYIWY--DECTLIYSNSTFQYD-NMV--PKFTLNDEG 156
CRK25 ITTAAAEIT--QLCPNKT----ESIIWY--DECTLRFTNRYFSPA-ATE--PGASLSNNK 153
CRK26 VATAVTEIR--RRCPNQT----ESIIWY--DECMVRYTDRYFSPT-SVV--PRANLWDDK 150
CRK27 LSDSKVLLT--QLCPNQK----EAILWY--DYCMLRYSNRSIFKTMEAT--PGFS-KRTY 141
CRK28 LKDAGGNIT--QLCPNQK----EASIYN--DHCMLRYSNRSIFGVLDIS--PDFSAYNTA 159
CRK29 LNNSRIYLS--HRCPNQK----KAIMWS--GDCMLRYSNDTIFLKMETS--PTYYWWNVG 141
CRK30 LNDSALTIT--QLCPNQK----EALLWLNTSKCLLRYSHRTIFGVMESS--PGFYLTNVN 150
CRK31 LNNSRVDLT--ERCPNQK----KAIMWS--DQCMLRYSNYTIFHQMETS--PRYYMWNTA 144
CRK32 LNNSRVDLT--QRCPNQK----KAIMWS--SECMLRYSNDTIFNQKETF--PRYYWWNTA 141
CRK33 LNNSRVDIT--ERCPNQK----KAILWS--DQCMLRYSNDTIFNQMEIS--PTYYMWNQP 144
CRK34 LNDSRVSLA--ERCPNQK----EAIWWT--VECMLRYTNRSIFGVMENQ--PTNNNYYNL 144
CRK35 LNDSRAALS--KECPNQK----EAIDWG--GECSLRYSNRSIFGLMENQ--PTLELLYTL 137
CRK36 LNGSRANLT--ELCPNRK----EVIGWYEDERCMLRYSDRSIFGLMETG-KPAYFAWNLG 156
CRK37 LNDSRANLT--ELCPNRK----EVIGWYEDEKCMLRYSNRSIFGLMETG-KPAYFVWNSD 156
CRK38 VGNATQKLSTDPECNQSI----EGFMWY--AECKLRYSNVSFFSIVAT--SPEYALFNNN 152
CRK39 INSSQANLR--QLCPNQK----EAIKWE--EKCMLRYSNRPIFHTMETS--PPYYTNNDE 151
CRK40 LNSSQANLR--QLCPNQK----EAISWE--EKCMLRYSNRPIFHTMDTS--PPYYMHNTY 151
CRK41 YAASRTKLP---RCLPSV----SARIYL--DGCFLRYDNYSFYAEVS-D--PLRDTLNCT 150
CRK42 VSNAAREVR--QHCPNRV----SATVMY--DFCILRYSNENFFGKILTY--PTWHAVGTK 152
CRK43 ISTAVREAP--QLCSNRV----SAVVWN--DYCVIRYSNEDFFGKALTD--PTWHTFGTK 150
CRK44 ITTAASELS--RRCPNAV----RAIIWY--DICIIRYSNQSFNGKVSLT--PTWNITGPK 143
CRK45 ITTASKELS--QRCPNTV----RGIIWY--DVCIIRYSNQNFIGKVSLT--PIWNTTGTR 154
CRK46 VSTASREVL--QRCSNRV----SAIIWY--DFCILRYSNENFFGNVTVD--PSWNHSGPK 156
         .         *                  * :      .

CRK1  N-MTKKNS------------SFQAAAKEAVSRAVQ-DAP----NNKGYARGNVAVEGATN 189
CRK2  N-MTKKNS------------SFQAAAKQAVSRAVQ-DAP----NNKGYARGNVAVEGATN 189
CRK3  T-EDFRG----------NWSVFKANTVELVRNLSIE-TP----KNEGFSAGYVR---RSN 187
CRK4  N-IS-------------NPEAFMTLLFSTINTTAD-EAANFHMGVKKYATNQA--NFSGF 203
CRK5  T-NVSSKNSNNISDGAHSSGIYKANALELVRNLSEV-AP----KNDGFWVGSVE---KKN 199
CRK6  N-TTKKNT------------SFQAAAKKAVMTAVQ-TAP----NNKGYARAEVAVEGTAN 191
CRK7  T-PTLGD------DGG-------KRVAEMVSNVTK--EA----AERGFAVDGE------- 166
CRK8  T-EYGGV------AGEAERLVFGNSVGKVVENVVRVAGN----EGRGFAVGEG------- 201
CRK9  N-ITSR-------------NEEQSFFSVTLAKTLDKVAVMAGDSDERFGKYST--KLNDK 434
CRK10 NS-T-------------DPEELQ-KAESYMQSLKREATVE---SNKLYAMGGF--NLSDG 191
CRK11 QNFT-------------SAGEYDSDAQVLMNGLIQ-IGSE---APLMFGTHMF--NINGT 189
CRK12 D--YEG-----------ELDSFNKVLSSLMDDLLTEA-NEAPMDSIKFAVKKI--TISED 193
CRK13 N-TSS-TVT-----------GFRVAVQQV-LMELQEATP----KIKGFYAATKT-PVVGG 189
CRK14 N-QTANESP-----------AFSAAGQQV-IKDLQRATP----KITAFYAATKT-QAVDG 188
CRK15 N-ITS-NAT-----------GFISVGQQV-IWDIQRATP----KTKRFYAATKT-EVAGG 186
CRK16 N-AT-------------EVVEFNRVLDDLLKELKGKAASG--DSRRKYATDND--TTMNF 184
CRK17 DYV--G-----------DVGRFNNIVWDMLNDLRSEAASASNKS----ADKSV--NITDN 195
CRK18 N-MTNQ-------------ESFKRLLYDTMNKTADD-AAHFPTGAKKFATRET--SIDIF 195
CRK19 N-MTNQ-------------ESFKRLVYVSLNETADE-AVGSGSGGEKFATKET--DIDIF 195
CRK20 N-SRNE-----------KGSYGFWLLSKTLSDAVGEAANAGPAGTMKFATKNA--TVFGS 193
CRK21 GDPVVL-----------HSSGFYDALGSIFDELPNQAALALKGSNNPYAVKQE--NASAS 194
CRK22 S-VPDS-----------DYLQFNNILASTLNDAKQ-EAIS---SGKMFSTKEA--NVTSS 188
CRK23 S-VPDS-----------EITSFINFLASTLKGVAQ-DAVNS-PSAKMFATREA--IFTSS 192
CRK24 S-IVNS-----------NHDQFNQLLLSLLKGLEE-KASASSMGEKKFAAGTV--SVTSA 201
CRK25 S-ISAS-----------DLDSFNRTLFGLLDDLVEKTASS--NSARKFATGDR--EFAGS 197
CRK26 N-ISTS-----------DWGSFNQTLSSLLSGLATKAAGS--QSASKFAIGEM--NFTAT 194
CRK27 N-AT-------------DGDSFNEVLRNLLENLTSQASTG--DSLRKFAVANA--SGPAF 183
CRK28 N-AT-------------EADEFNRVLQNLLRELKDKAASG--DSRRKYATDDN--TTSNF 201
CRK29 N-VTE-------------VDQFNEVLGNLMTSLRDIAASG--DSRRKCAGGKN-ISGLNF 184
CRK30 N-VT-------------EADKFNQALSNLMRNLTVVAASG--DSRLKYAADSA--IAANF 192
CRK31 N-AT-------------DATQFNEVLGNLMKSLKDTAASG--DSRRKYATAEN-ATGLNF 187
```

```
CRK32 K-VREV-----------DVDQFNEVLGNLMKSLRDIAASG--DSRRKYAAGKN-TTGLNF 186
CRK33 N-AT-------------DANQFNEVLGNLMKSLIDTAASG--DSRRKYATAEN-ATGLNF 187
CRK34 N-VT-G-----------SVEQFNTALESLMRNLTRIAASG--DSRRKYATASA--YGSDF 187
CRK35 E-VR-G-----------SVEQFNAALQSLMRNLIQTAASG--DSRRKYATGSA--SAPDF 180
CRK36 N-AT-------------QAEEFNKVLRDLLDGMRSRAASG--DSQSKYATANA--TGPDD 198
CRK37 H-AT-------------QAEEFNKVLRDLLDGMRSRAASG--DSQSKYATAKA--TGPDD 198
CRK38 N-VSSN-----------STISFMNFLRNTMNQT----AEAAADSEARFSTKEA--NLSRS 194
CRK39 N-AT-------------KVDEFNKVLGGLLRNLREKAAGG--DSRRKYATDTA--FVDNF 193
CRK40 N-AT-------------DVDEFNKVLGGLLRNLREKAAGG--DSRRKYATDTA--IVANF 193
CRK41 S-QHGTV------VDKTERLELEESVGRVVESVTNRALA----KGGGFAVGEV------- 192
CRK42 NI-Y-------------NKEETK-KGEDFMRGLIRKATVE---TNKLYYMDGF--NLSFT 192
CRK43 NI-S-------------NTTEIQ-KGQDFVRSLIRKATNE---TNQLYYKEGF--NLSAT 190
CRK44 KI-K-------------DSAEAK-KVEDSMESLITKVTID---TKKFWAVDEF--DWVDN 183
CRK45 KI-K-------------DSSEVK-KAEDSMQSLIRKATVE---SKTFWAVEEF--EWIDN 194
CRK46 NV-S-------------SVEEIQ-KGEGFMRSLIRKATVE---TNQLYYMDGF--NLSST 196


CRK1  ---QSVYVLADCWRTLDKRSCKACLENAFFSL--LGCLPWSEGRALNTGCFMRYSDKD-F 243
CRK2  ---QSVYVLADCWRTLDKRSCKACLENAFFSL--LGCLPWSEGRALNTGCFMRYSDTD-F 243
CRK3  ---VTIYGLAQCWKFVNSSSCQNCLAEAITRI--DSCAPKGEVRALNAGCYLRYATHN-F 241
CRK4  ---QTLYCLAQCTPDLSPGDCRNCLSGVIGDL-PWCCQGKQGGRVLYPSCNVRYELYP-F 258
CRK5  ---VSVYGLAQCWEFVNGSACKKCLADAATRI--ASCATQ-EGRALNAGCYLRFSAHK-F 252
CRK6  ---DSAYVLANCWRSLDAKSCRACLENASSSV--LGCLPWSGGRALNTGCFMRYSDSD-F 245
CRK7  ---EGVFALAQCWSTLNKGRCKICLTEAAKKV--QECVPNVEGRGLFTGCILRYSTRK-F 220
CRK8  ---EGVYALAQCWKTVGGKGCRDCLKKAENEI--RGCLPKKEGRALNSGCYLRYSSDK-F 255
CRK9  ---QTLYTLAQCTKDLPTDDCKGCLGNLIGTEIPWSRLGSIGGRVLYPSCNLRFEFHQ-F 490
CRK10 ---EERYGLVQCTRDLTNDECSQCLDAMIQKV-PQCCGTKRSWQVLAPSCLIKYDDFM-F 246
CRK11 ---QRRYGWVQCSRDITPEECRTCLSNMLEDV-ENCCQEKKVWRVFSPSCIVMYETQP-F 244
CRK12 ---QHLFGFAQCTPYLSKENCKKCLRDAMNFL-QTCARGKTGGRVIYPSCIVRYDHYL-F 248
CRK13 ---SAIYAVSQCVETAIKTNCLNCMQVAFNNL--QRCLPNTDGTAYDAGCFMRYSTTS-F 243
CRK14 ----AIYAIAQCAETLSQDNCLDCLSVESSSI--QGCLPNTNARAFDAGCFMRYSETP-F 241
CRK15 ---SAIYAVAQCVETATEDKCLSCMQVGYNNL--QSCLPKTEGRAYDAGCFMRYSTTP-F 240
CRK16 ---QTIYGLLQCSPDLTQTDCNDCLDEAITRI-PTCCDDRRGGRVVGPSCNIRYENYR-F 239
CRK17 ---QKSYGHAWCLPYLTKENCSWCLSEAIADIPTGCCRGKSGGHVIFPSCGVRYELYP-F 251
CRK18 ---QKLYCLAQCTQDLFPGDCRSCLDSLINSDLPGCCAGSQGGRVYYPNCIIRFEIYP-F 251
CRK19 ---QKLYCLVQCTPDLSPRDCRSCLNSLINSDLPRCCAGRQGGRVLYPNCVIRFEIYP-F 251
CRK20 ---QKIHTLVQCTPDLSSEDCSNCLGDIMR-DIPLCCLGRIGGMVLYPSCTLMFGTRH-F 248
CRK21 ---ITVYGLAQCTPDLVAGDCRRCIADAAAEFAVSCCGGSIGASILFPSCIVRYETYP-F 250
CRK22 ---MKIYTLAQCTPDLSTSECNTCFSSAISSF-PNCCDRKQGARSLLPGCNVRYELYP-F 243
CRK23 ---MTLYSLAQCRPDLSAFDCNMCLTGAISSL-G---EGKRGARNLLPSCNVRYELYP-F 244
CRK24 ---QTLYGMAQCEPDATSGSCAACFENAISSI-PSCCNGSGGARLLLPICSIRYQLYP-F 256
CRK25 SSQRTVYSLTECEPTLTSTECEECLQNAISTL-PSCCEGKQGARALLAWCNVRYELFQ-F 255
CRK26 ---SRVYGLAQCASGMTNAQCEACLVNASITL-GTCCQGKQGARALLAWCDIRYDLYQ-F 249
CRK27 ---QTIFGLVQCTPDLSEKHCDECLLKTISEI-PQCCNGSKGAKFFKPSCIIRYEIFP-F 238
CRK28 ---QAIYGLVQCTPDLTQTDCNDCLDEAIKQI-PTCCDEKRGGRVVGPSCNIRYEDYR-F 256
CRK29 ---QTIYGVVQCTPNLSQQECYQCLDVAISEI-PNCCSGKIGGRVLKPSCNIRFEIYS-F 239
CRK30 ---QTVYGLVQCTPDLSETDCNRCLDGAISEI-PSCCGNKMGGRVLRPSCNIRFESAI-F 247
CRK31 ---QTIYGLVQCTPDLSQQECTQCLDGVISEI-PTCCNGKVGGRVLRPSCNIRFETYS-F 242
CRK32 ---QNIYGVVQCTPDLSQQDCYQCLDGAISEI-PNCCNGKIGGRVLKPSCNLRFESNS-F 241
CRK33 ---QTIYGLVQCTPDLSQQRCTQCLDTAISEI-PNCCNGKIGGRVLRPSCNIRFQTNS-F 242
CRK34 ---QTIYGQAQCTPDLSSEDCTKCLDEAISII-PQCCSGKAGGNVVKPSCRIRFDPYS-F 242
CRK35 ---QTIFGYTQCTPDLSSEDCSKCLNEAISKI-PECCNGKAGGNVLKPSCRIRFDPYI-F 235
CRK36 ---KTIFGLAQCTPDLSEQDCNNCLIQSMEEF-GNCCDSKIGARVVRPSCNLRYETSFPF 254
CRK37 ---KTIFGLAQCTPDLSEQDCNNCLIQSMEEF-GNCCDSKIGARVVRPSCNLRYETSLQF 254
CRK38 ---QTLYALAQCTQDLSPQNCRTCLAEGIK-ILPTCCDGKQGARVIFPSCNIWYEMYP-F 249
CRK39 ---QPIHGLMQCTPDLSRQDCGDCLDWSISAI-PTFAKDKVGALVLLPSCNLRFEIYN-F 248
CRK40 ---QPINGLMQCTPDLSRQDCGDCLDWSISTL-PKFAKDKVGALVLLPSCNLRFEIYE-F 248
CRK41 ---EGVYALAQCWNTIGSDGCRECLRKAGKEV--RGCLPKRDGRALNAGCYLRYSTNK-F 246
CRK42 ---QERYGFVQCSRDLTNGGCRECLEAMLAEV-PKFYEQKLGWSIWSGTCMIKYDDYM-F 247
CRK43 ---ESRYGVVQCTRDLTNEVCRQCLEDILAEV-PKCCEQKVAWMVWSGSCLIKYDDYM-F 245
CRK44 ---EKRYGWVQCDRDIPSDECGECLHVLLDIF-PQCCSTNAQWAVFGPSCGMRMDDEK-F 238
CRK45 ---EKRYGWVQCDRVIENDECGECLHATLDIF-PECCSTNVQWAIFGPSCSIRMDDQN-L 249
CRK46 ---QRRYGMVQCSRDLTNEGCSQCLEAMLAKV-PKCCEHKLGWQVSTASCLIKYDDYI-F 251
                 *        *  *:                        * :       :

CRK1  LNKEQETGSSR--------------------------------GN----VAVIVVVVVSS 267
CRK2  LDKEQETGSSR--------------------------------GN----VVVIVVAVVSS 267
CRK3  YNSSNNNPPQE--------------------------------HRGHKNFAIIVAA-SSA 268
CRK4  YRATATPSPSPSPSPPLLPPPTSG---------------NSRGGGISA-GTIVAIV-VPI 302
CRK5  YNNSTDVAISG--------------------------------NHGQRSLAKILAA-TTA 279
CRK6  LNKEEKKGRSG--------------------------------DN----VLVIVITVVSS 269
CRK7  YNDVALPNINN--------------------------------STEERPGVWMIVACVLS 248
CRK8  YNQGGEDGEGD--------------------------------DSSRK-RIIITAVSVLA 282
CRK9  YFDDPSAGPIQ----------------------------SENKGINVR-KIVIVVI-VP- 519
```

```
CRK10 YQITDQTS-SPLPNPAKK-----------------------G-GSIGS-KTLIIIT-VSV 279
CRK11 FSNGTDSAPAPQQDNEAT-----------------------DGNSRRW-WVIVIIA-VAG 279
CRK12 FPLPR------------------------------------GKRKNLG-VIASIIF-FHA 270
CRK13 FADNQTIDIAPFL---------------------------KQGGS-RK--KWGIISGVVG 273
CRK14 FADNQTVDISPFI---------------------------KQGRSSSK--KWVIIGCVVG 272
CRK15 FADNQTIDIAPFL---------------------------KQGDS-SK--KWAIIGGSVG 270
CRK16 YEQTTIIDPE--TPPPSTIN------------------TSPEESSNTT-IIVIAVI-VPA 277
CRK17 RMAHVNSGWVSPPPLPDSPPPF----------------ASSGKRKQKT-LTIIVTV-VPI 293
CRK18 FRSLPTAPTPSPAGLVPPT--------------------NSGENKSRS-RTIILIV-VP- 288
CRK19 FRSHGSAPTPTPPKL------------------------GEKKSSTS-RTIILVV-VP- 283
CRK20 YRDVNAIQESKPSGIKMG-----------------------SPG-S------IFTV-VI- 276
CRK21 YQHSGTSAPTM-----I-----------------------KGSGNIGT-EVIVIVV-VLV 280
CRK22 YNVSSVSTQ-----PEFF-SPSS--------------------GKGRI-SLIVAIV-VPI 275
CRK23 YNVSAESAK-----PESL-SPSS--------------------GKSSI-SIIVAIA-VPI 276
CRK24 LYNST---------AMIL-IPSS--------------------GSKSI-LMVVAIV-IPI 284
CRK25 YNTSATSAPSSG------------------------------SNNNVV-RVLVIVA-LVI 283
CRK26 YNTSIASSPVPSPPPPPSE----------------------KKKSGSQ-RTIIIVV-VAV 285
CRK27 YDPINAATTHASPPPLPQAS--------------------TKGKIIVS-GSVIAIA-VTI 276
CRK28 YEQTTIIDPE--TPPPSTIN------------------TSPEESSNTT-IIVIAII-VPA 294
CRK29 YGNTTTLDP--EAPPPSTNTTS------------SINTTSSQESDNTT-IIVIAVV-VPT 283
CRK30 YDQTPKLDPDVTPPSPPPSSFT---------------NTSPKESNNTI-TIVIAVV-VSI 290
CRK31 YGATTTLDPDPEAPPPSTNT------------------TSSQESDNTT-IIVIAVV-VPT 282
CRK32 YENTTTLDPDPEAPPPSTNTTSSTNNTSSTNNTSSTNTTSSQESDNTT-IIVIAVV-VPT 299
CRK33 YGNPTTLDTDPEAPPPSTNT------------------TSSQESDNTT-IIAIAVV-VPT 282
CRK34 YGPTLNIDLDAPP-SPSTNNT-----------------SSSQGKSNTS-RTIIAIV-VSV 282
CRK35 YGPTLKLDPDAPPPSRLTNNTS----------------LHLQGKSKTS-RTIILIA-VPV 277
CRK36 YGA-PAYTPSPSLSPPS--------------------NTSSEGKSNTI-IIAIATA-VPV 291
CRK37 YGA-AAYTP-------S--------------------NTSSEGKSNTV-IIAIATA-VPA 284
CRK38 YGLVTDNIPPK--TLVPSPGF----------------SSSSSSSSYQT-RTIILIL-ISI 289
CRK39 YNSTVILDPPLPP----ASLP-----------------LHQEDHGNSL-RTAIIIV-VPT 285
CRK40 YNSTAILDPPLPPALPPAPLS-----------------LHQEDHGNSL-RTAIIIV-VPT 289
CRK41 YNEDGDAGGKN--------------------------------GFLRR-GVIIAEV-LAA 272
CRK42 YL------------PETD-----------------------KDGVNKR-SKILIIS-FSV 270
CRK43 YLLNNDTTPAPAANPQTD-----------------------KQGVNNR-SRILIII-FSV 280
CRK44 YQSSG------------------------------------NGGAS-K-TRKLIIS-SSV 259
CRK45 YQNSG------------------------------------NGGISSK-SRKLIIS-FSV 271
CRK46 YLFHNPASSVSVPDTQIA-----------------------KQRSGSK-AKLLIIG-LSV 286

CRK1  VIVSVVGV--TIGVYIWKQRYIQKKRRGSNDAEKLA------------------KSLQNK 307
CRK2  VIVSVVGV--TIGVYIWKQRYIEKKRRGSNDAEKLA------------------KSLQNK 307
CRK3  SLAFLLIV--STVIFLVWTNLLK-RRRERRQVGVLL------------------NTVIKS 307
CRK4  SVAVLLF---IVGICFLSRRARK------KASAKE---------G----KNVD-DITTLE 339
CRK5  ALALLLVV--ATVVFFIRKNMVT-RRRERRQFGALL------------------DQVNKS 318
CRK6  AIVLVVGI--AIVVYIRKHRYVQMKRRGSNDAEKLA------------------KSLHHN 309
CRK7  AIVGILLI--AFAAFICCQKMSS-RRKVRDSGGVSP------------------DFSSVA 287
CRK8  -AAVVVLS--LLVTYVAFTK-----KRKNNNFIEIP------------------SSLKNS 316
CRK9  TIILVNLF--L-LAWYFQRRNTR------KSHTTIL-----------KENFGH-ETATLE 558
CRK10 LVALIFLCCS--IYYLWRKYVTN------KDELI---------SVNTLRSFHG-YVQREE 321
CRK11 TVALALLAIS--IYYCYLKRKKD------KQSIQEE--------GLNSIFSQE-QTDREE 322
CRK12 LVPVIVLF----FVCYLLHRESR------KDLKHRK------------GNCGEEISTELN 308
CRK13 -AVL-LLL--LLIAWRWYG---KSKG--VHRGDILG------------------ATELKG 306
CRK14 -GAGLVAI--LLLLFPWRRRPQSPKR--VAGGNILE------------------ATELKG 309
CRK15 -GFVVLLC--VLFAWLWFT---KPKR--VPRGDILG------------------ATELKG 304
CRK16 A-AV-VLV--TCLFLYLRRRKAR------KNL---AV------KEEEDEKEED-EISIVE 317
CRK17 VVSLVLLS--LGCSCFLRRKETK------NQHDILK------------ESFGN-DSTTLE 332
CRK18 IVSLATFL--T-LLYYVLWRRAR------KSSKILL-----------RKNFGE-ESSTLQ 327
CRK19 IVSLATLS--A-FCYYLLRRRAR------KRLKNLL-----------RENFGE-ESSILE 322
CRK20 LLVLTMLI--SFHCYFLWRRARK------RKYKILL-----------KENFGQ-ESVTLE 316
CRK21 ALLVIFFCFGYYGFIWIKGRKKR------KASDHR---------E----NFGE-EISVLE 320
CRK22 VVAVVLL---IVGVCFLRKRASN------TYLSSF---------R-QDSIVD--DLTDVE 314
CRK23 VVAVVLF---IVGVCFLRKRASN------RYINSF---------R-QDSIVD--DLTDVE 315
CRK24 AILAALL---FIGCCWWRRRQRK------NREDVQ---------EVNDSIRRY-LASEEE 325
CRK25 V-SIILL---CGVCYFIFERSRK------KYNTLL------------RENFGE-ESSTLE 320
CRK26 A-ATILV---FSGGYFLVRRSRR------NYKTLL------------KENFGD-DSATLE 322
CRK27 SAFVILVN--LICCINLRMRSCR------KQFE---------------AENDD-EIQSTK 312
CRK28 A-AV-VLV--ICLFLYLTRRNAR------KNHAGQTI------KEEEDEEDQN-EINIVE 337
CRK29 V-VVVLFL--ICLFLYLRRLKAR------KNLPVIED-------D-----DED-KINIVE 321
CRK30 VVVAVVSL--LGLCIYLRRKKAR------KSPTVNQ------------DDDDD-DIEISQ 329
CRK31 V-VVVLFL--ICLFLYLRRLKAR------KNLPVKEE-------EEDADEDEG-EIKIVE 325
CRK32 V--VVLFL--ICLFLYLRRLKAR------KNLPVIED-------D----EDED-EINIVE 337
CRK33 V-VVVLFL--ISLFLYLRRLKAR------KNLAVKEE-------DD--VEDED-EIKMAE 323
CRK34 A-CVVLVL--TLLCIYLRLRKQR------KKIE----------FEREEESDED-EISFTE 322
CRK35 A-SAILAL--SLFCIYLIVRKPR------KKIEITR---------EKDSDDED-EVTFAE 318
```

```
CRK36 VVIVSAVL--IFIYIRLKARKSG------KKFETKQE------E-LYDDDDDD-GIDASE 335
CRK37 IVIVSAVV--IFIYIRLIARKSW------KKFETKQE------EEELYDDDDD-GIDASE 329
CRK38 IILLGLLF--G-ICYSLMRRKTK------KSNKVIL-----------RENFGL-GSSTIE 328
CRK39 VIVVLVLL--IVISRYLRRK-AR------KNLL------------AEEDGDDD-EIEMVE 323
CRK40 VIVVLVLL--IVIGRYLRRK-AR------QNLL------------AEDDGDDD-EIGMVE 327
CRK41 -AAVLMLI--LSASYVAFTKLTR-IKKENNNLGHIS------------------SSISKS 310
CRK42 MGSITLLCLG--VYFFWNRRR----------------------DGIRLSSFH--KIQTEE 304
CRK43 IGAIIVLCFS--LYCFWYRKRVR------KGNHEEKTRDGVREEELPLPSFH--KIQWEE 330
CRK44 LGSVALLCFS--VYCFWYRKRVR------KDKMT----------------------FDEE 289
CRK45 LGAIALLCFS--VYCFWYRKRVR------KDKMM----------------------LDEE 301
CRK46 LGAVALLCFS--VYCFWFRKRTR------SGRKT----GGRIPDTIRLSSYH--NVQTDE 332


CRK1  SLNFK-----YSTLDKATGSFHENNKLGQGGFGTVYKGVLADGREIAIKRLYFNNRQ-RA 361
CRK2  SLNFK-----YSTLDKATGSFHENNKLGQGGFGTVYRGVLADGREIAIKRLYFNNRH-RA 361
CRK3  KLNMP-----YEILEKATDYFSDSNKVGEGGSGSVFKGVLPDGTTVAVKRLTFNTSQ-WA 361
CRK4  SLQFG-----FGTIEAATNNFSADNKLGEGGFGEVFKGTLPSGQVIAVKRLSKSSGQ-GG 393
CRK5  RLNIS-----YEILEKATDYFNDSNKLGQGGSGSVYKGVMPDGITVAIKRLRFNTTQ-WV 372
CRK6  SLNFK-----YSTLEKATNSFDEDNKLGQGGFGAVYKGVLPDGREIAIKRLYFNNRH-RA 363
CRK7  GFSFR-----YDLLEKATDHFDPANKLGQGEAASVFKGTLPSGGTVAVKRLFFNKRQ-WT 341
CRK8  SLNYK-----YEILEKATDYFSSPRKLGQGGAGSVFKGTLPNGKDVAVKRLVFNNRQ-WV 370
CRK9  PLHFN-----LTAIELATNNFSNDNKIGRGGFGEVYKGILLDGRQIAVKRLSKSSKQ-GV 612
CRK10 PLNVDLPTIPLIWIQQSTNYFSEFSKLGEGGFGSVHKGNLEDGTEVAVKRLSKASGQ-GL 380
CRK11 SMNTDFPMMPLSTILKCTNNFSEEQKLGKGGFGPVYKGTLPDGRQIAVKRLSKTSVQ-GG 381
CRK12 SLQFE-----LRTIQEATNKFSDDNKIGEGGFGAVYKGGFSNGLEIAVKRLKKNSSQ-GA 362
CRK13 PVNYK-----YNDLKAATKEFSAENKLGEGGFGDVYKGTLKNGKVVAVKKLVLGKSSKME 361
CRK14 PKKYK-----YSDLKAATKNFSEKNKLGEGGFGAVYKGTMKNGKVIAVKKLISGKSSKIN 364
CRK15 PVNYK-----YNDLKAATKKFSAENKLGEGGFGDVYKGTLKNGKVVAVKKLVLGKSSKME 359
CRK16 SLQFN-----FDTIRVATEDFSDSNKLGQGGFGAVYWGRLSNGQIIAVKRLSRDSGQ-GD 371
CRK17 SLRFE-----LAKIEAATNRFAKENMIGKGGFGEVYRGILSDGQEIAVKRLTGSSRQ-GA 386
CRK18 GLQFD-----LVTIKEATNNFSPENEIGKGGFGEVYKGILLDGRYIAVKRLSKSSRQ-GS 381
CRK19 GLQFD-----LATIKLATDNFSNEREIGKGGFGEVYKGVLVDGRHIAVKRLSIGSKQ-GS 376
CRK20 GLQFD-----LAIIEAATNNFSNENKIGKGGFGEVYKGILSCGLHIGVKRLSQSSTQ-GS 370
CRK21 SLEFD-----FATIEAATNKFSEERRIGKGGYGEVYKGILPNGEEVAVKRLSTNSKQ-GE 374
CRK22 SLQFD-----MATVEAATNRFSDENKIGRGGFGVVYKGVLSNGQEIAVKRLSVTSLQ-GA 368
CRK23 SLQFD-----LATIEAATNRFSDENKIGQGGFGVVYKGVLPDGQEIAVKRLSVTSLQ-GA 369
CRK24 SLRFD-----LATIEAATNSFSNEMKIGEGGFGAVYKGTFPNGEEIAVKRLSRTSLQ-GD 379
CRK25 SLQFD-----LATMEVATKKFSLENRIGEGGFGDVYKGILPDGREIAVKRLSHSSGQ-GA 374
CRK26 SLQFS-----LATIEVATKKFSYENKIGRGGFGEVYKGILLDGRHIAVKKLSHTSRQ-GV 376
CRK27 TLPLE-----FASIMVATNNFSDANKLGEGGFGPVYKGRLSYGQEVAVKRLSKNSLQ-GN 366
CRK28 SLQFN-----FDTIRVATEDFSDSNKLGQGGFGIVYWGRLSNGQMIAVKRLSRDSGQ-GD 391
CRK29 SLQFN-----FDTIRVATEDFSDSNKLGQGGFGAVYWGRLSNGQMIAVKRLSRNSGQ-GD 375
CRK30 SLQFD-----FDTIRVATEDFSNSNKLGQGGFGAVYRGRLPNGQMIAVKRLSSGSSQ-GD 383
CRK31 SLQFN-----FDTIRVATADFSDSNKLGQGGFGAVYRGKLPNGEIIAVKRLSRNSGQ-GD 379
CRK32 SLQFN-----FDTIRVATEDFSDSNKLGQGGFGAVYWGRLSNGQIIAVKRLSRNSGQ-GD 391
CRK33 SLQFN-----FDTIRVATEDFSDSNKLGQGGFGAVYWGRLPNGQIIAVKRLSRDSGQ-GH 377
CRK34 PLQNN-----FNTIRIATNGFADSNKLGQGGFGAVYKGQLSNGQEIAVKRLSRDSGQ-GD 376
CRK35 SLQFN-----FETIRVATNEFADSNKVGQGGFGAVYRGRLSNGQAIAVKRLSSDSGQ-GD 372
CRK36 SLQIR-----FSIIREATDDFSDSNKLGQGGFGAVYRGTLPNGQEIAVKRLSANSGQ-GD 389
CRK37 SLQIR-----FSIIREATDDFSDSNKLGQGGFGAVYRGTLPDGQEIGVKRLSANSRQ-GN 383
CRK38 SLQFN-----LALIEAATNNFADDNKIGKGGFGEVYKGILIDGTSIAVKRLSRNSKQ-GL 382
CRK39 SLQFN-----LSTIQVATSNFSDSNKLGEGGFGSVYQGKLSNGQVIAVKRLSRNSGQ-GD 377
CRK40 SLQFN-----LNTIQVATSNFSDSNKLGEGGFGSVYQGKLSNGQVIAVKRLSTNSGQ-GD 381
CRK41 SLNYK-----YETLEKATDYFNSSRKLGQGGAGSVFKGILPNKKVIAVKRLIFNNRQ-WV 364
CRK42 TLNTDLPIIPLITVLQSTDNFSEASKLGEGGFGSVYKGILPDGRQIAVKRLSKFSGQ-GS 363
CRK43 TWNTDLPRIPFATILQSTYNFSEASKLGEGGFGPVYKGTLPDGTQIAVKRLSKFSGQ-GS 389
CRK44 KLNGDLPTIPLIAVLHSTNNFSEESILGEGGFGSVYKGILPDGRQIAVKRLSQFSGQ-GS 348
CRK45 KLNGDLPTIPLIAVLHSTNNFSEAFKLGEGGFGPVYKGILPDGRQIAVKRLSKFSGQ-GS 360
CRK46 TLNPDVPIIPLNTILQSTDNFSEASKLGEGGFGPVYKGTLPDGRKIAVKRLSESSGQ-GS 391
                   :  .*  *     :*.*  . *. * :     :.:*:*   .

CRK1  ADFYNEVNIISSLEHKNLVRLLGCSCSGPESLLVYEFLPNKSLDRFIFD-KNKGKELNWE 420
CRK2  ADFYNEVNIISSVEHKNLVRLLGCSCLGPESLLVYEFLPNKSLDRFIFD-KNKGKELNWE 420
CRK3  DHFFNEVNLISGIHHKNLVKLLGCSITGPESLLVYEFVPNHSLYYHLSG-RRNSQQLTWE 420
CRK4  EEFKNEVVVVAKLQHRNLVRLLGFCLQGEEKILVYEYVPNKSLDYTLFD-PEKQSELDWR 452
CRK5  DHFFNEVNLISDIQHKNLVKLLGCSITGPESLLVYEYVPNQSLYDHLSV-RRISQPLSWE 431
CRK6  ADFYNEVNIISSVEHKNLVRLLGCSCSGPESLLIYEFLANRSLDRFIFD-KNKGRELNWT 422
CRK7  EGFFNEVNLINGIQHKNVVKLLGCSIEGPESLLVYEFVPSGSLDQVLFS-KDSEDALNWE 400
CRK8  DDFFNEVNLISGINHKNLVKLLGCSIEGPESLIVYEYLPNKSLDHFLFE-KDKTRILKWK 429
CRK9  AQFKTEVLLIAKLQHRNLVEFIGFCLEEEEKILIYEYVPNKSLDHFLFD-SQPQKLLSWC 671
CRK10 EEFKNEVIFIAKLQHRNLVRLLGCCIEENEKLLVYEYMPNSSLDSHLFN-EEKRKQLDWK 439
CRK11 EEFKNEIILIAKLQHRNLVRLLAGCVEQNEKLLVYEYMPNSSLDFHLFD-TNKGVHLNWI 440
CRK12 IEFKKEVLLISKLQHRNLVRLLGFCVERNEKILIYEFVHNKSLDYYLSSKS*-------- 414
CRK13 DDFEGEVKLISNVHHRNLVRLLGCCSKGQERILVYEYMANSSLDKFLFG-HRKG-CLSWK 419
```

```
CRK14 DDFESEVTLISNVHHRNLVRLLGCCSKNQDRILVYEFMANNSLDKFLFG-KRKG-SLSWK 422
CRK15 DDFEGEVKLISNVHHRNLVRLLGCCSKSQERILVYEYMANSSLDKFLFG-NRKG-SLSWK 417
CRK16 TEFKNEVLLVVKLQHRNLVRLLGFCLEGRERLLVYEFVPNKSLDYFIFD-PSMKAQLDWE 430
CRK17 VEFKNEVQVIAKLQHRNLVRLLGFCLEDDEKILIYEYVKNKSLDYFLSD-IKKRALLSWS 445
CRK18 VEFRNEILLIAKLQHKNLVAFIGFCLEEEEKILIYEYVSNRSLDYFLFG-YP-EEKLSWY 439
CRK19 VEFKNEILLIAKLQHRNLVAFMGFCLEEEEKILIYEYVPNRSLDYFLFG-NQ-QQKLSWH 434
CRK20 IEFKNEVLLIAKLQHRNLVELIGFCLAAQEKILIYKFMHNGSLDKFLFG-NQ-QKTLSWS 428
CRK21 EEFKNEVLLIAKLQHKNLVRLIGFCQEDREKILVYEYVPNKSLDHFLFD-SQKRRVLTWS 433
CRK22 VEFRNEAALVAKLQHRNLVRLLGFCLEGQEKILIYEYIPNKSLDHFLFD-PVKQRELDWS 427
CRK23 VEFRNEAALVAKLQHRNLVRLLGFCLEGQEKILIYEYIPNKSLDYFLFD-SIKQRELDWS 428
CRK24 REFKNEVLLVAQLQHRNLVRLLGFCMEGTEKILIYEFIQNSSLDRFLFG-HEDQGGLDWA 438
CRK25 AEFKNEILLIAKLQHRNLVTLLGFCLGAQEKMLIYEFVSNKSLDYFLFD-PDKSRQMKWS 433
CRK26 LEFKNEILLIAKLQHRNLVTLIGFCLENQEKILIYEYF----FDYIFTD-SHKTRLLSWI 431
CRK27 TEFKNEVLLVAKLQHRNLVKLLGFCLERRERLLVYEFVPNKSLDFFIFD-EDRREQLDWE 425
CRK28 TEFKNEVVLVVKLQHRNLVRLLGFCLEGRERLLIYEFVPNKSLDYFIFD-PAMNAQLDWE 450
CRK29 TEFKNEVVLVVKLQHRNLVRLLGFCLEGRERLLIYEFVPNKSLDYFIFD-PAMKAQLDWG 434
CRK30 TEFKNEVLLMAKLQHRNLVRLLGFCLEGRERLLIYEFVPNKSLDYFIFD-PVKKAQLDWE 442
CRK31 TEFKNEVVLVVKLQHRNLVRLLGFCLEGRERLLVYEFVPNKSLDYFIFD-PAMKAQLDWE 438
CRK32 TEFKNEVVLVVKLQHRNLVRLLGFCLEGRERLLIYEFVPNKSLDYFIFD-PAMKAQLDWG 450
CRK33 TEFKNEVVLVVKLQHRNLVRLLGFCLEGRERLLVYEFVPNKSLDYFIFD-PTMKAQLDWE 436
CRK34 LEFKNEVVLLAKLQHRNLVRLLGFGLEGRERLLVYEFVPNKSLDYLIFD-PTMKPQLNWE 435
CRK35 TEFKNEVLLLAKLQHRNLVRLIGFCLERRERLLIYEFVPNKSLDYFIFD-PIKKTRLDWQ 431
CRK36 TEFKNEVLLLAKLQHRNLVRLLHFCMEGREKLLVYEFVPNKSLDYFIFD-ETKRAQLDWY 448
CRK37 TEFKNEVLLLAKLQHRNLVRLLGFCMEGREKLLVYEFVPNKSLDYFIFD-KTKRAQLDWY 442
CRK38 EEFKNEVLLISKLQHRNLVTFMGFCVNEEEKILIYEYVTHKSLDYFLFD-TKQEKFLTWP 441
CRK39 LEFKNEVLLLAKLQHRNLVRLLGFSLQGREKLLVYEFVPNKSLDYFIFD-PTEKAQLDWD 436
CRK40 LEFKNEVLLLAKLQHRNLVRLLGFSLQGREKLLVYEFVPNKSLDYFIFD-PTEKAQLDWD 440
CRK41 DEFFNEVNLISGIEHKNLVKLLGCSIEGPESLLVYEYLPKKSLDQFIFE-KNRTQILNWK 423
CRK42 EEFNNEVMSIAKLQHRNLVRLFGCCLEENEKILVYEYMPNASLYFHLFD-DEKRKQLDWK 422
CRK43 EEFDNEVMFIAKLRHRNLVRLLACCSEGNEKILVYEYLPNKSLDFHLFD-VEKRKHFDWK 448
CRK44 QEFKNEVMFIAKLQHRNLVRLLGCCLEENEKILVYEYMCNASLDFYLFGDDEKRKQLDWG 408
CRK45 QEFKNEVMFIAKLQHRNLVRLLGCCLEENEKILVYEYMCNSSLDFHLFGDDEKRKQLGWR 420
CRK46 EEFKNEVMFIAKLQHRNLVRLLACCLEEKEKILVYEYMTNASLDFHLFD-ERKKRQLDWK 450
        *  *   :  :.*:*:* ::       : :::*::.    :   :

CRK1  KRFEIIIGTTEGLVYLHENSKTRIIHRDIKASNILLDAKFRAKIADFGLARSFQEDKSH- 479
CRK2  KRFEIIIGTTEGLVYLHENSKTRIIHRDIKASNILLDGKFRAKIADFGLARSFQEDKSH- 479
CRK3  VRHKIILGTAEGLAYLHEES-QRIIHRDIKLGNILIDENFTPKIADFGLARLFPEDKSH- 478
CRK4  RRYKIIQGTARGTQYLHEDSRLRIIHRDLKASNILLDGDMNPKISDFGMARIFGVDQTQ- 511
CRK5  FRHKIILGIAEGLAYLHEESHLRIIHRDIKLSNILLEEDFTPKIADFGLARLFPEDRSH- 490
CRK6  KRYDIIVGTAEGLVYLHENSNIRIIHRDIKASNILLDAKLRAKIADFGLARSFQDDKSH- 481
CRK7  QRFQIICGLAEGLAYLHEGSGTKIIHRDIKSSNILFDENMNPKIADFGLARSVTGNKSH- 459
CRK8  QRFEIVVGIAEGLAYLHGGSEIRIIHRDIKSSNVLLDENLNPKIADFGLARCFGADKTH- 488
CRK9  ERYKIIGGVARGIVYLHEHSRLKVIHRDLKPSNILLDENMIPKISDFGIAKIVEISQDQ- 730
CRK10 LRLSIINGIAKGLLYLHEDSRLRIIHRDLKASNVLLDQEMNPKISDFGLARTFEKGQSQ- 498
CRK11 NRLNIINGIAKGLLYLHEDSRLRVIHRDLKASNILLDHEMNPKISDFGLARTFGGDQKQ- 499
CRK12 ------------------------------------------------------------
CRK13 QRYDIILGTARGLAYLHEEFHVSIIHRDIKTANILLNDDLQPKIADFGLARLLPEDLSH- 478
CRK14 QRYDIILGIARGLTYLHEEFHVSIIHRDIKSGNILLDEELQPKISDFGLVKLLPGDQSH- 481
CRK15 QRYDIILGTARGLAYLHEEFHVSIIHRDIKTSNILLDDDLQPKIADFGLARLLPEDRSH- 476
CRK16 KRYKIIRGIARGLLYLHEDSQLRIIHRDLKASNILLDQEMNPKIADFGMARLVLMDQTQ- 489
CRK17 ERQKIIRGIARGILYLHEDSCLKIIHRDLKPSNVLLDSNMNPKISDFGMARIVAGDEIE- 504
CRK18 QRYNIIKGTALGIFYLHEYSRLKVIHRDLKPSNVLLDENMNPKISDFGLARIVDIDQNR- 498
CRK19 ERYKIIGGTASGILYLHEYSQLKVIHRDLKPSNILLDENMNPKISDFGLARIFDVDQNR- 493
CRK20 QRFKIIEGIAQGILYLHEHSRLKVIHRDIKPSNILLDKNMNPRISDFGMAKIMEIDQDH- 487
CRK21 ERFKIIKGIARGILYLHEDSRLKIIHRDIKPSNVLLDNDINPKISDFGMARMVDTNHIQ- 492
CRK22 RRY-IIVGITRGILYLHEDSQLRIIHRDIKASNVLLDQNMNPKISDFGMAKIFEADQTQ- 485
CRK23 KRYKIIVGIARGILYLHEDSQLRIVHRDLKASNVLLDENMNSKISDFGMAKIFQADQTQ- 487
CRK24 RRYKIIVGIASGIQYLHEDSRLKVIHRDLKASNVLLDSDMNPKISDFGMAKIFHGDQSRE 498
CRK25 QRYKIIEGITQGILYLHEHSRLKVIHRDLKPSNVLLDNNMNPKISDFGMARMVAVDQYE- 492
CRK26 ERYKIIQGIAQGILYLHDHSRLKVIHRDLKSSNVLLDNNMNPKISDFGIARIIALEQDQ- 490
CRK27 KRYKIIIGIARGLLYLHQDSSVRIIHRDLKASNILLDAEMHPKISDFGMARLFEVDQSQ- 484
CRK28 KRYKIIRGIARGLLYLHEDSQLRIIHRDLKASNILLDEEMNPKIADFGMARLVLMDQTQ- 509
CRK29 KRFKIITGIARGLLYLHEDSQLRIIHRDLKAGNILLDEEMNPKIADFGTARLVLMDQTQ- 493
CRK30 MRYKIIRGIARGLLYLHEDSLLRIIHRDLKASNILLDEEMNPKIADFGMARLVLLDETH- 501
CRK31 KRYKIIRGIARGLLYLHEDSQLRIIHRDLKASNILLDQEMNPKIADFGMARLVLMDQTQ- 497
CRK32 KRFKIITGIARGLLYLHEDSQLRIIHRDLKAGNILLDEEMNPKIADFGTARLVLMDQTQ- 509
CRK33 KRYKIITGIARGVLYLHEDSQLRIIHRDLKASNILLDQEMNPKIADFGMARLVLMDQTQ- 495
CRK34 KRYKIIRGIARGILYLHEDSRLRIIHRDLKASNILLDHEMNPKIADFGMARLVLMDQTQ- 494
CRK35 MRYQTIRGIARGILYLHEDSQLRIIHRDLKASNILLDENMYPKISDFGMARLVQVDQTQ- 490
CRK36 TRFKIIAGTARGILYLHQDSRLRIIHRDLKAGNILLDEDMNPKIADFGLARLFVVDQTH- 507
CRK37 TRFKIIAGTARGILYLHQDSRLRIIHRDLKAGNILLDEGMNPKIADFGLARLFVVDQTH- 501
CRK38 ERYKIIEGIAKGILYLHDHSRLKVIHRDLKPSNILLDENMNPKISDFGLARIVEKDQQE- 500
CRK39 TRYKIIKGIARGLLYLHQDSRLRIIHRDLKASNILLDKEMIPKISDFGLARLITADQTQ- 495
```

```
CRK40 TRYKIIIGIARGLLYLHQDSRLRIIHRDLKASNILLDKEMIPKISDFGIARLMTAGQTQ- 499
CRK41 QRFNIILGTAEGLAYLHEGTKIRIIHRDIKSSNVLLDDNLTPKIADFGLVRCFGADKSH- 482
CRK42 LRLRIINGIARGLLYLHEDSRLRVIHRDLKASNVLLDHDMNPKISDFGLARAFEIGQNQ- 481
CRK43 LRLSIIHGIARGILYLHEDSQLRVIHRDLKASNVLLDHDMNPKISDFGLARAFEGGQNQ- 507
CRK44 LRLSIINGIARGILYLHEDSRLKVIHRDLKASNVLLDDEMNPKISDFGLARAFEIGENQ- 467
CRK45 LRLSIINGIARGILYLHEDSRLKVIHRDLKASNVLLDDEMNPKISDFGLARAFEIGQNQ- 479
CRK46 LRLSIIHGIAKGLLYLHEDSRLKVIHRDLKASNVLLDDEMNPKISDFGLARSFEKGQNQ- 509


CRK1  -I-STAIAGTLGYMAPEYLAHGQLTEKADVYSFGVLLLEMVTGRQNNRSKVSEY---SDS 534
CRK2  -I-STAIAGTLGYMAPEYLAHGQLTEKADVYSFGVLLLEMATGRQNNRSKESDD---SDS 534
CRK3  -L-STAICGTLGYMAPEYVVLGKLTEKADVYSFGVLIMEIISGKKSTSFV--QN---SYS 531
CRK4  -GNTDRIVGTYGYMAPEYAMHGEFSVKSDVYSFGVLLMEIISGKKNSSFYQTDG--A-ED 567
CRK5  -I-STVIAGTLGYMAPEYVIRGKLSEKADVYSFGVLVIEIVSGKRNSSFI--MN---SSS 543
CRK6  -I-TTAIAGTLGYMAPEYLAHGQLTEKADVYSFGVLLLEIITGRQNNRSKASEY---SDS 536
CRK7  -L-STGNSRTLGYMAPEYVSKGQLTEKVDIYAFGVLIVEIVCGKKNSDHI--PG---STS 512
CRK8  -L-STGIAGTLGYMAPEYLIQGQLTDKADVYSFGVLVLETASGRKNNVFR--ED---SDS 541
CRK9  -ESTDLIVGTCGYMSPEYAMFGQFSEKSDVFSFGVMILEIITGRKNMHSSEPRR--IADG 787
CRK10 -ESTRRVMGTYGYMSPEYAMEGLYSVKSDVFSFGVLLLEIICGKRNSGFHLSEH--G-QS 554
CRK11 -ANTIRVVGTYGYMAPEYAMEGLFSVKSDVFSFGVLLLEIISGKRNSKFYLSDQ--G-QS 555
CRK12 ------------------------------------------------------------
CRK13 -L-CTKFAGTLGYTAPEYAMHGLLSEKADTYSYGIVVLEIISGQKSTDVKVDED--DSEY 534
CRK14 -L-STRFAGTMGYTAPEYALHGQLSEKADTYSYGIVVLEIISGQKSTDVKIVDDDAEDEY 539
CRK15 -L-STRFAGTLGYTAPEYAIHGQLSEKADTYSYGIVVLEIISGQKSTDVKGDED--GHEY 532
CRK16 -ANTSRIVGTYGYMAPEYAMHGQFSVKSDVFSFGVLILEIVSGQKNSGINNGEN--M-ED 545
CRK17 -ESTGRIVGTYGYMPPEYAMHGQFSVKSDVFSFGVMVLEIISGIRKCCSSESEV--I-DD 560
CRK18 -AETNRIVGTYGYMSPEYAMFGQFSEKSDVFSFGIMILEIITGKKNGNSYKSNE--DDGG 555
CRK19 -AETDRIVGTYGYMSPEYAMLGQFSEKSDVFSFGIIVLEIITSKKNARSYESHN--MEEG 550
CRK20 -GKTKRIVGTYGYMSPEYAMFGQFSEKSDIFSFGVMILEIVTGKKNINFHESQY--MPNG 544
CRK21 -GCTKRVVGTYGYMSPEYAMHGKFSDKSDVFSFGVMTLEIISGKKNSCSFESHR--V-DD 548
CRK22 -VQTGRIVGTYGYMSPEYAMRGQFSEKSDVFSFGILVLEIVSGKKNAGLYQSKH--A-DD 541
CRK23 -VNTGRIVGTYGYMSPEYAMRGQFSMKSDVFSFGVLVLEIVSGKKNTDFYQSNH--A-DD 543
CRK24 NTRTGRVVGTFGYMSPEYAMHGKFSVKSDVFSFGVLVLEIISGKKNSSYYRSHD--D-NE 555
CRK25 -GKTNRIVGTYGYMSPEYAMYGNFSEKSDVFSFGVIMLEIVSGKKNSRHVFSD---H-ED 547
CRK26 -GSTNRIVGTYGYMSPEYAMHGQFSEKSDVYSYGVIVLEIISSKKNSCSLLSDD--V-DD 546
CRK27 -ANTSRVVGTYGYMAPEYVIHGQFSVKSDVFSFGVLVLEIVSGQKNSWVQRGEN--E-GD 540
CRK28 -ANTSRIVGTYGYMAPEYAMHGQFSVKSDVFSFGVLILEIVSGQKNSGINKGEN--M-ED 565
CRK29 -VNTNRIVGTYGYMAPEYALHGQFSVKSDVFSFGVLILEIVSGQKNGGISNREN--M-DD 549
CRK30 -ANTNRVVGTYGYMAPEYIMQGQFSVKSDIFSFGVLLLEIVSGQKNSGFRHGEN--V-ED 557
CRK31 -ANTSRIVGTYGYMAPEYAMQGHFSVKSDVFSFGVLILEIVSGQKNSGINNGEN--M-ED 553
CRK32 -VNTNRIVGTYGYMAPEYALHGQFSVKSDVFSFGVLILEIVSGQKNGGISNREN--M-DD 565
CRK33 -ANTSRIVGTYGYMAPEYAMHGHFSVKSDVFSFGVLILEIVSGQKNSGINNGEN--V-ED 551
CRK34 -ANTSRIVGTYGYMAPEYVMHGQFSVKSDVFSFGVLILEIVSGQKNSGINNGEN--M-ED 550
CRK35 -ANTHRIVGTYGYMAPEYAIYGQFSTKSDVFSYGVLVLEIVSGRKSIGSRHGER--V-ED 546
CRK36 -EDTQRVVGTYGYMAPEYALHGHFSEKSDVFSFGVLVLEIVSGKKISSIQHGEE--S-GD 563
CRK37 -EDTQRVVGTFGYMAPEYALHGQFSEKSDVFSFGVLVLEIVSGQKISSIQHGEE--T-GD 557
CRK38 -GSTKRITGTYGYMSPEYAVFGQFSEKLDVYSFGVMVLEIISGRKNIGSYEPHR--IVNC 557
CRK39 -ENTSRVVGTFGYMAPEYIMQGQFSVKSDVFSFGVLVLEIVSGQKNHGVRDEKN--G-ED 551
CRK40 -ENTSRVVGTFGYMAPEYIMQGQFSVKSDVFSFGVLVLEIVSGQKNHGVRDEKN--G-EG 555
CRK41 -L-STGIAGTLGYMAPEYLIRGQLTDKADVYSFGVLVLEVVCGRRNNVFR--ED---SGS 535
CRK42 -GNTKRVMGTYGYMAPEYAMQGLFSVKSDVFSFGVLVLETICGRKNNGFYLSKH--G-QT 537
CRK43 -ANTKRVMGTYGYMAPEYAMEGLFSVKSDVFSFGVLVLEIICGRKNGGFYMSD---G-QT 562
CRK44 -AKTKRVVGTYGYMAPEYAMEGLFSVKSDVFSFGVLVLEIICGRKNSGFHRSEH--G-QS 523
CRK45 -ANTKRVVGTYGYMAPEYAMEGLFSVKSDVFSFGVLVLEIICGRKNSGFHRAEH--G-QS 535
CRK46 -ANTRRVMGTYGYMAPEYAMEGLFSVKSDVFSYGVLVLEIICGKKNSGFYLSEC--G-QS 565


CRK1  LVTV-AWKHFQAGTAEQLFDPNLVLQEDHNSEVKDEILRVVHIGLLCTQEVPSLRPIMSK 593
CRK2  LVTV-AWKHFQAGTAEQLFDPNLVLQEDHNIEVKDEILRGVHIGLLCTQEVPSLRPIMSK 593
CRK3  ILHRPLWIKQAM*----------------------------------------------- 544
CRK4  LLSY-AWLLWKDGTPLELMDPILRES-----YNQNEIIRSIHIGLLCVQEDPEDRPTMAT 621
CRK5  LLQT-VWRLYGSNRLSEIVDPTLECP-----FPAEEACQLLQIGLLCAQATAELRPSMSV 597
CRK6  LIIM-TWKHFQSGTAEQIIDPYLLLDDNHRSKVKNDILRVVQIGLLCTQEISSLRPSMSK 595
CRK7  LLHS-VWKNYKTNNITASVDPALHGK-----LTVEEASNTLQTGLLCTQSTVTLRPSMSE 566
CRK8  LLQT-VWKLYQSNRLGEAVDPCLGNE-----FPAREASRVFQIGLLCTQASASLRPSMAQ 595
CRK9  LLNY-VWKQWRDQTPLSILNSNIKEN-----YAEIEVIKCIQIGLLCVQENPDVRPTMVT 841
CRK10 LLVH-SWRLWGEGKSLEMLDPMLEKT-----YKGSEVMKCIHIGLLCVQEDAADRPTMST 608
CRK11 LLVY-AWNLWCESKGLELMDPSIEKS-----SVHSEVVKCMQIGLLCVQEDAADRPTMSS 609
CRK12 ------------------------------------------------------------
CRK13 LLQR-SWKLYERGSHLELLEEAIDPN----EYDAEEVKRIIEIALLCTQATAATRPTMSE 589
CRK14 LLRK-AWKLNERGMHLELVDKSLDPN----SYDAEEVKKVLDIALLCTQPSAAMRPAMSE 594
CRK15 LLQR-TWKLYERGTHLELVDKAIEPN----EYDAEDVKKIIEIALLCTQASAATRPTMSE 587
CRK16 LLNF-AWRNWKEGKAINIVDPSLNS------NSRNEMLRCIHIGLLCVQENLVDRPTMAN 598
CRK17 IRKH-AWTKWTKETPLELLDPHVGGP-----YSCEEVIKYIHIGLLCVQEDPNDRPTMAT 614
```

```
CRK18 LMSN-VWRNWTNQTPLSILEPNMKEN-----YFETEVIRCIEIGLLCVQENPNMRPTIAE 609
CRK19 LMTY-VWRHWKNETPMSILDPNMKEN-----YYETEVIRCIQIGLLCVQENPNIRPTMAT 604
CRK20 LLSY-VWRKWKEKTQLSIIDPLLEEN-----YSKNEVIKCIHISLLYVQEKKSIRPTIAD 598
CRK21 LLSY-AWNNWRDESPFVLLDPALEES-----YCPNEVEKCIQIGLLCVQENPDERPIIGT 602
CRK22 LLTL-AWKHWTEETPMEFLDPTLRGS-----YSRNEVNRCIHIGLLCVQESPSDRPSMAT 595
CRK23 LLIF-AWKKWTEQTTMEFLDPALRGS-----CSRNEVNRCIHIGLLCVQENPSDRPSMAT 597
CRK24 LLSF-AWKNWIDQTPFQILDPKLRGS-----YSRNEVQRCIHIALLCVEENPVERPSMAT 609
CRK25 LLSY-AWDQWRDQTPINILDQNIKES-----CNVSEVVKCIQIGLLCVQEKPQDRPTMTQ 601
CRK26 LLSD-AWRYWRDETPLKILDQNIEES-----SNHNEVLKCIQIGLLTYHNNLNALLSNLS 600
CRK27 LLTY-TWQTWRKRTMSNIVDPTIS-------GSSNEIMRCIHIALLCVQEKGDDRPTMAS 592
CRK28 LLSF-AWRNWKEGKAINIVDPSLNN------NSRNEMLRCIHIGLLCVQENLVDRPTMAN 618
CRK29 LLSF-AWRNWKEGKAINIVDPSLNS------NSRNEMLKCIHIGLICVQENLVDRPTMAN 602
CRK30 LLSF-TWRNWRDGTAVNIVDPSLEN------NSRNEVMRCIHIGLLCVQENLTDRPTMAT 610
CRK31 LLSF-AWRNWKEGKAINIVDPSLNS------NSRNEMLRCIHIGLLCVQENLVDRPNMTN 606
CRK32 LLSF-AWRNWKEGKAINIVDPSLNS------NSRNEMLRCIHIGLLCVQENLVDRPNMTN 618
CRK33 LLSF-AWRNWKEGKAIYIVDPSLNS------NSRNEMLRCIHIGLLCVQENLVDRPTMAN 604
CRK34 LLSF-AWRSWKEGKAINVVDPSLNN------NSRNEMLRCIHIGLLCVQENLVDRPTMAN 603
CRK35 LLSI-TWRNWKDGTIANIVDPTLIK------GSQNEIMRCIHIGLLCVQENMDARPTMTS 599
CRK36 LRHI-AWRSWREGRATDIVDPTLNN------GSESEIMRCMHIGLLCVQDSVAARPTMAS 616
CRK37 LRHI-AWRSWREGRATDIVDQTLND------GSESEIMRCIHIGLLCVQDNVAARPTMAS 610
CRK38 LLNF-VWEHWRDETPLSTLDPRLKEN-----HSNIEVIRCVKIGLLCVQENPDVRPTMLT 611
CRK39 LLSF-VWRNWIEGTVTNIIDPTLKN------SSQNEMIRCIGIGLLCVEEDLNNRPTMAN 604
CRK40 LLSF-VWRNWREGTVTNIMDPTLNN------SSLDEMIRCIGIGLLCVEEDLNNRPTMAN 608
CRK41 LLQT-VWKLYRSNTLTEAVDPCLGDD-----YPATEASRVFRIGLLCTQASASLRPSISQ 589
CRK42 LLLY-VSHPFGLI---NQISTILAN*---------------------------------- 559
CRK43 LLVY-AWRIWYEGKCLELMDPVLEKS-----FIGDEVERCIQIGLLCVQEDARDRPTMSD 616
CRK44 LLLY-AWSIWCAGKCLELMDPALIKS-----FIASEVVKCLHIGLLCVQQDAADRPTMST 577
CRK45 LLLY-AWSIWCAGKCLELMDPALIKS-----FIASEVVKCLHIGLLCVQQDAADRPTMST 589
CRK46 LTLY-AWKVWCAGKCLELMDPVLEEC-----CVESEVVKCIHIGLLCVQEDAADRPTMST 619


CRK1  TLQMLTKKE-ENLIAPSNPPFLDENTMELHDTSGDP----------------FYPLN-AA 635
CRK2  TLQMLTKKE-ENLIAPSNPPFLDKNTMELHDTNGDP----------------FYPLN-ES 635
CRK3  ------------------------------------------------------------
CRK4  IVLMLDS-NTVTLPTPKRPAFFIHSGTD-ATMPK--GQ-Q----------FDQSITKSLP 666
CRK5  VVKMV-NHN-HEIPQPTQPPFINSGSSELSRSGLPGHN--------------FQPESNTQ 641
CRK6  ALKMLTKKE-EHVEAPSNPPFIDESTMELHDQNDGP----------------FYPLN-AA 637
CRK7  VVQMLTKKD-YAIPSPKQKPFLNFSGLSQNDRTVSSKGLASARSSFHSTTSSLIPNDDST 625
CRK8  VACMLSNSD-LDVPIPKQPPFLNSRLLNQTPPLGFSTD-NSSSNTFKKIGVSYSPTQSSS 653
CRK9  IVSYLDG-HFSELPTPQKPAFFLHGRMDSKSIGR-------------KSSSRKSMNISTP 887
CRK10 VVVMLAS-DTMALSNPNHPAFSVGRKTKEEE---------------------STSKGSKD 646
CRK11 VVHMLAS-DTMSLPNPTRPAFSVGRTVIEQE---------------------SSSNTSMQ 647
CRK12 ------------------------------------------------------------
CRK13 VVVLLKSKNSVEHMRPTMPVFVGTNVMTREGNSTS------------------------- 624
CRK14 VVALLSSNDLLEHMRPTMPLFIESKLRPHRDIFASIGS----------------------S 633
CRK15 IVVLLKSKSLVEHLRPTMPVFVATNMKTRESKSSSTSG----------------------S 626
CRK16 IVLMLNS-YSLSLPIPAEPAFYMNSRTRSLPEMQSWDY-NS-----RETGSSEPILKSAQ 651
CRK17 IVFYLNN-HSINLPSPNEPGYFKSNRKDNMATNE--------------------ELDNII 653
CRK18 VVSYLND-LTIELPSPQEPAFFSHGIDQKAVMQQ-------------ESSSNSSVIGSMP 655
CRK19 VVSYLNN-HSLELPSPQEPAFFLHGIDKK-VMQQ-------------GSSSTISANSSMP 649
CRK20 VVSYLDGRHTLEFPSPQEPAFFLEDKMDTKT-------------------------STEH 633
CRK21 IVSYLNN-TSVQVPIPLEPAFFMHGRVRRHSTEH--QP-SS-----EFTL-KRHWNE--L 650
CRK22 IALMLNS-YSVTMSMPRQPASFLRGRSP-NRLNP--GL-DS-----DQSTTNQSTTSSIP 645
CRK23 IALMLNS-YSVTLSMPRQPASFLRGRGP-NRLNQ--GL-ES-----DQSTTDQFTSCSVP 647
CRK24 IMLALNA-YSVTLGLPRKPAFFVRGRATTDRLRQ--QL-DS-----DH-----SNTSSVP 655
CRK25 VVSYLSG-SLSELSLPEKPINCNQSGIVQRML-------------------VGGSSSGSA 641
CRK26 SNTDIDY-GFYNFSYGQDSSTVNAIGLCRGDV-------------------K-PDACRRC 639
CRK27 VALMLNS-HSVTLPLPSKPAFSIDSTSLSVIQ----------------------SNEPNI 629
CRK28 IVLMLNS-YSLSLPIPAEPAFYMNSRTRSLPEMQSWEY-NS-----REPGSSEAIFKSVQ 671
CRK29 IMLMLNS-YSLSLPIPGEPAFYINSRTRSLPETQSWEY-NS-----RETGSREAKLISAQ 655
CRK30 IMLMLSS-YSLGLPIPSEPAFYANSTARSLPATSSWGH-SS-----RATA-----NQSAQ 658
CRK31 IMLMLNS-YSLSLPIPAEPAFYMNSRTRSLPEMQSWDY-NS-----RETGSSEPILKSAQ 659
CRK32 IMLMLNS-YSLSLPIPAEPAFYMNSRTRSLPEMQSWDY-NS-----RETGSSEAILKSAQ 671
CRK33 IVLMLNS-YSLSLPIPAEPAFYMNSITRSLPEMQSWDY-NS-----RETRSSESIRKSAQ 657
CRK34 IVLMLNS-YSLSLPIPAEPAFYMNSRTRSLPETQSWEY-NS-----RETGSSEAILKSAQ 656
CRK35 VMLMLNS-YSLTLPVPSEPAFVVDSRTRSFPNMLSLEH-NS-----RETGSSESTNKSSQ 652
CRK36 VVPMLNS-HSFSLQVPLAPAFYGDAMSGIFEDMKLWEI-NS-----GTTRSIESTNRNDQ 669
CRK37 VVSMLNS-PSLSLQVPMAPAFYGNAMSGIFEDMQLREI-NS-----GTTRSNESTNRKDQ 663
CRK38 IVSYLNG-HLVELPSPVEPTFSLNRETNPIVA-Y-------------E------SNLRQS 650
CRK39 VALMLNS-CSITLPVPKKPAFFIDSATGSLPNMSWED--S------WPTRGTQSSGRSAQ 655
CRK40 VALMLNS-CSITLPVPKKPAFFIDSATGSLPNMSSED--S------WATKGTQSSGISAQ 659
CRK41 VVYMLTNSN-EDVPTPNQPPFLSTGMLDSDGSKSYTTN-SFISNALKKIGVSYIYSESSS 647
CRK42 ------------------------------------------------------------
CRK43 VVVMLAS-DTVAIPKPKHPAFSIGRTS-EEV---------------------STSRSSKN 653
```

```
CRK44 VVLMLGS-DTMDLPKPNHPAFSVGKLTSKEA---------------------SASENSKN 615
CRK45 VVLMLGS-ESMALPKPNHPAFSVGKLTSKEA---------------------SASRSTKN 627
CRK46 VVVMLAS-DMMSLPKPNQPAFSVGRMTLDDA---------------------STSKSSKN 657


CRK1  DSIATMSRSSFYPR*--------------------------------------------- 650
CRK2  DSIATMSRSSIYPR*--------------------------------------------- 650
CRK3  ------------------------------------------------------------
CRK4  MSINDVSISEMDPR*--------------------------------------------- 681
CRK5  SSANTMTESQIEPR*--------------------------------------------- 656
CRK6  DSLATMSHSSFYPR*--------------------------------------------- 652
CRK7  VLENSFSHSDMFIAGSPDSNIQINM--RAPEPR*-------------------------- 657
CRK8  SC------SLIRSSKTEETILEV*------------------------------------ 671
CRK9  LSINEMSTSECIPR*--------------------------------------------- 902
CRK10 PSVNDVTISNILPR*--------------------------------------------- 661
CRK11 YSVNEVTVSEVIPR*--------------------------------------------- 662
CRK12 ------------------------------------------------------------
CRK13 SSNATASISIASAR*--------------------------------------------- 639
CRK14 ASNGTASDSIVPA*---------------------------------------------- 647
CRK15 SSNATASISVLSAR*--------------------------------------------- 641
CRK16 ESENEVSITELYPR*--------------------------------------------- 666
CRK17 DSINEISLTKFFPR*--------------------------------------------- 668
CRK18 FSVNEMSISDFYPR*--------------------------------------------- 670
CRK19 FSVNEMPTTSFYPR*--------------------------------------------- 664
CRK20 FSVNEMSISTYYPR*--------------------------------------------- 648
CRK21 MDIGKN*----------------------------------------------------- 657
CRK22 WSVNEVSITELYPR*--------------------------------------------- 660
CRK23 WSVNEVSLTEVYPR*--------------------------------------------- 662
CRK24 FSAGDSLITEVYPR*--------------------------------------------- 670
CRK25 LSKNEMSVTIFIPR*--------------------------------------------- 656
CRK26 LNDSKVLLTQLCPNQKEAIGWYDQCMLRYSNRSLFNTMETSHSFALWNPGNASDIDQFNL 699
CRK27 QSVNEASITEPFAR*--------------------------------------------- 644
CRK28 ESENEASISELYPR*--------------------------------------------- 686
CRK29 ESENEASITELYPR*--------------------------------------------- 670
CRK30 ESENENSITEPYPR*--------------------------------------------- 673
CRK31 ESENEASITELYPR*--------------------------------------------- 674
CRK32 ESENEASITELYPR*--------------------------------------------- 686
CRK33 ESENEASITELYPR*--------------------------------------------- 672
CRK34 ESENEASITELYPR*--------------------------------------------- 671
CRK35 YSVDEASITETYPR*--------------------------------------------- 667
CRK36 DSLNEASITDPYPR*--------------------------------------------- 684
CRK37 DSLSEASITEPYPR*--------------------------------------------- 678
CRK38 TSNNEMSVSKLYPR*--------------------------------------------- 665
CRK39 ESVNEASITELYPR*--------------------------------------------- 670
CRK40 ESVNGASITELYPR*--------------------------------------------- 674
CRK41 SR------NSDGPSRSEESIVQV*------------------------------------ 665
CRK42 ------------------------------------------------------------
CRK43 LSINNITSSITLPR*--------------------------------------------- 668
CRK44 RSINDVTVSAVLAR*--------------------------------------------- 630
CRK45 LSINDVTVSTDLAR*--------------------------------------------- 642
CRK46 LSINDVTVSNIFPR*--------------------------------------------- 672


CRK1  ------------------------------------------------------------
CRK2  ------------------------------------------------------------
CRK3  ------------------------------------------------------------
CRK4  ------------------------------------------------------------
CRK5  ------------------------------------------------------------
CRK6  ------------------------------------------------------------
CRK7  ------------------------------------------------------------
CRK8  ------------------------------------------------------------
CRK9  ------------------------------------------------------------
CRK10 ------------------------------------------------------------
CRK11 ------------------------------------------------------------
CRK12 ------------------------------------------------------------
CRK13 ------------------------------------------------------------
CRK14 ------------------------------------------------------------
CRK15 ------------------------------------------------------------
CRK16 ------------------------------------------------------------
CRK17 ------------------------------------------------------------
CRK18 ------------------------------------------------------------
CRK19 ------------------------------------------------------------
CRK20 ------------------------------------------------------------
CRK21 ------------------------------------------------------------
```

```
CRK22 ------------------------------------------------------------
CRK23 ------------------------------------------------------------
CRK24 ------------------------------------------------------------
CRK25 ------------------------------------------------------------
CRK26 VLGNLLDSLIGQATTLGDSQRKFAAANVSESVFQTIYGLVQCTPDLSEQDCSECLTGAIS 759
CRK27 ------------------------------------------------------------
CRK28 ------------------------------------------------------------
CRK29 ------------------------------------------------------------
CRK30 ------------------------------------------------------------
CRK31 ------------------------------------------------------------
CRK32 ------------------------------------------------------------
CRK33 ------------------------------------------------------------
CRK34 ------------------------------------------------------------
CRK35 ------------------------------------------------------------
CRK36 ------------------------------------------------------------
CRK37 ------------------------------------------------------------
CRK38 ------------------------------------------------------------
CRK39 ------------------------------------------------------------
CRK40 ------------------------------------------------------------
CRK41 ------------------------------------------------------------
CRK42 ------------------------------------------------------------
CRK43 ------------------------------------------------------------
CRK44 ------------------------------------------------------------
CRK45 ------------------------------------------------------------
CRK46 ------------------------------------------------------------


CRK1  ------------------------------------------------------------
CRK2  ------------------------------------------------------------
CRK3  ------------------------------------------------------------
CRK4  ------------------------------------------------------------
CRK5  ------------------------------------------------------------
CRK6  ------------------------------------------------------------
CRK7  ------------------------------------------------------------
CRK8  ------------------------------------------------------------
CRK9  ------------------------------------------------------------
CRK10 ------------------------------------------------------------
CRK11 ------------------------------------------------------------
CRK12 ------------------------------------------------------------
CRK13 ------------------------------------------------------------
CRK14 ------------------------------------------------------------
CRK15 ------------------------------------------------------------
CRK16 ------------------------------------------------------------
CRK17 ------------------------------------------------------------
CRK18 ------------------------------------------------------------
CRK19 ------------------------------------------------------------
CRK20 ------------------------------------------------------------
CRK21 ------------------------------------------------------------
CRK22 ------------------------------------------------------------
CRK23 ------------------------------------------------------------
CRK24 ------------------------------------------------------------
CRK25 ------------------------------------------------------------
CRK26 EIPQCCDGKRGGRILRPSCNFRYEIYRFYDLTDVKIPPAPAPAPKVSVLPPLSTDTLSTE 819
CRK27 ------------------------------------------------------------
CRK28 ------------------------------------------------------------
CRK29 ------------------------------------------------------------
CRK30 ------------------------------------------------------------
CRK31 ------------------------------------------------------------
CRK32 ------------------------------------------------------------
CRK33 ------------------------------------------------------------
CRK34 ------------------------------------------------------------
CRK35 ------------------------------------------------------------
CRK36 ------------------------------------------------------------
CRK37 ------------------------------------------------------------
CRK38 ------------------------------------------------------------
CRK39 ------------------------------------------------------------
CRK40 ------------------------------------------------------------
CRK41 ------------------------------------------------------------
CRK42 ------------------------------------------------------------
CRK43 ------------------------------------------------------------
CRK44 ------------------------------------------------------------
CRK45 ------------------------------------------------------------
CRK46 ------------------------------------------------------------
```

```
CRK1  ------------------------------------------------------------
CRK2  ------------------------------------------------------------
CRK3  ------------------------------------------------------------
CRK4  ------------------------------------------------------------
CRK5  ------------------------------------------------------------
CRK6  ------------------------------------------------------------
CRK7  ------------------------------------------------------------
CRK8  ------------------------------------------------------------
CRK9  ------------------------------------------------------------
CRK10 ------------------------------------------------------------
CRK11 ------------------------------------------------------------
CRK12 ------------------------------------------------------------
CRK13 ------------------------------------------------------------
CRK14 ------------------------------------------------------------
CRK15 ------------------------------------------------------------
CRK16 ------------------------------------------------------------
CRK17 ------------------------------------------------------------
CRK18 ------------------------------------------------------------
CRK19 ------------------------------------------------------------
CRK20 ------------------------------------------------------------
CRK21 ------------------------------------------------------------
CRK22 ------------------------------------------------------------
CRK23 ------------------------------------------------------------
CRK24 ------------------------------------------------------------
CRK25 ------------------------------------------------------------
CRK26 VQVENDDEIRSSETLQLDFSTIMTATNNFSDANRLGQGGFGPVYKGKLSNGQDVAVKRLC 879
CRK27 ------------------------------------------------------------
CRK28 ------------------------------------------------------------
CRK29 ------------------------------------------------------------
CRK30 ------------------------------------------------------------
CRK31 ------------------------------------------------------------
CRK32 ------------------------------------------------------------
CRK33 ------------------------------------------------------------
CRK34 ------------------------------------------------------------
CRK35 ------------------------------------------------------------
CRK36 ------------------------------------------------------------
CRK37 ------------------------------------------------------------
CRK38 ------------------------------------------------------------
CRK39 ------------------------------------------------------------
CRK40 ------------------------------------------------------------
CRK41 ------------------------------------------------------------
CRK42 ------------------------------------------------------------
CRK43 ------------------------------------------------------------
CRK44 ------------------------------------------------------------
CRK45 ------------------------------------------------------------
CRK46 ------------------------------------------------------------

CRK1  ------------------------------------------------------------
CRK2  ------------------------------------------------------------
CRK3  ------------------------------------------------------------
CRK4  ------------------------------------------------------------
CRK5  ------------------------------------------------------------
CRK6  ------------------------------------------------------------
CRK7  ------------------------------------------------------------
CRK8  ------------------------------------------------------------
CRK9  ------------------------------------------------------------
CRK10 ------------------------------------------------------------
CRK11 ------------------------------------------------------------
CRK12 ------------------------------------------------------------
CRK13 ------------------------------------------------------------
CRK14 ------------------------------------------------------------
CRK15 ------------------------------------------------------------
CRK16 ------------------------------------------------------------
CRK17 ------------------------------------------------------------
CRK18 ------------------------------------------------------------
CRK19 ------------------------------------------------------------
CRK20 ------------------------------------------------------------
CRK21 ------------------------------------------------------------
CRK22 ------------------------------------------------------------
CRK23 ------------------------------------------------------------
CRK24 ------------------------------------------------------------
CRK25 ------------------------------------------------------------
```

```
CRK26 KNSLQGDIEFKNEVMLVAKLQHRNLVKLIGFCLERRERLLVYEFVPNKSLDFFIFEYVFR 939
CRK27 ------------------------------------------------------------
CRK28 ------------------------------------------------------------
CRK29 ------------------------------------------------------------
CRK30 ------------------------------------------------------------
CRK31 ------------------------------------------------------------
CRK32 ------------------------------------------------------------
CRK33 ------------------------------------------------------------
CRK34 ------------------------------------------------------------
CRK35 ------------------------------------------------------------
CRK36 ------------------------------------------------------------
CRK37 ------------------------------------------------------------
CRK38 ------------------------------------------------------------
CRK39 ------------------------------------------------------------
CRK40 ------------------------------------------------------------
CRK41 ------------------------------------------------------------
CRK42 ------------------------------------------------------------
CRK43 ------------------------------------------------------------
CRK44 ------------------------------------------------------------
CRK45 ------------------------------------------------------------
CRK46 ------------------------------------------------------------


CRK1  ------------------------------------------------------------
CRK2  ------------------------------------------------------------
CRK3  ------------------------------------------------------------
CRK4  ------------------------------------------------------------
CRK5  ------------------------------------------------------------
CRK6  ------------------------------------------------------------
CRK7  ------------------------------------------------------------
CRK8  ------------------------------------------------------------
CRK9  ------------------------------------------------------------
CRK10 ------------------------------------------------------------
CRK11 ------------------------------------------------------------
CRK12 ------------------------------------------------------------
CRK13 ------------------------------------------------------------
CRK14 ------------------------------------------------------------
CRK15 ------------------------------------------------------------
CRK16 ------------------------------------------------------------
CRK17 ------------------------------------------------------------
CRK18 ------------------------------------------------------------
CRK19 ------------------------------------------------------------
CRK20 ------------------------------------------------------------
CRK21 ------------------------------------------------------------
CRK22 ------------------------------------------------------------
CRK23 ------------------------------------------------------------
CRK24 ------------------------------------------------------------
CRK25 ------------------------------------------------------------
CRK26 GQFSVKSDVYSFGVLVLEIVTDQKNSWACRGENEGDLLTYTWQNWREGTVSNIIDPTIID 999
CRK27 ------------------------------------------------------------
CRK28 ------------------------------------------------------------
CRK29 ------------------------------------------------------------
CRK30 ------------------------------------------------------------
CRK31 ------------------------------------------------------------
CRK32 ------------------------------------------------------------
CRK33 ------------------------------------------------------------
CRK34 ------------------------------------------------------------
CRK35 ------------------------------------------------------------
CRK36 ------------------------------------------------------------
CRK37 ------------------------------------------------------------
CRK38 ------------------------------------------------------------
CRK39 ------------------------------------------------------------
CRK40 ------------------------------------------------------------
CRK41 ------------------------------------------------------------
CRK42 ------------------------------------------------------------
CRK43 ------------------------------------------------------------
CRK44 ------------------------------------------------------------
CRK45 ------------------------------------------------------------
CRK46 ------------------------------------------------------------


CRK1  ------------------------------------------------------------
CRK2  ------------------------------------------------------------
CRK3  ------------------------------------------------------------
```

```
CRK4  ------------------------------------------------------------
CRK5  ------------------------------------------------------------
CRK6  ------------------------------------------------------------
CRK7  ------------------------------------------------------------
CRK8  ------------------------------------------------------------
CRK9  ------------------------------------------------------------
CRK10 ------------------------------------------------------------
CRK11 ------------------------------------------------------------
CRK12 ------------------------------------------------------------
CRK13 ------------------------------------------------------------
CRK14 ------------------------------------------------------------
CRK15 ------------------------------------------------------------
CRK16 ------------------------------------------------------------
CRK17 ------------------------------------------------------------
CRK18 ------------------------------------------------------------
CRK19 ------------------------------------------------------------
CRK20 ------------------------------------------------------------
CRK21 ------------------------------------------------------------
CRK22 ------------------------------------------------------------
CRK23 ------------------------------------------------------------
CRK24 ------------------------------------------------------------
CRK25 ------------------------------------------------------------
CRK26 SSRNEIMRFIHIALLCVQEKVTDRPTMASVVLMLSSYSVSLPLPSKPAFSVRSRNVSVIQ 1059
CRK27 ------------------------------------------------------------
CRK28 ------------------------------------------------------------
CRK29 ------------------------------------------------------------
CRK30 ------------------------------------------------------------
CRK31 ------------------------------------------------------------
CRK32 ------------------------------------------------------------
CRK33 ------------------------------------------------------------
CRK34 ------------------------------------------------------------
CRK35 ------------------------------------------------------------
CRK36 ------------------------------------------------------------
CRK37 ------------------------------------------------------------
CRK38 ------------------------------------------------------------
CRK39 ------------------------------------------------------------
CRK40 ------------------------------------------------------------
CRK41 ------------------------------------------------------------
CRK42 ------------------------------------------------------------
CRK43 ------------------------------------------------------------
CRK44 ------------------------------------------------------------
CRK45 ------------------------------------------------------------
CRK46 ------------------------------------------------------------


CRK1  --------------
CRK2  --------------
CRK3  --------------
CRK4  --------------
CRK5  --------------
CRK6  --------------
CRK7  --------------
CRK8  --------------
CRK9  --------------
CRK10 --------------
CRK11 --------------
CRK12 --------------
CRK13 --------------
CRK14 --------------
CRK15 --------------
CRK16 --------------
CRK17 --------------
CRK18 --------------
CRK19 --------------
CRK20 --------------
CRK21 --------------
CRK22 --------------
CRK23 --------------
CRK24 --------------
CRK25 --------------
CRK26 SEEYDPVSGNSIN* 1073
CRK27 --------------
CRK28 --------------
CRK29 --------------
```

```
CRK30 --------------
CRK31 --------------
CRK32 --------------
CRK33 --------------
CRK34 --------------
CRK35 --------------
CRK36 --------------
CRK37 --------------
CRK38 --------------
CRK39 --------------
CRK40 --------------
CRK41 --------------
CRK42 --------------
CRK43 --------------
CRK44 --------------
CRK45 --------------
CRK46 --------------
```
