# Supplementary material for: Cysteine-Rich Receptor-Like Kinase Gene Family Identification in the Phaseolus Genome and Comparative Analysis of Their Expression Profiles Specific to Mycorrhizal and Rhizobial Symbiosis
Source: Genes (Basel). 2019 Jan 17;10(1):59. doi: 10.3390/genes10010059 (PMC6356535; doi:10.3390/genes10010059)
Supplement: Supplementary file 1 [file genes-10-00059-s001.zip › Supplementary Figures S1-S3, S5-S7.pptx]

## Slide 1
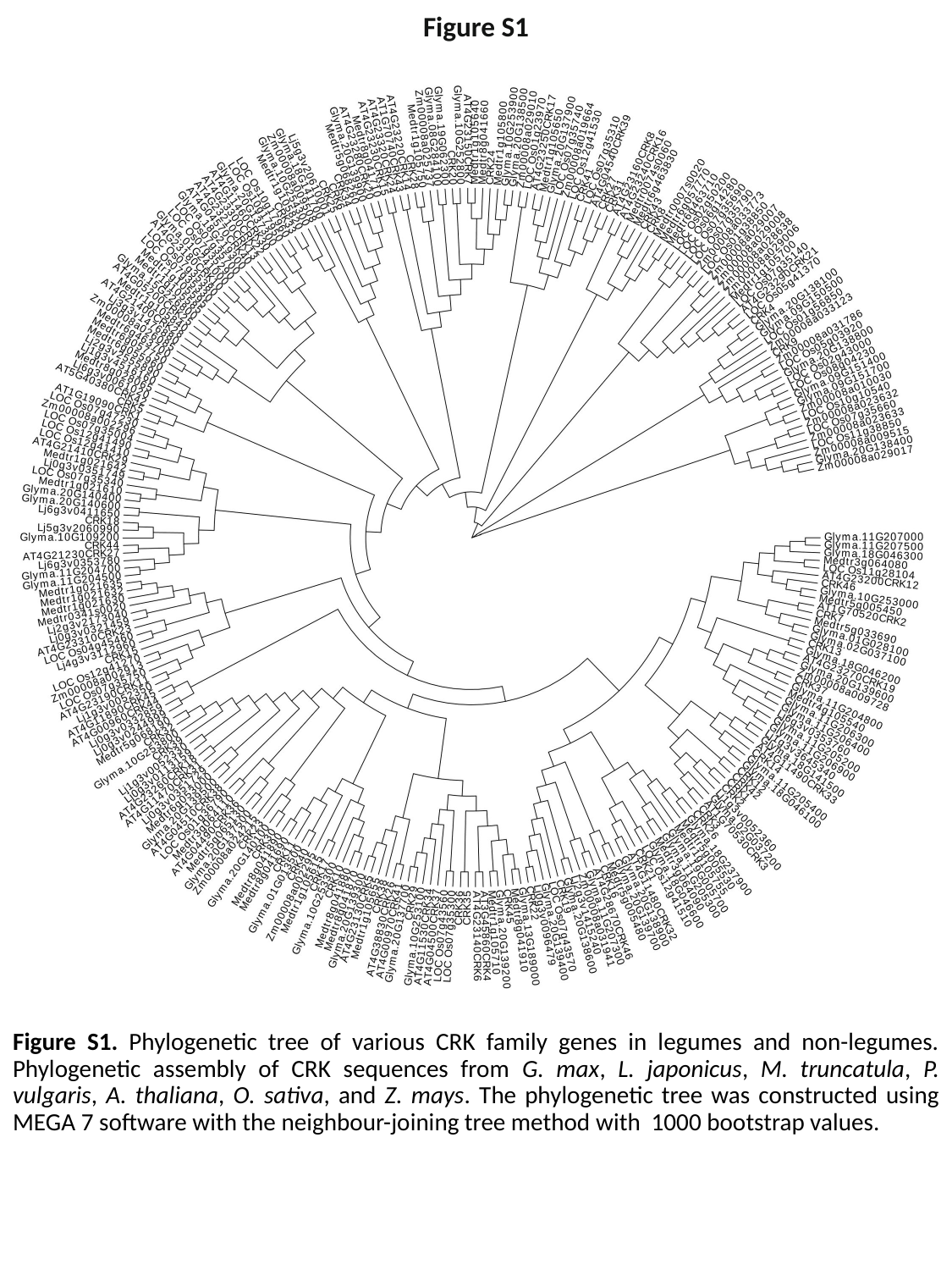

Figure S1
Figure S1. Phylogenetic tree of various CRK family genes in legumes and non-legumes. Phylogenetic assembly of CRK sequences from G. max, L. japonicus, M. truncatula, P. vulgaris, A. thaliana, O. sativa, and Z. mays. The phylogenetic tree was constructed using MEGA 7 software with the neighbour-joining tree method with 1000 bootstrap values.

## Slide 2
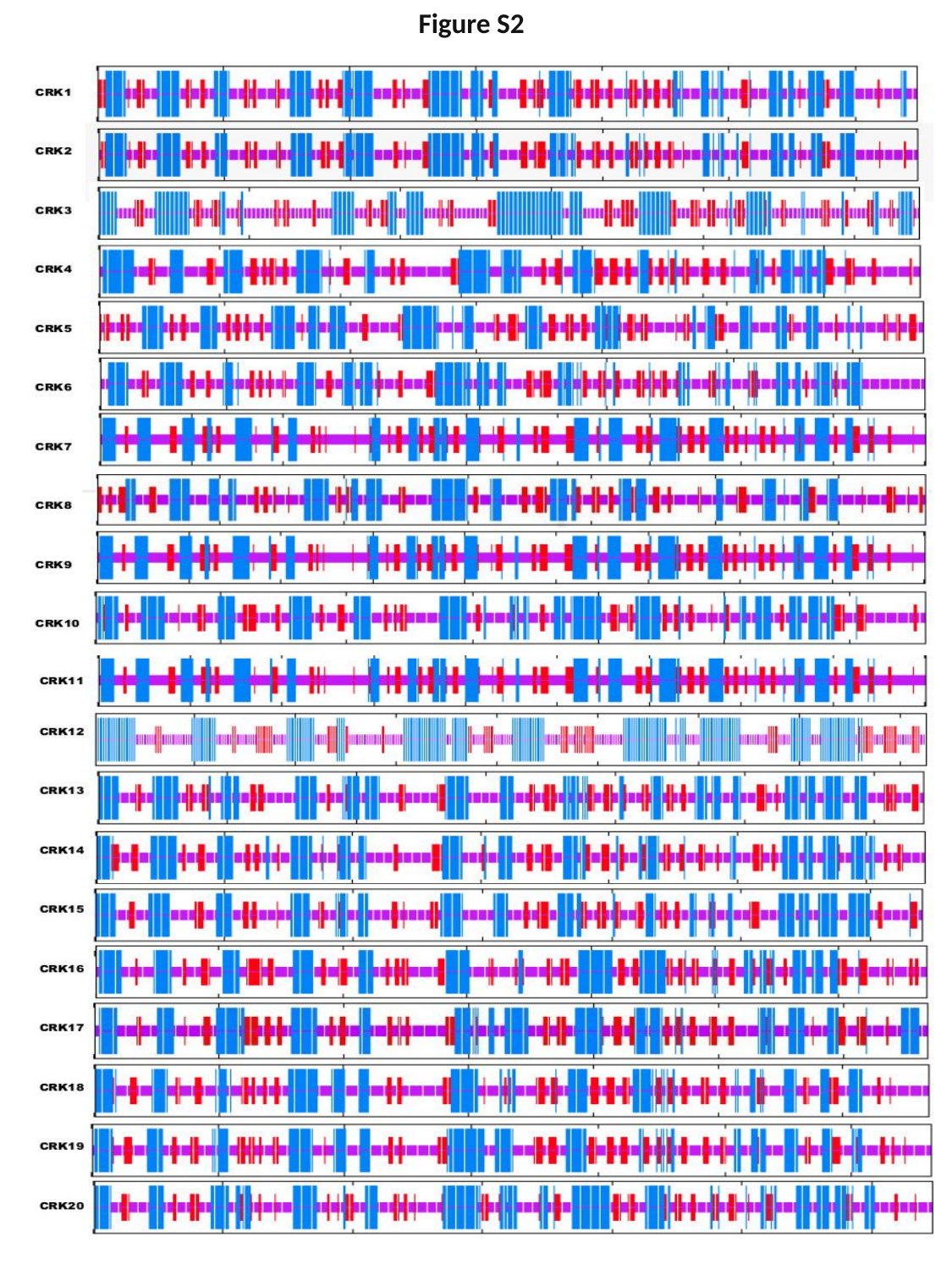

Figure S2

## Slide 3
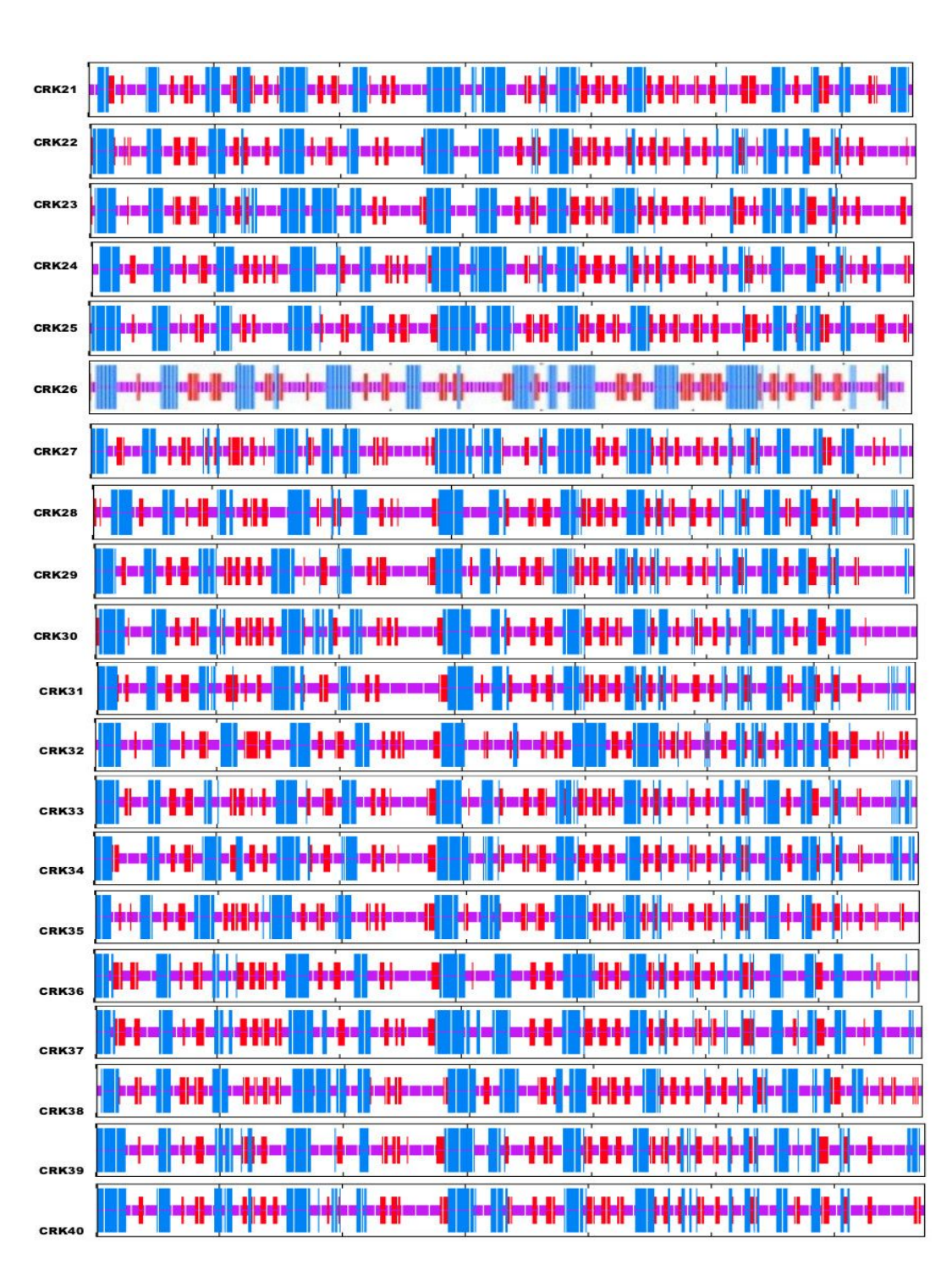

## Slide 4
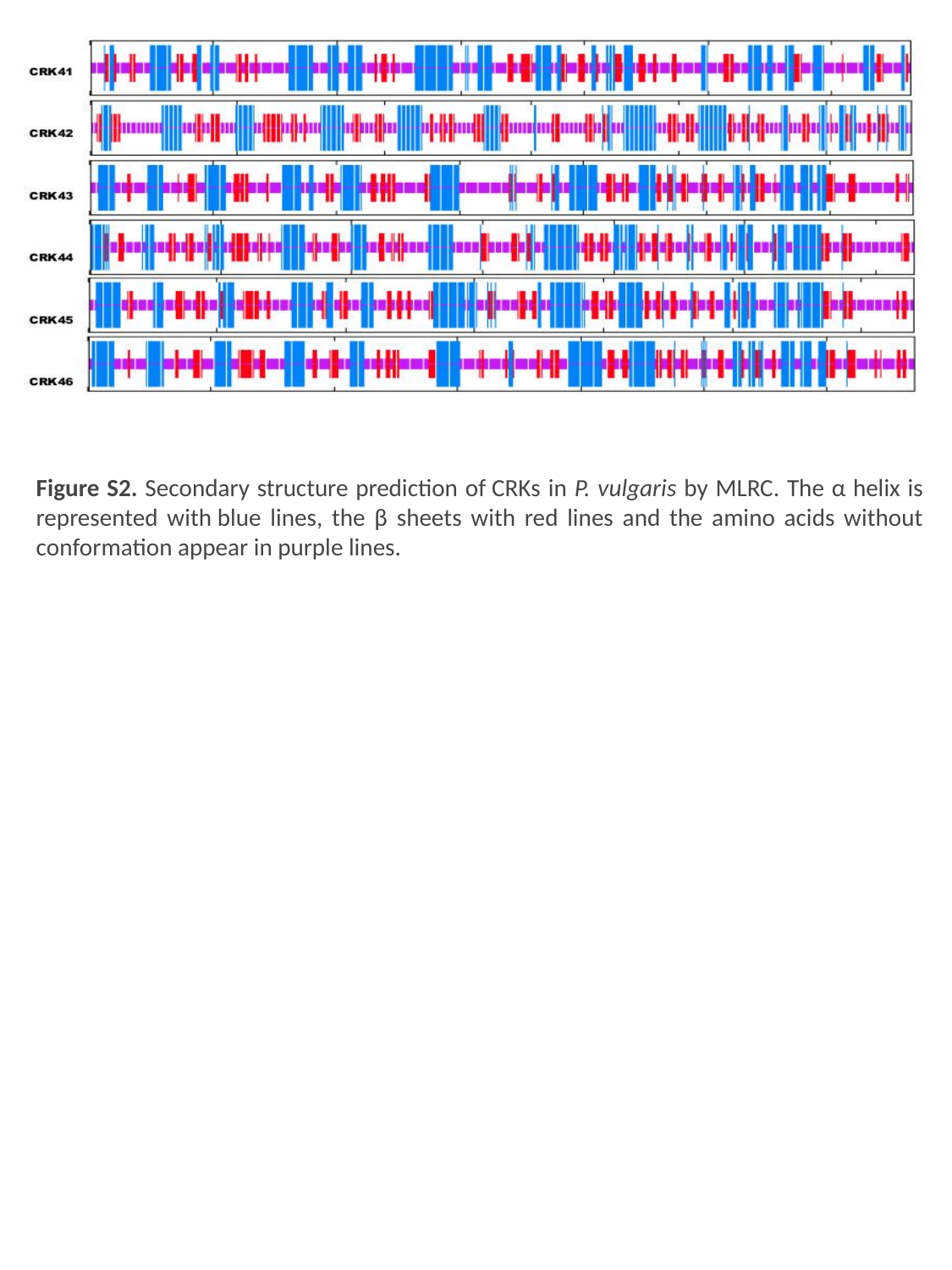

Figure S2. Secondary structure prediction of CRKs in P. vulgaris by MLRC. The α helix is represented with blue lines, the β sheets with red lines and the amino acids without conformation appear in purple lines.

## Slide 5
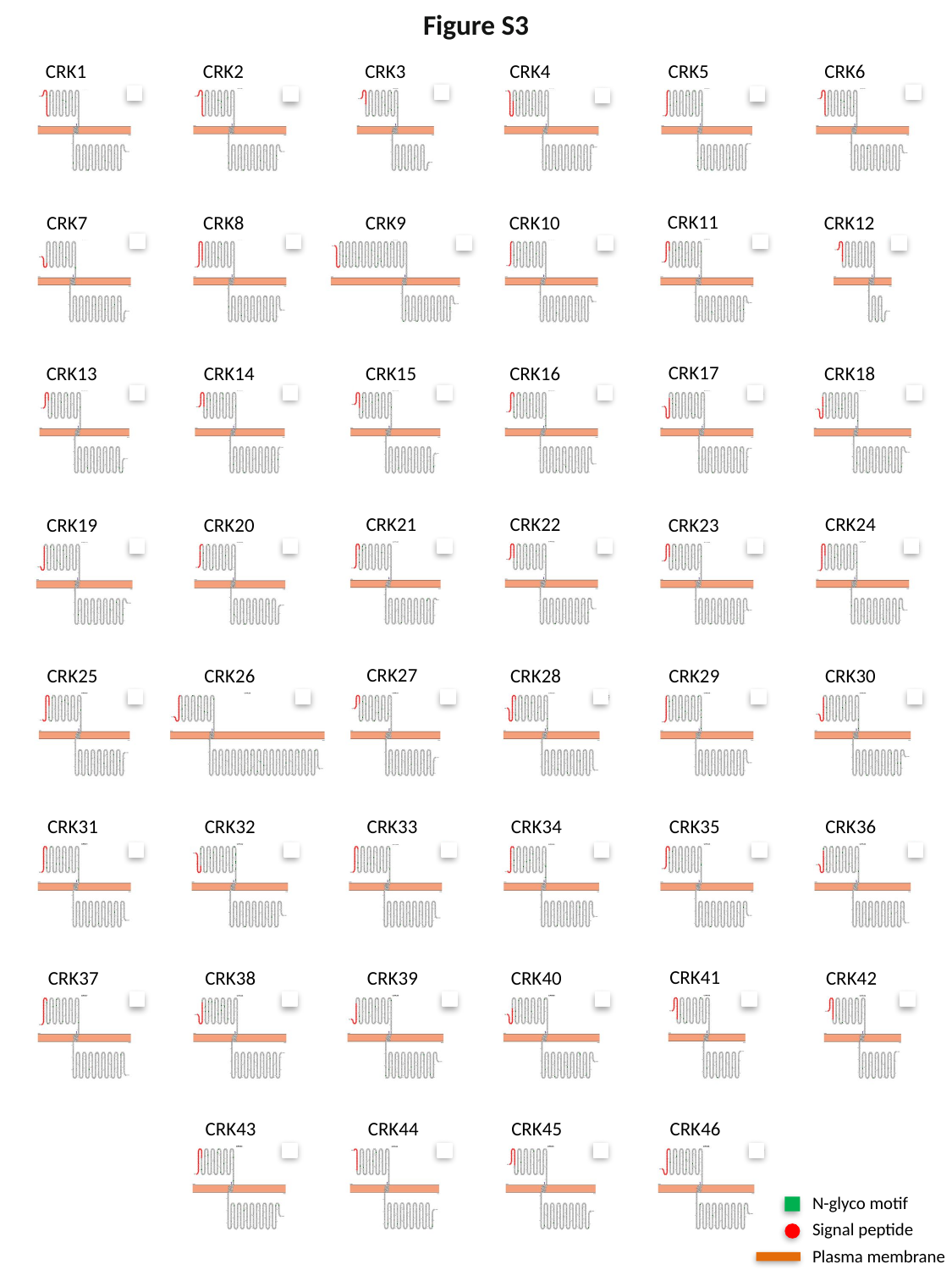

Figure S3
CRK3
CRK2
CRK6
CRK5
CRK4
CRK1
CRK11
CRK10
CRK9
CRK7
CRK8
CRK12
CRK17
CRK16
CRK15
CRK13
CRK14
CRK18
CRK22
CRK21
CRK24
CRK23
CRK19
CRK20
CRK27
CRK25
CRK26
CRK30
CRK29
CRK28
CRK31
CRK32
CRK36
CRK35
CRK34
CRK33
CRK41
CRK40
CRK39
CRK37
CRK38
CRK42
CRK43
CRK46
CRK45
CRK44
N-glyco motif
Signal peptide
Plasma membrane

## Slide 6
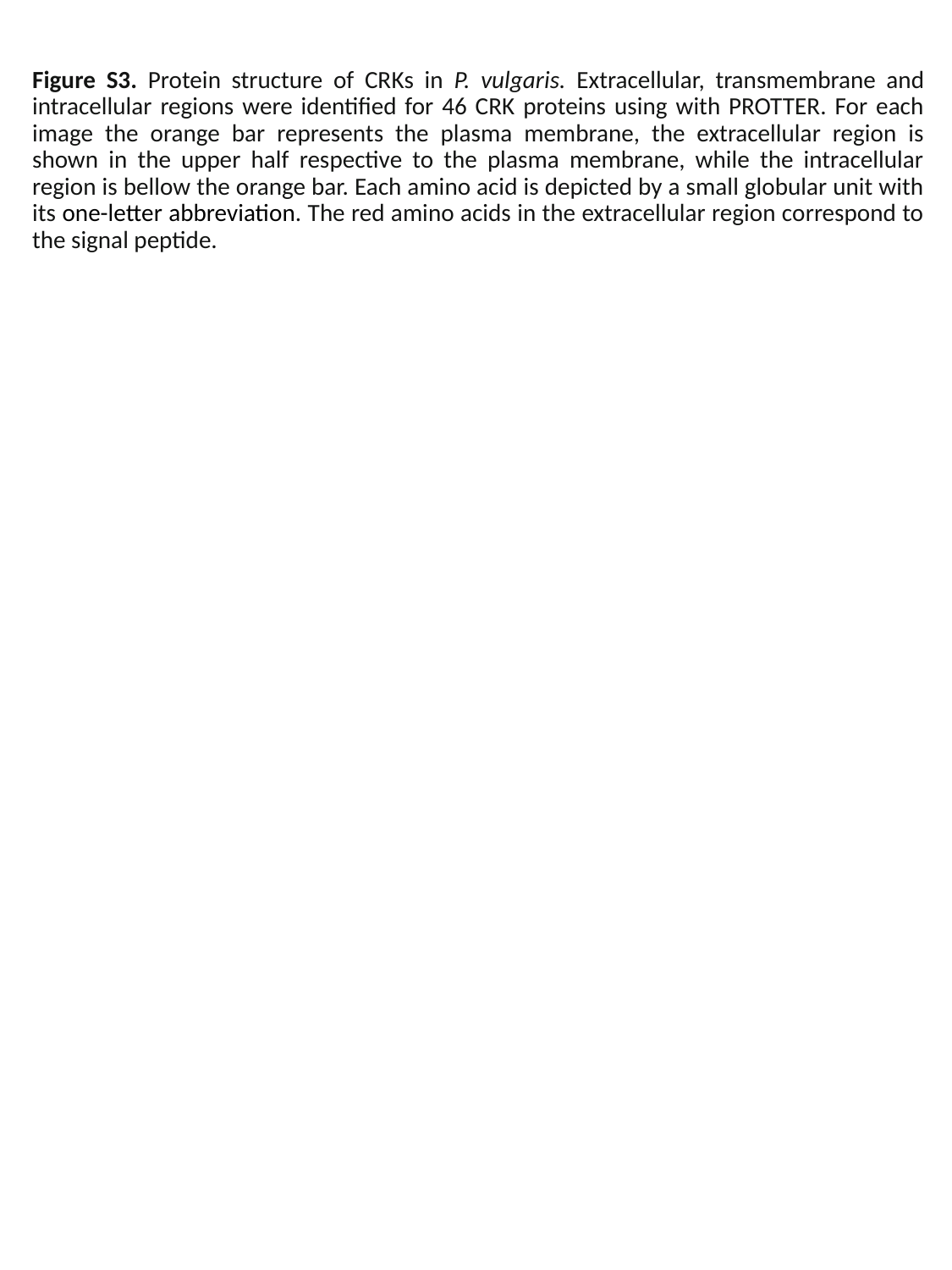

Figure S3. Protein structure of CRKs in P. vulgaris. Extracellular, transmembrane and intracellular regions were identified for 46 CRK proteins using with PROTTER. For each image the orange bar represents the plasma membrane, the extracellular region is shown in the upper half respective to the plasma membrane, while the intracellular region is bellow the orange bar. Each amino acid is depicted by a small globular unit with its one-letter abbreviation. The red amino acids in the extracellular region correspond to the signal peptide.

## Slide 7
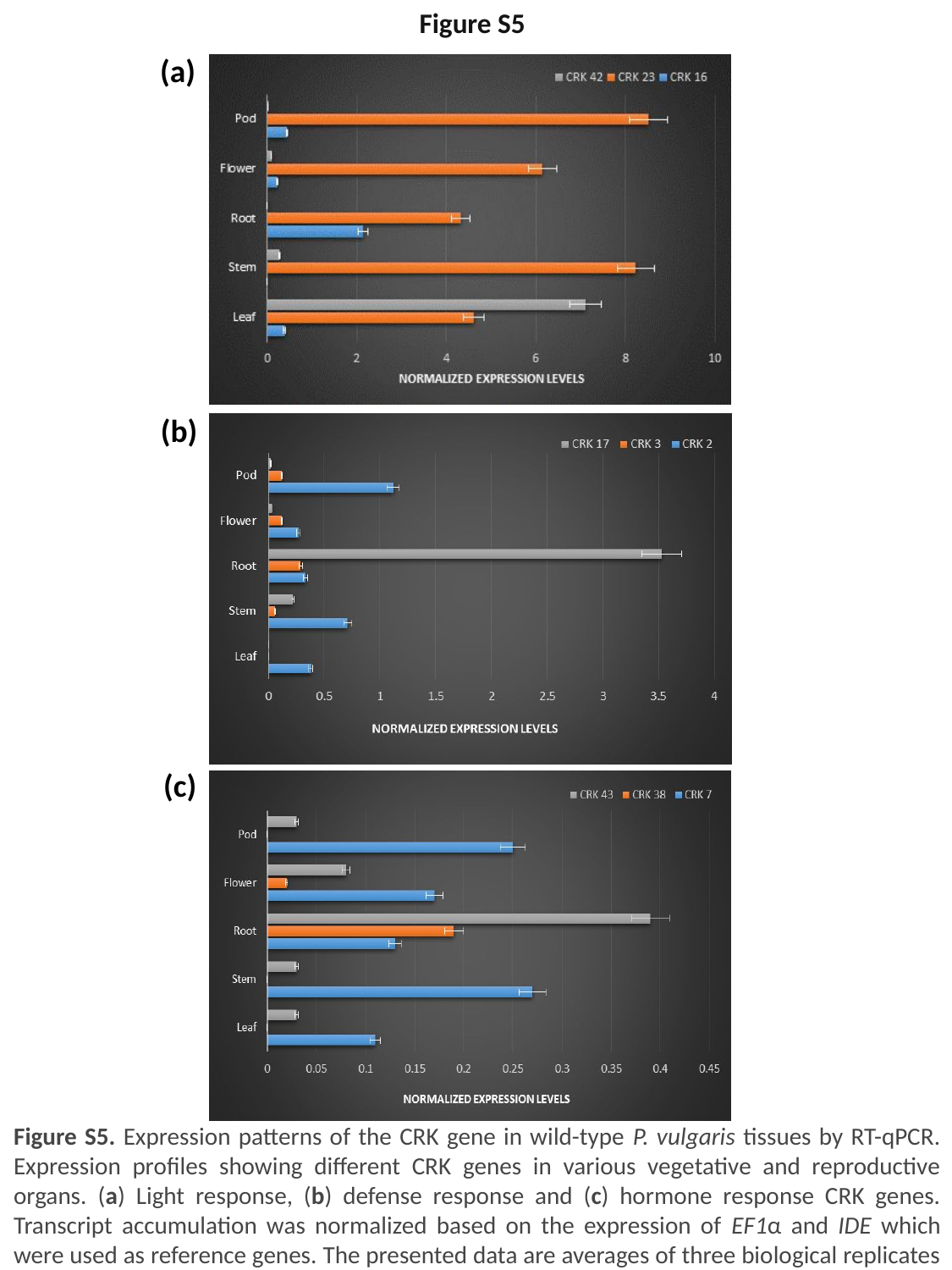

Figure S5
(a)
(b)
(c)
Figure S5. Expression patterns of the CRK gene in wild-type P. vulgaris tissues by RT-qPCR. Expression proﬁles showing different CRK genes in various vegetative and reproductive organs. (a) Light response, (b) defense response and (c) hormone response CRK genes. Transcript accumulation was normalized based on the expression of EF1α and IDE which were used as reference genes. The presented data are averages of three biological replicates (n > 9) and represent means ± SEM.

## Slide 8
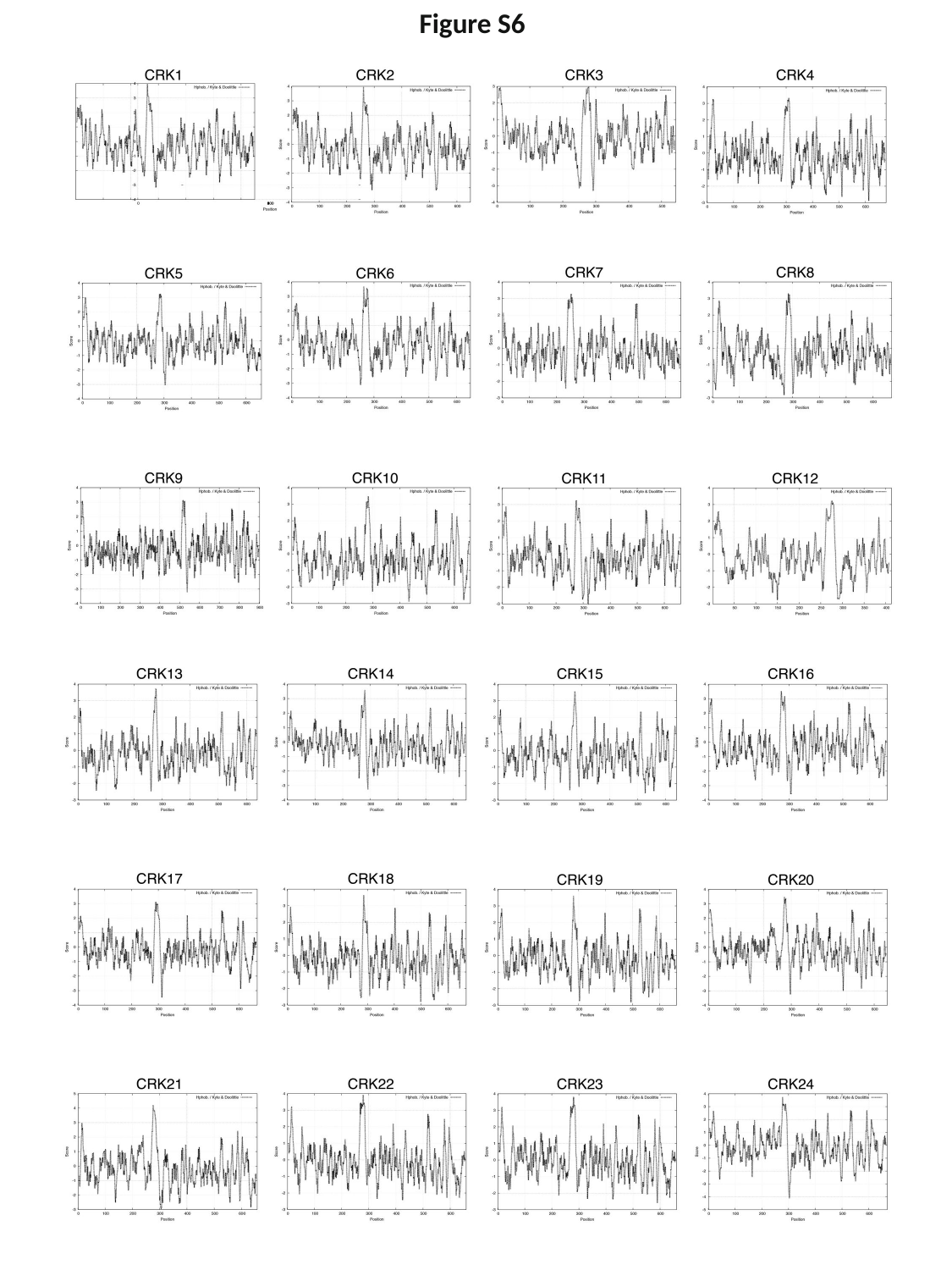

Figure S6

## Slide 9
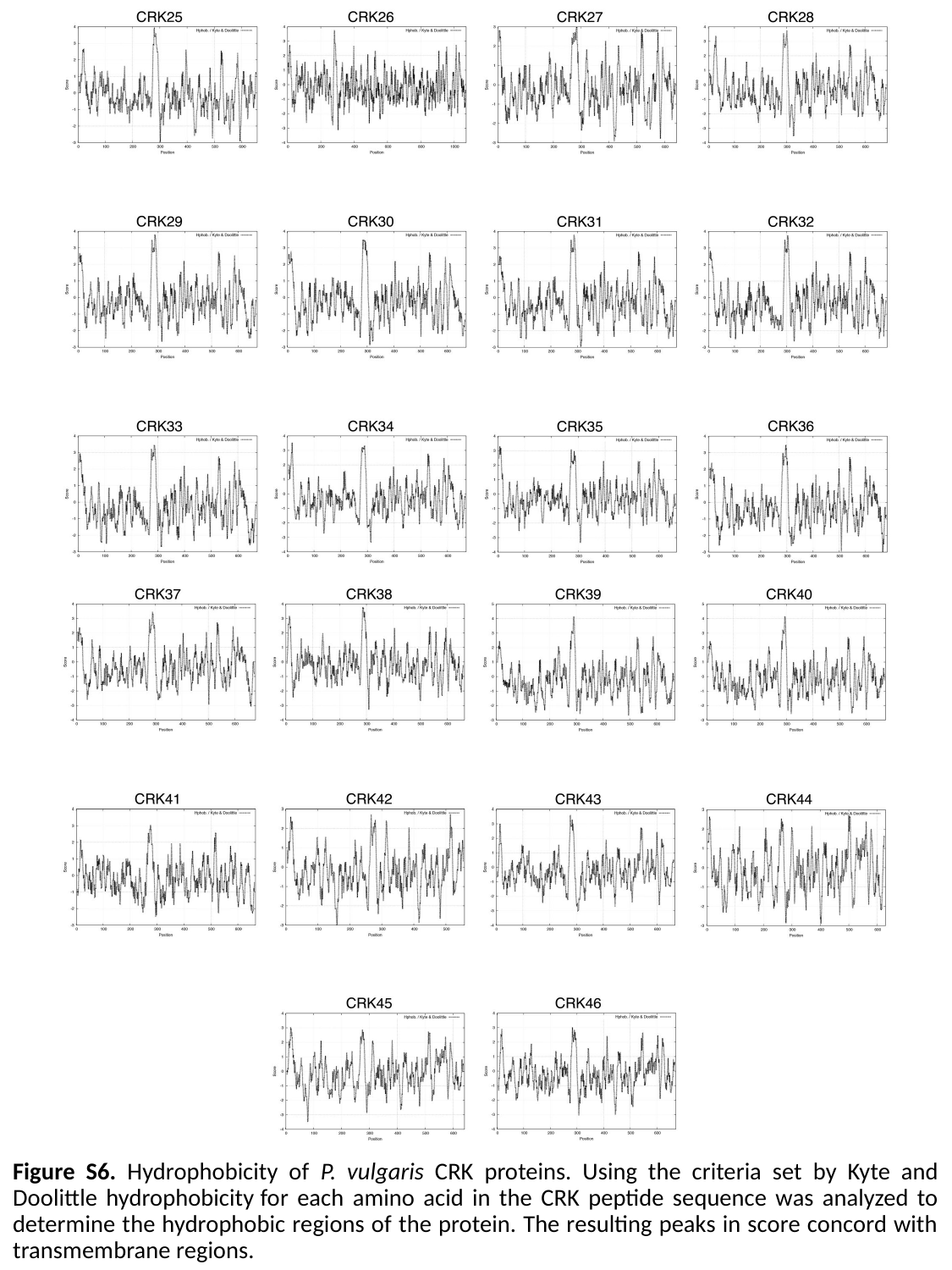

Figure S6. Hydrophobicity of P. vulgaris CRK proteins. Using the criteria set by Kyte and Doolittle hydrophobicity for each amino acid in the CRK peptide sequence was analyzed to determine the hydrophobic regions of the protein. The resulting peaks in score concord with transmembrane regions.

## Slide 10
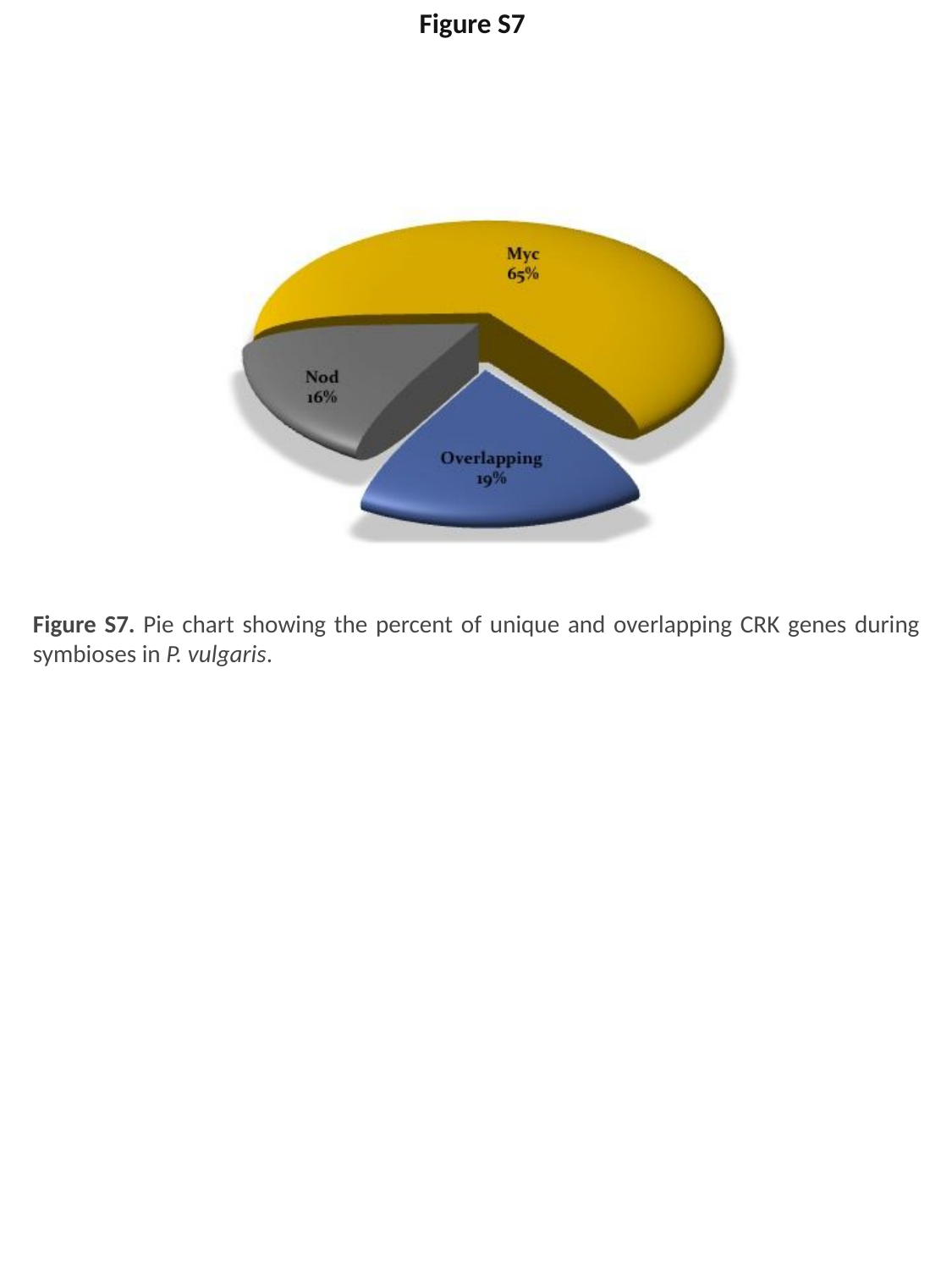

Figure S7
Figure S7. Pie chart showing the percent of unique and overlapping CRK genes during symbioses in P. vulgaris.
