# Supplementary material for: Cysteine-Rich Receptor-Like Kinase Gene Family Identification in the Phaseolus Genome and Comparative Analysis of Their Expression Profiles Specific to Mycorrhizal and Rhizobial Symbiosis
Source: Genes (Basel). 2019 Jan 17;10(1):59. doi: 10.3390/genes10010059 (PMC6356535; doi:10.3390/genes10010059)
Supplement: Supplementary file 1 [file genes-10-00059-s001.zip › Table S1.docx]

**Table S1.** Primer sequences of *P. vulgaris* genes used to perform RT-qPCR analyses.

| *P. vulgaris*  Locus Name^#^ | Gene | | Oligonucleotide sequence | Size of the amplicon (bp) |
| --- | --- | --- | --- | --- |
| Phvul.004G075100 | *PvElf1α* | F | 5′-GGTCATTGGTCATGTCGACTCTGG-3′ | 146 |
|  |  | R | 5′-GCACCCAGGCATACTTGAATGACC-3′ |  |
| Phvul.001G133200 | *PvIDE* | F | 5′-GCAACCAACCTTTCATCAGC-3′ | 156 |
|  |  | R | 5′-AGAAATGCCTCAACCCTTTG-3′ |  |
| Phvul.004G007300 | *PvPT*-4 | F | 5′-GCGGTGACTAACATGTTAGGG-3′ | 137 |
|  |  | R | 5′-CCTGTGCCCTAGTATTGTTGG-3′ |  |
| Phvul.001G019200 | *PvMYB73* | F | 5′- GAAACTGGGCCTTGATAAGCCG-3′ | 380 |
|  |  | R | 5′-GAGCCAAAGTTGTCGGAAAACG-3′ |  |
| Phvul.003G155600 | *PvENODL12* | F | 5′-GGATTCAGACACTTCATAAGTGG-3′ | 271 |
|  |  | R | 5′-GAAGTAACATCATCACCCCC-3′ |  |
| Phvul.002G063700 | *pvCRK2* | F | 5′-ACAGAACAACAGGAGCAAAG-3′ | 277 |
|  |  | R | 5′-GAAGGGTGGATTAGAGGGAG-3′ |  |
| Phvul.002G063600 | *pvCRK3* | F | 5′- GCCCCAAAAGGAGAAGTGAG-3′ | 164 |
|  |  | R | 5′- AGCAGGAAAGCCAAGGAAG-3′ |  |
| Phvul.003G202000 | *pvCRK7* | F | 5′- ACAACTACTCCTTCATCCGC-3′ | 278 |
|  |  | R | 5′- ACCCCTACCCTCAACATTC-3′ |  |
| Phvul.006G006800 | *pvCRK12* | F | 5′- GGAGCAGTTTACAAGGGTGG-3′ | 227 |
|  |  | R | 5′-GAGGACAGGTAGTAATCAAGGC-3′ |  |
| Phvul.007G052500 | *pvCRK16* | F | 5′- CCACCACCATCCACAATTAAC-3′ | 184 |
|  |  | R | 5′- CATCCTCCTCCTTTTCATCTTC-3′ |  |
| Phvul.007G051500 | *pvCRK17* | F | 5′- GCCATAGCAGACATTCCAAC-3′ | 254 |
|  |  | R | 5′- CAGCCCAAGGATAAAAGCAC-3′ |  |
| Phvul.007G050600 | *pvCRK23* | F | 5′- TCCGCCGAATCTGCTAAAC-3′ | 295 |
|  |  | R | 5′- AAACCACCCCAAATCCACC-3′ |  |
| Phvul.008G077800 | *pvCRK38* | F | 5′- CCTTCTTCAACTTCCCAACC-3′ | 202 |
|  |  | R | 5′- ATACCATACACGCTACTGTTTC-3′ |  |
| Phvul.011G193300 | *pvCRK42* | F | 5′- CTGTGTCTCTAATGCTGCC-3′ | 205 |
|  |  | R | 5′- GCCTCTCATAAAATCCTCACC-3′ |  |
| Phvul.011G194401 | *pvCRK43* | F | 5′- CCAATCCGCAAACAGATAAAC-3′ | 178 |
|  |  | R | 5′- TCTTCTTCTCTAACTCCATCCC-3′ |  |
|  |  |  |  |  |
|  |  |  |  |  |

^# Phytozome IDs^
